# Multiobjective starfish optimization algorithm for engineering design and optimal power flow problems

**DOI:** 10.1038/s41598-026-35329-4

**Published:** 2026-01-23

**Authors:** Mohammed Jameel, Hana Merah, Alaa M. Abd El-latif, Tareq M. Al-shami, A. Almutairi, Mohamed Abouhawwash

**Affiliations:** 1 Department of Mathematics, Sana’a University, Sana’a, Yemen; 2 LGEERE Laboratory, Department of Electrical Engineering, University of El-Oued, El-Oued, 39000 Algeria; 3 Department of Mathematics, College of Science, Northern Border University, Arar, 91431 Saudi Arabia; 4 Jadara University Research Center, Jadara University, Irbid, Jordan; 5 Department of Mathematics, College of Science, Qassim University, Buraydah, 51452 Saudi Arabia; 6 Department of Industrial and Systems Engineering, King Fahd University of Petroleum & Minerals, Dhahran, 31261 Saudi Arabia; 7 Interdisciplinary Research Center of Smart Mobility and Logistics (IRC-SML), King Fahd University of Petroleum & Minerals, Dhahran, 31261 Saudi Arabia

**Keywords:** Starfish optimization algorithm, Multi-objective starfish optimization algorithm (MOSFOA), Real-world problems, Optimal power flow, Engineering, Mathematics and computing

## Abstract

This paper presents a robust multi-objective optimization approach—the multi-objective starfish optimization algorithm (MOSFOA)—designed to address complex challenges in engineering design and optimal power flow analysis. As an advanced extension of the starfish optimization algorithm (SFOA), MOSFOA leverages biological inspiration from starfish behaviors such as exploration, predation, and regeneration to balance global exploration and local exploitation. The proposed MOSFOA employs elitist non-dominated sorting (NDS) and crowding distance (CD) mechanisms to preserve solution diversity and guide convergence toward the Pareto-optimal front. The effectiveness of MOSFOA is validated on standard ZDT and DTLZ benchmark suites and further demonstrated on real-world applications, including engineering design tasks and the IEEE 30-bus power system. Performance comparisons with ten state-of-the-art multi-objective algorithms, using metrics such as inverted generational distance (IGD) and hypervolume (HV), confirm the strength of MOSFOA in achieving a well-balanced trade-off between convergence and diversity. Additionally, the KKT proximity metric (KKTPM) is employed to assess convergence. The results demonstrate that MOSFOA significantly outperforms its counterparts in terms of both IGD and HV, achieving superior convergence and diversity performance. These findings underscore MOSFOA’s robustness, scalability, and stability across runs. Moreover, its strong performance in handling constrained engineering problems highlights its practical potential for real-world decision-making and optimization tasks in power systems and complex design optimization, making MOSFOA a promising tool for both theoretical research and industrial applications. Source code of MOSFOA are publicly available at https://www.mathworks.com/matlabcentral/fileexchange/183090-mosfoa-multi-objective-starfish-optimization-algorithm.

## Introduction

In recent decades, metaheuristic algorithms have become vital in solving complex optimization problems across various fields in science and engineering. Unlike conventional optimization techniques, which typically depend on gradient information and are constrained by problem-specific structures, metaheuristics offer greater flexibility by mimicking natural processes, such as biological evolution, swarm behavior, and physical dynamics^1^. These algorithms are particularly effective in large-scale and high-dimensional problem spaces, where conventional techniques often fail to provide satisfactory solutions^2^.

Classical metaheuristics, such as the Genetic Algorithm (GA)^3^, Particle Swarm Optimization (PSO)^4^, and Ant Colony Optimization (ACO)^5^, have demonstrated wide applicability in fields including electrical engineering, materials science, and machine learning. Their effectiveness stems from a well-maintained balance between exploration (broad search across the solution space) and exploitation (focused local improvement), which enables them to efficiently handle complex, real-world optimization problems that are often non-convex and multi-modal.

Optimization plays a fundamental role in engineering, where improving system efficiency, reducing costs, and enhancing performance are ongoing priorities. Optimization problems are typically classified into single-objective and multi-objective categories. Unlike classical differential-based methods, modern approaches increasingly rely on heuristic and metaheuristic strategies, especially when dealing with nonlinear and complex objective landscapes. These methods, when integrated with modern computational capabilities, have significantly contributed to advancements in robotics, material design, and other high-impact engineering applications. As the complexity of engineering systems continues to grow, so too does the demand for more refined and powerful optimization algorithms, where even marginal performance improvements can lead to substantial practical benefits.

Single-objective optimization (SOO) focuses on optimizing a single performance metric, such as minimizing cost or improving efficiency. Although this method is conceptually simple and has played a foundational role in many optimization studies, especially in cases where one objective clearly dominates, it often falls short when applied to real-world scenarios. These scenarios typically involve multiple, conflicting objectives that require a broader framework to properly capture the necessary trade-offs. Classical SOO techniques, including linear programming, quadratic programming, and metaheuristic-based approaches, have been widely explored and utilized in various scientific and engineering fields. However, their effectiveness diminishes when applied to multi-dimensional design spaces where balancing competing objectives is critical. To address increasingly complex problem landscapes, a range of recently developed SOO algorithms has been introduced, demonstrating strong capabilities in identifying optimal solutions. Notable examples include Secretary bird optimization algorithm (SBOA)^6^, Mantis Search Algorithm (MSA)^7^, Coyote and Badger Optimization (CBO)^8^, Snow ablation optimizer (SAO)^9^, Exponential distribution optimizer (EDO)^10^, Starfish Optimization Algorithm (SFOA)^11^, Hybrid Grasshopper Optimization Algorithm (HGOA)^12^, and Hyperbolic Sine Optimizer (HSP)^13^.

Recently, several researchers have raised critical concerns regarding the rapid proliferation of metaphor-based metaheuristic algorithms. While many of these algorithms demonstrate promising performance, studies such as “*Metaheuristics—The Metaphor Exposed*’’^14^ have argued that excessive reliance on metaphors may obscure methodological clarity and hinder theoretical understanding. This line of critique has been extended in recent works, including “*Exposing the Grey Wolf*,* Moth-Flame*,* Whale*,* Firefly*,* Bat*,* and Antlion Algorithms*’’^15^. These studies emphasize the need for greater methodological rigor, transparent validation, and theoretical grounding in the design of new algorithms. In response to these insights, the proposed MOSFOA is developed with a strong focus on both biological interpretability and mathematical soundness, aiming to address existing criticisms while maintaining practical efficiency in solving complex multi-objective optimization problems.

Multi-objective optimization (MOO) addresses the shortcomings of SOO by targeting the simultaneous optimization of two or more conflicting objectives. A typical example can be found in electrical engineering, especially in optimal power flow (OPF) problems, where objectives such as minimizing fuel costs and maximizing voltage stability or system reliability often conflict and must be balanced concurrently. MOO algorithms, especially metaheuristic-based ones such as MOPSO^16^, NSGA-II^17^, SPEA-II^18^, MOEA/D^19^, MONOA^20^, MOAVOA^21^, MOMSA^22^, and MOPO^23^, are specifically designed to handle such complexities. These algorithms are designed to produce a diverse set of Pareto-optimal (PO) solutions, allowing decision-makers to assess trade-offs and choose outcomes that best align with their goals and contextual requirements. However, despite these strengths, MOO remains computationally intensive, largely due to the difficulty of generating high-quality, well-distributed solutions that accurately reflect the complex balance among competing objectives. This inherent challenge underscores the ongoing demand for innovation in algorithm design to enhance convergence, scalability, and overall solution quality. Furthermore, the No-Free Lunch (NFL) theorem^24^ reinforces this need by asserting that no single optimization algorithm is universally superior across all problem domains, highlighting the importance of creating new, problem-specific optimization strategies.

Recently, Zhong et al.^11^ introduced the Starfish Optimization Algorithm (SFOA), a single-objective metaheuristic inspired by the natural behaviors of starfish. SFOA models essential biological actions—including exploration, foraging, and regeneration—within a two-phase framework. In the exploration phase, it employs a hybrid strategy that integrates five-dimensional and one-dimensional movement patterns to enhance search capability and computational efficiency. In the exploitation phase, it simulates predatory and regenerative behaviors to intensify the search around promising regions. Extensive evaluations on 65 benchmark functions, including the CEC 2017 and CEC 2022 suites, and comparisons with 100 metaheuristic algorithms demonstrated that SFOA is highly competitive, outperforming most state-of-the-art optimizers in terms of accuracy, efficiency, and scalability.

Given these promising characteristics, SFOA provides a solid foundation for further research and adaptation. Although a wide range of metaheuristic algorithms have been proposed in the literature, SFOA has attracted attention because of its unique biologically inspired mechanisms and proven effectiveness. These results motivate its selection as the basis for developing a robust multi-objective variant. To the best of our knowledge, no multi-objective version of SFOA has been reported in the literature. This work addresses that gap by extending its framework with Pareto-based elitist sorting and crowding distance mechanisms to effectively tackle MOO problems in both benchmark test suites and real-world applications. This study proposes the Multi-objective Starfish Optimization Algorithm (MOSFOA), a novel approach developed to enhance the performance of existing MOO techniques. MOSFOA is specifically designed to maintain an effective balance between exploration and exploitation, an essential factor for achieving accurate, globally optimal solutions in complex real-world scenarios^25^.

The primary contributions of this work are outlined below:

- Proposing MOSFOA, a novel multi-objective extension of the SFOA Algorithm.
- Validating MOSFOA on standard benchmark problems (ZDT, DTLZ) and engineering design cases, where it achieves superior Pareto front quality. Additionally, its application to the real-world speed reducer design problem further reinforces the algorithm’s effectiveness in addressing practical industrial optimization tasks.
- Applying MOSFOA to OPF (IEEE 30-bus) in single-, bi-, and tri-objective cases with improved convergence.
- Employing fuzzy set theory for decision support to identify best-compromise solutions.
- Demonstrating robustness and statistical superiority (IGD, HV, KKTPM, Wilcoxon, Friedman) compared with ten state-of-the-art algorithms.

The paper is organized into six main sections. Section 1 presents the research topic, while Sect. 2 describes the framework of MOO problems, including essential definitions and a review of existing literature on various solution strategies. Section 3 presents the formulation of the OPF problem. In Sect. 4, the original SFOA is described, along with its extension to handle multi-objective problems (MOPs), known as MOSFOA. Section 5 discusses the experimental outcomes. Lastly, Sect. 6 summarizes the main findings and suggests future research directions and possible applications of MOSFOA.

## Literature review

This section offers a brief overview of fundamental concepts in MOO, such as Pareto optimality, Pareto dominance, PO set, and PF. Following this, we review a range of MOO approaches, covering both classical methods and recently developed techniques.

### Definition of MOO

The structure of MOPs is generally framed as either a maximization or minimization task and can be represented in the following standard form:

1 $$\:\left\{\begin{array}{c}Min/Max:F\left(\mathbf{x}\right)=\left[{f}_{1},{f}_{2}\dots\:,\:{f}_{M}\right]\\\:{g}_{i}\left(\mathbf{x}\right)\le\:0,i=\mathrm{1,2},\dots\:,P\\\:{h}_{j}\left(\mathbf{x}\right)=0,j=\mathrm{1,2},\dots\:,K\\\:{lb}_{i}\le\:{x}_{i}\le\:{ub}_{i},\:i=\mathrm{1,2},...,\:N\end{array}\right.$$

where $\:N$ represents the number of decision variables, while $\:M$ refers to the number of objective functions. The problem may also include $\:P$ inequality constraints represented by $\:{g}_{i}\left(\mathbf{x}\right)\le\:0$, and $\:K$ equality constraints denoted as $\:{h}_{j}\left(\mathbf{x}\right)=0$. Each design variable $\:{x}_{i}$ is bounded within a specific range [$\:{lb}_{i}{,ub}_{i}$], representing its lower and upper limits, respectively.

In MOO, traditional relational operators (e.g., $\:\le\:,\:\ge\:$) are no longer sufficient to compare solutions due to the presence of multiple conflicting objectives. Instead, the concept of Pareto optimality is utilized to evaluate and rank solutions. The core definitions related to Pareto optimality are introduced below.

#### **Definition 1**

(*Pareto Dominance*,* Minimization Case*^26^: A solution $\:\mathbf{x}$ is said to dominate another solution $\:\mathbf{y}$ (denoted as $\:\mathbf{x}\:\prec\:\:\mathbf{y}$) if and only if the following two conditions are met:

- For all objective functions: $\:{f}_{i}\left(\mathbf{x}\right)\le\:{f}_{i}\left(\mathbf{y}\right),\forall\:i\in\:1,\dots\:,M,$
- There exists at least one objective function for which the inequality is strict$\::\exists\:j\in\:1,\dots\:,M$ such that $\:{f}_{j}\left(\mathbf{x}\right)<{f}_{j}\left(\mathbf{y}\right)$.

#### **Definition 2**

 ( *Pareto Optimality*)^26^: A solution **x** is considered PO (or non-dominated) if there is no other solution **y** in the search space such that **y** dominates **x**. The collection of all such non-dominated solutions forms the Pareto Set (PS), defined as:

$$\:PS\:=\:\{\mathbf{x}\:\in\:\:X\:|\:\nexists\:\:\mathbf{y}\:\in\:\:\boldsymbol{X}\:such\:that\:\mathbf{y}\:\prec\:\:\mathbf{x}\}.$$

**Fig. 1:**
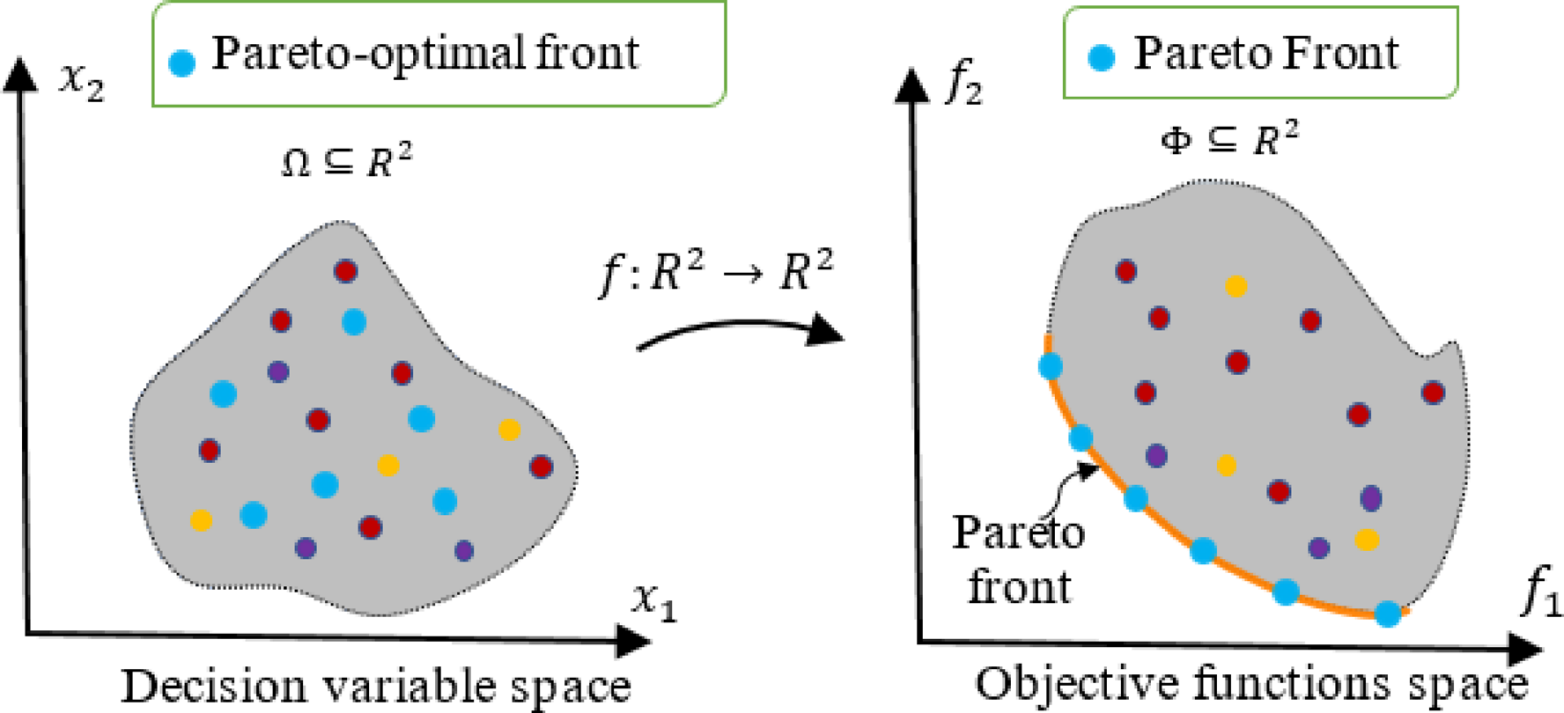
Illustration of decision and objective spaces in MOO.

#### **Definition 3**

(*Pareto Front*^26^ :

The PF is the mapping of the PS into the objective space using the objective function vector (*F* defined in Eq. (1)). It is defined as:

$$\:\hspace{1em}\hspace{1em}PF=F\left(\mathbf{x}\right)|\mathbf{x}\in\:P$$

Figure 1 illustrates the preceding definitions through a conceptual example involving two decision variables (forming a two-dimensional decision space) and two objective functions (forming a two-dimensional objective space), both considered under a minimization framework. In the figure, light blue circles represent PO solutions. These are referred to as PO decision vectors in the decision space and PO objective vectors in the objective space. The PF, comprising solely Non-Dominated (ND) solutions, may exhibit a variety of forms; it can be continuous or discrete, and its shape may be convex, concave, or a mix of both, depending on the specific characteristics of the optimization problem.

### Challenges of mops

In practical applications, solving MOPs is particularly challenging not just because multiple objectives must be optimized at once, but more so due to the inherent conflict among these objectives. Enhancing one objective often results in the degradation of another, making it impractical to simultaneously improve all objectives. Since the relative importance of each objective is usually unknown beforehand, the typical goal shifts to finding a diverse set of ND solutions that closely approximate the true Pareto front (PF). The final selection from this set is then made by the decision-maker, based on individual preferences. This aspect is referred to as convergence toward the true PF. However, convergence alone does not suffice to evaluate the effectiveness of an optimization method. A set of solutions that, despite lying on the PF, are clustered in a limited region or unevenly spread can hinder decision-making. In such cases, choosing one solution may significantly compromise the performance of others, reducing the practical utility of the results. This brings attention to the second critical aspect: diversity, ensuring a well-distributed spread of solutions across the entire PF.

**Fig. 2:**
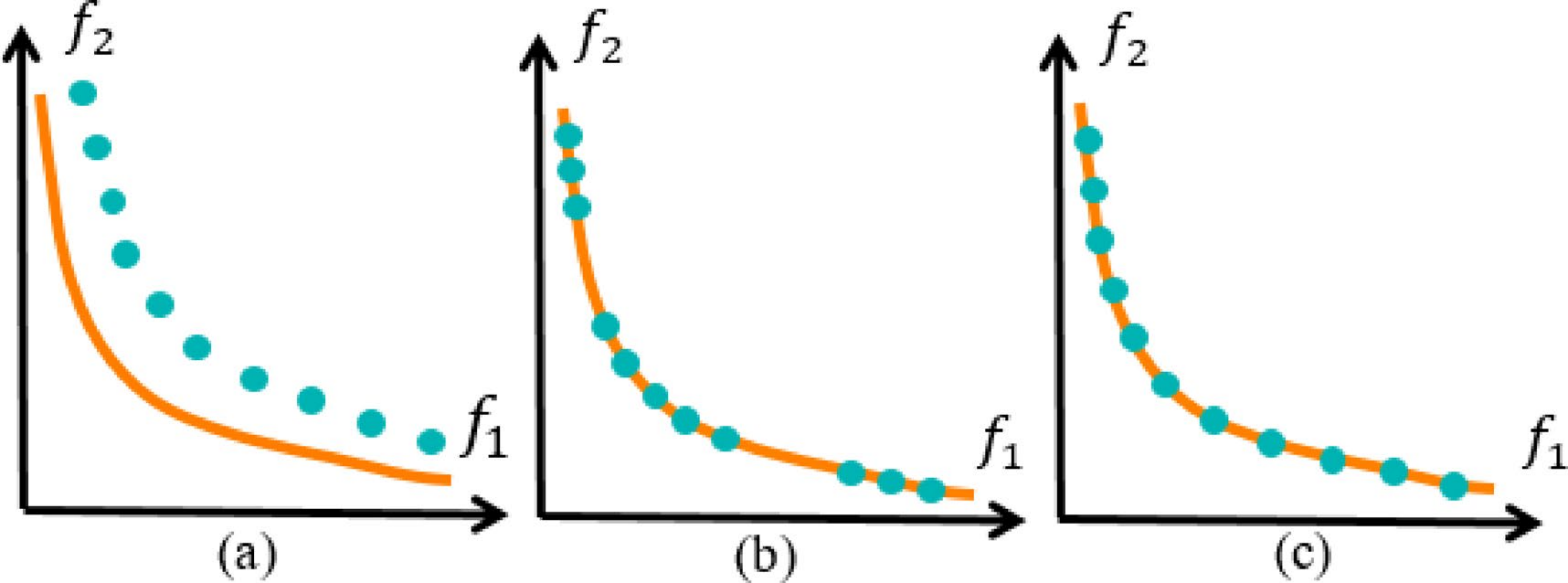
Key challenges in MOO: (**a**) Convergence; (**b**) Diversity; (**c**) True PF.

In sum, two essential objectives must be addressed when solving MOPs^27^: (a) Convergence – the ability to find ND solutions that are as close as possible to the true PF; (b) Diversity – the ability to maintain a well-distributed set of solutions across the front (as illustrated in Fig. 2).

Despite the significant progress achieved by many multi-objective metaheuristics such as NSGA-II, MOEA/D, and their numerous variants, existing approaches still face limitations when applied to complex real-world problems. Many algorithms tend to converge prematurely to local regions of the Pareto front, leading to a loss of diversity. Others require extensive parameter tuning, which reduces their robustness and practicality. Furthermore, the scalability of most existing MO algorithms remains a challenge when dealing with high-dimensional objectives or large-scale real-world problems. These limitations highlight the need for new algorithms that can balance convergence and diversity more effectively while maintaining robustness across different problem domains. Motivated by these gaps, we propose the Multi-Objective Starfish Optimization Algorithm (MOSFOA), which extends the strong single-objective performance of SFOA into the multi-objective domain through Pareto-based elitist sorting and crowding distance mechanisms.

### Related works

MOO seeks to approximate the PO front with a diverse set of ND solutions in a single execution^28^. Thanks to its numerous advantages, such as the ability to avoid local optima and not requiring gradient information^29^, MOO has become a crucial tool for solving complex, real-world problems in both research and industry. Over the past three decades, several stochastic MOO algorithms have been developed and refined to improve performance in terms of convergence and diversity. Notable among these influential algorithms are NSGA-II^17^, SPEA-II^18^, MOEA/D^19^, and MOPSO^16^.

NSGA-II, introduced by Deb et al. in 2002, is a well-known MOO algorithm that combines ND sorting with a crowding distance mechanism. It starts by producing an initial population and then applies selection, crossover, and mutation operations to create offspring. The parent and offspring populations are merged and sorted according to ND levels. The next generation is then formed by choosing individuals based on Pareto dominance and crowding distance, ensuring both convergence and diversity. This process iterates until the specified termination criteria are satisfied.

The notable success of NSGA-II has inspired the development of various methods that integrate ND sorting mechanisms, including: Multi-Objective Reptile Search Algorithm (MORSA)^30^, Multi-objective Mantis Search Algorithm (MOMSA), Non-Dominated Sorting Chicken Swarm Optimization (NSCSO)^31^, Multi-Objective RIME (MORIME)^32^, Multi-Objective Slime Mould Algorithm (MOSMA)^33^, Non-Dominated Sorting Advanced Butterfly Optimization Algorithm (NDSABOA)^34^, and Multi-Objective Artificial Hummingbird Algorithm (MOAHA)^35^.

Another important evolutionary method is SPEA-II (Strength Pareto Evolutionary Algorithm-II)^18^, an improved version of SPEA^36^. SPEA-II utilizes a fine-grained fitness assignment scheme, a fixed-size external archive for maintaining ND solutions, and a clustering technique for archive truncation. These enhancements enable better preservation of diversity and convergence compared to its predecessor.

MOEA/D^19^ offers a different perspective by decomposing an MOP into a set of scalar subproblems. Each subproblem is optimized simultaneously using evolutionary operators, and the neighborhood structure among subproblems is defined based on the distance between their associated weight vectors. This approach enables MOEA/D to exploit problem structure more effectively and better balances convergence and diversity. Compared to other MOEAs, MOEA/D efficiently addresses challenges related to fitness evaluation and diversity maintenance without explicitly relying on Pareto dominance.

MOPSO^16^ extends the standard PSO approach to handle multiple objectives by incorporating concepts such as external archives and mutation. MOPSO retains a historical repository of non-dominated solutions to ensure elitism. To maintain diversity and avoid premature convergence to a suboptimal front, mutation is periodically applied. Additionally, a geographically distributed strategy is employed for leader selection, improving exploration of the search space.

Beyond the previously mentioned algorithms, a wide array of additional multi-objective evolutionary and swarm intelligence-based methods have been introduced in the literature. Prominent examples include the Multi-Objective Ant colony optimization algorithm based on an elitist selection strategy^37^, the Multi-Objective Grey Wolf Optimizer (MOGWO)^38^, the Multi-Objective Ant Lion Optimizer (MOALO)^39^, the Strength Pareto Evolutionary Algorithm based on Reference Directions (SPEA/R)^40^, and Multi-Objective Equilibrium optimizer (MOEO)^41^. Other notable contributions include the Diversity-indicator based multi-objective evolutionary algorithm (DI-MOEA‏)^42^, the multi-objective seagull optimization algorithm (MOSOA)^43^, the Multi-Objective Boxing Match Algorithm (MOBMA)^44^, the Multi-Objective Material Generation algorithm (MOMGA)^45^, Multi-Objective Marine Predator Algorithm (MOMPA)^46^, the multi-objective Salp Swarm Algorithm (MSSA)^47^, and the Multi-Objective Quantum Genetic Algorithm (MOQGA)^48^, among others. For a more comprehensive understanding, readers are encouraged to consult recent review papers on multi-objective optimization techniques^49^. According to the No-Free-Lunch (NFL) theorem, no single algorithm performs best across all MOPs. This motivates the continuous development of new algorithms that either address previously unsolved problems or improve the performance of existing methods.

Despite the extensive progress in developing MOEAs, several important gaps remain unaddressed in the literature, which motivates our proposed MOSFOA. These gaps can be summarized as follows:

- In MOO, existing algorithms are not easily able to balance between convergence and diversity, especially with increasing numbers of objectives. This leads to higher computational complexity and the inability to approximate the true Pareto front properly.
- Most of the recent state-of-the-art MOEAs have not been extensively tested on large-scale benchmark sets or realistic real-world problems. Under limited function evaluations, they typically do not provide convergence or achieve an adequate distribution of non-dominated solutions.
- While some algorithms are robustly performing for single-objective optimization, their multi-objective variants typically do not provide such robustness and scalability. By generalizing a well-established single-objective framework into a multi-objective version, the gap might be closed.
- According to the No-Free-Lunch (NFL) theorem^50^, there is no optimization algorithm which performs best for all types of problems, and that justifies the development of novel algorithms which are efficient and optimal for diverse problem domains.
- While the SFOA has been effectively used in single-objective problems, there is no reported extension in the multi-objective direction in the existing literature. This leaves a clear gap and the opportunity to introduce the multi-objective version (MOSFOA) for the resolution of real-world optimization engineering and power system issues.

## Formulation of the OPF problem

Multi-objective OPF (MOOPF) problem aims to simultaneously minimize multiple objective functions while adhering to both equality and inequality constraints. Due to the inherent conflict among objectives, the solution is represented by a PF, illustrating the best possible trade-offs among competing goals. Mathematically, the MOOPF problem is formulated as follows^51^:

2 $$\:Min:\:F\left(x,u\right)=\:\left[{f}_{1}\left(x,u\right),{f}_{2}\left(x,u\right),\dots\:{f}_{N}\left(x,u\right)\right]$$

Subject to:

3 $$\:g\left(x,u\:\right)=0$$

4 $$\:h\left(x,u\right)\le\:0$$

Here, $\:f\left(x,u\right)$ denotes the objective function specific to the OPF problem, whereas $\:F\left(x,u\right)$ denotes the set of multiple objectives that are simultaneously optimized in the MOO framework. The term $\:g\left(x,u\:\right)$ represents the set of equality constraints, and $\:h\left(x,u\right)$ corresponds to the inequality constraints. In this formulation, $\:u$ represents the set of control variables, which includes:

1. Generator bus voltages.
2. Active power outputs at PV buses (excluding the slack bus).
3. Transformer tap settings.
4. Shunt VAR compensation devices.

The control vector$\:\:u$ can thus be formulated as:

5 $$\:u=\left[{P}_{{G}_{2}}\dots\:{P}_{{G}_{NG}},{V}_{{G}_{1}}\dots\:{V}_{{G}_{NG}},{Q}_{{C}_{1}}\dots\:{Q}_{{C}_{NC}},{T}_{1}\dots\:{T}_{NT}\right]$$

Meanwhile, x denotes the state variables, encompassing:

1. The active power output at the slack bus ($\:{P}_{{G}_{1}}$).
2. Load bus voltages.
3. Generator reactive power outputs.
4. Transmission line loadings (or power flows).

Consequently, the state vector x can be expressed as:

6 $$\:x=\left[{P}_{{G}_{1}},{V}_{{L}_{1}}\dots\:{V}_{{L}_{NL}},{Q}_{{G}_{1}}\dots\:{Q}_{{G}_{NG}},{S}_{{l}_{1}}\dots\:{S}_{{l}_{NTL}}\right]$$

### Objectives constraints

#### Equality constraint

In the OPF problem, the equality constraints are represented by the power balance equations for both active and reactive power, as shown in Eqs. (7) and (8).

7 $$\:{P}_{Gi}-{P}_{Di}-{V}_{i}\sum\:_{j=1}^{NB}\:{V}_{j}\left({G}_{ij}cos{\theta\:}_{ij}+{B}_{ij}sin{\theta\:}_{ij}\right)=0$$

8 $$\:{Q}_{Gi}-{Q}_{Di}-{V}_{i}\sum\:_{j=1}^{NB}\:{V}_{j}\left({G}_{ij}sin{\theta\:}_{ij}-{B}_{ij}cos{\theta\:}_{ij}\right)=0$$

Here, $\:NB$ denotes the total number of buses in the network, while $\:{P}_{D}$ and $\:{Q}_{D}\:$represent the active and reactive power demands at each bus, respectively. Additionally, $\:{G}_{ij}$ and $\:{B}_{ij}$ signify the conductance and susceptance values between buses$\:\:i\:$and $\:j$^52^.

#### Inequality constraints

In addition to meeting the power balance equations, the OPF problem must also comply with a series of operational limits known as inequality constraints. These constraints ensure that the system operates within safe and reliable boundaries. They are defined as follows^53^:

Generators limitations:

9 $$\:{V}_{Gi}^{min}\le\:{V}_{Gi}\le\:{V}_{Gi}^{max},i=1,\dots\:.,NG$$

10 $$\:{P}_{Gi}^{min}\le\:{P}_{Gi}\le\:{P}_{Gi}^{max},i=1,\dots\:.,NG$$

11 $$\:{Q}_{Gi}^{min}\le\:{Q}_{Gi}\le\:{Q}_{Gi}^{max},i=1,\dots\:.,NG$$

Transformer limitations:

12 $$\:{T}_{i}^{min}\le\:{T}_{i}\le\:{T}_{i}^{max},i=1,\dots\:.,NT$$

Shunt compensator limitations:

13 $$\:{Q}_{Ci}^{min}\le\:{Q}_{Ci}\le\:{Q}_{Ci}^{max}i=\mathrm{1,2},\dots\:,NC$$

Security limitations:

14 $$\:{V}_{Li}^{min}\le\:{V}_{Li}\le\:{V}_{Li}^{max},i=1,\dots\:,{N}_{PQ}$$

15 $$\:{S}_{li}\le\:{S}_{li}^{max},i=1,\dots\:,NL$$

### Objective functions

#### Fuel cost (FC)

One of the fundamental objectives in the OPF problem is to minimize fuel or generation costs. Generally, the relationship between generation cost (in $/h) and output power (in MW) is represented as a quadratic function, which can be expressed as follows^54^:

16 $$\:{f}_{FC}=\sum\:_{i=1}^{NG}\:\left({a}_{i}+{b}_{i}{P}_{Gi}+{c}_{i}{P}_{Gi}^{2}\right)$$

where $\:{a}_{i}$​, $\:{b}_{i}$​, and $\:{c}_{i}$ are the fuel cost coefficients associated with the *i-th* generator, and $\:{P}_{Gi}$​ represents its active power output.

#### Emission (EM)

Burning fossil fuels to generate electricity results in the release of hazardous gases, including sulfur oxides (SOₓ), nitrogen oxides (NOₓ), and carbon oxides (COₓ). With increasing environmental concerns, it has become crucial for power plants to reduce these emissions while ensuring consistent and reliable energy generation. The correlation between emission levels and the amount of real power produced is generally modeled using a quadratic function, as shown in^52^:

17 $$\:{f}_{EM}=\sum\:_{i=1}^{NG}\:\left({\alpha\:}_{i}+{\beta\:}_{i}{P}_{Gi}+{\gamma\:}_{i}{P}_{Gi}^{2}+{\delta\:}_{i}\mathrm{e}\mathrm{x}\mathrm{p}\left({\epsilon\:}_{i}{P}_{Gi}\right)\right)$$

where:

- $\:{\alpha\:}_{i}$​, $\:{\beta\:}_{i}$​, $\:{\gamma\:}_{i}$, $\:{\delta\:}_{i}$, and $\:{\epsilon\:}_{i}$​ are the emission coefficients of the *i-th* generator.
- $\:{P}_{Gi}$​ is the active power generated by the *i-th* unit.
- $\:NG$ represents the total number of generators.

#### Active power loss (PL)

In electrical transmission lines, inherent resistance and conductance with fixed parameters cause active power losses during power transfer across the network. These losses are unavoidable and impact the overall efficiency of the system. The total active power loss in the grid can be mathematically represented as^51^:

18 $$\:{f}_{PL}=\sum\:_{i=1}^{Nl}\:\sum\:_{j\ne\:i}^{Nl}\:{G}_{ij}\left[{V}_{i}^{2}+{V}_{j}^{2}-2{V}_{i}{V}_{j}cos\left({\theta\:}_{i}-{\theta\:}_{j}\right)\right]$$

where:

- $\:Nl$ is the total number of transmission lines,
- $\:{V}_{i}$ and $\:{V}_{j}$​ are the voltage magnitudes at buses $\:i$ and $\:j$.
- $\:{\theta\:}_{i}$​ and $\:{\theta\:}_{j}$ are the voltage phase angles at buses $\:i$ and $\:j$.
- $\:{G}_{ij}$​ represents the conductance between bus $\:i$ and bus $\:j$.

#### Voltage deviation (VD)

During the operation of power systems, voltage limit violations at load buses are often encountered, particularly under contingency conditions, making the situation more critical. To address this issue, the OPF problem includes an objective to minimize the total voltage deviation (TVD) at load buses. This approach aims to reduce the absolute difference between actual bus voltages and their desired reference values, thereby improving the overall voltage profile and enhancing the security and reliability of system operation. The voltage deviation minimization can be mathematically expressed as^55^:

19 $$\:{f}_{VD}=\sum\:_{i=1}^{NPQ}\:\left|\left({V}_{i}-{V}_{ref}\right)\right|$$

where $\:{V}_{ref}\:$denotes the reference voltage magnitude at each PQ bus, typically set to 1.0 p.u.

## Multi-objective SFOA (MOSFOA)

This section outlines the core mechanisms of the SFOA. Following this, we introduce an extension of the algorithm to address MOPs.

### SFOA algorithm

SFOA^11^ is a novel stochastic search optimization method inspired by the exploring, preying, and regeneration behaviors of starfish. Starfish, which are marine invertebrates with more than 2,000 known species globally, typically exhibit a star-shaped body with five arms radiating from a central disc.

Like most modern metaheuristic methods, SFOA progresses through two main stages: exploration and exploitation. In the exploration phase, the algorithm mimics the starfish’s searching behavior, while the exploitation phase draws on their preying and regeneration actions to enhance solution quality. These natural behaviors are captured through mathematical modeling, forming the foundation of SFOA’s approach to addressing global optimization tasks. The algorithm’s mathematical formulation is detailed below.

#### Initialization

SFOA initializes by producing an initial population of $\:N$ starfish positions within the search space, mathematically defined by Eq. (20):

20 $$\:{X}_{ij}=l{b}_{i}\mathrm{+}r\times\:\left(u{b}_{i}-l{b}_{i}\right){\hspace{0.05em}\hspace{0.05em}\hspace{0.05em}}\left\{\begin{array}{c}i=\mathrm{1,2},...,\:N\\\:j=\mathrm{1,2},...,\:D\end{array}\right.$$

where $\:{X}_{ij}$​ refers the position of the $\:ith$ starfish in the $\:jth$ dimension, $\:r$ is a random number generated from $\:\left(\mathrm{0,1}\right).$ $\:{ub}_{i}$ and $\:l{b}_{i}\:$denote the upper and lower bounds of the design variables in the $\:jth$ dimension, respectively. Although the above equation represents a standard uniform random initialization, it is particularly effective in high-dimensional search spaces because it ensures that each decision variable is independently sampled across its feasible range. This provides an unbiased coverage of the solution domain and maintains sufficient diversity in the initial population, which is essential for balancing exploration and exploitation in the subsequent evolutionary process.

#### Exploration phase

To model the exploratory behavior of starfish, the SFOA algorithm incorporates an exploration phase that mimics the searching ability of its five arms, each equipped with an eye. This phase introduces a novel search pattern: a five-dimensional search strategy for high-dimensional problems ($\:D>5$) and a unidimensional approach for lower-dimensional cases ($\:D\le\:5$). The threshold of five dimensions is inspired by the natural structure of starfish, which typically possess five arms acting as independent search directions. From a computational perspective, using five parallel search trajectories enhances diversity in larger search spaces ($\:D>5$), while for smaller search spaces ($\:D\le\:5$), a unidimensional exploration is sufficient and computationally efficient. Thus, the distinction between the two strategies is biologically motivated and further justified with computational and empirical evidence, as rigorously analyzed in the foundational study^11^.

For problems with $\:D>5$, the vast search space necessitates coordinated movement of all five arms, guided by the best-known positions of search agents. Accordingly, a mathematical model is developed to simulate this phase.

21 $$\:\left\{\begin{array}{c}\begin{array}{cc}{Y}_{i,p}^{t}={X}_{i,p}^{t}+{a}_{1\times\:}\left({X}_{best,p}^{t}-{X}_{i,p}^{t}\right)\times\:cos\:\theta\:,&\:r\le\:0.5\end{array}\\\:\begin{array}{cc}{Y}_{i,p}^{t}={X}_{i,p}^{t}-\:{a}_{1}\times\:\left({X}_{best,p}^{t}-{X}_{i,p}^{t}\right)\times\:sin\:\theta\:,&\:r>0.5\end{array}\end{array}\right.$$

where $\:{Y}_{i,p}^{t}$ and $\:{X}_{i,p}^{t}$​ represent the updated and current positions of the $\:ith$ starfish at iteration $\:t$, respectively. $\:{X}_{best,p}^{t}$ ​ denotes the $\:pth$ dimension of the current best position. The value of $\:p$ corresponds to five randomly chosen dimensions from the total $\:D$ dimensions, while $\:r$ is a random number within the range $\:\left(\mathrm{0,1}\right)$. The parameters $\:{a}_{1}$​ and $\:\:\theta\:$ are computed as follows:

22 $$\:{a}_{1}=\left(2\times\:r-1\right)\times\:\pi\:$$

23 $$\:\theta\:=\frac{\pi\:\times\:t}{2\times\:\:{G}_{max}}$$

where$\:\:{G}_{max}$​ is the maximum number of generations, $\:t$ is the current generation, sine and cosine functions model the starfish arms’ movement, allowing them to twist left or right with equal probability while searching for food. During the exploration phase, the parameter $\:{a}_{1}$ is randomly produced for each candidate solution in every iteration. Meanwhile, the angle $\:\theta\:$ varies throughout the iterations within the range $\:\left[0,\pi\:/2\right]$, affecting how the distance between the best and current positions influences the selected dimensions. For further explanation on the effect of these parameters on convergence, we refer the reader to the original SFOA study^11^.

For optimization problems with $\:D>5$, a five-dimensional search pattern is employed. This strategy updates only five randomly selected dimensions per iteration, thereby improving search efficiency relative to updating the entire decision vector. If an updated position violates the design variable bounds, the corresponding starfish arm maintains its previous position instead of moving beyond the permissible limits. The mathematical formulation of this mechanism is presented below:

24 $$\:{X}_{i,p}^{t+1}=\left\{\begin{array}{c}\begin{array}{cc}{Y}_{i,p}^{t},&\:l{b}_{p}\le\:{Y}_{i,p}^{t}\le\:u{b}_{p}\end{array}\\\:\begin{array}{cc}{X}_{i,p}^{t},&\:Else\end{array}\end{array}\right.$$

where $\:p$ represents the updated dimension. This boundary-handling strategy guarantees feasibility and prevents invalid updates. However, when multiple dimensions simultaneously exceed the limits, the mechanism may reduce exploration capability since several arms remain fixed at their previous positions. Despite this potential drawback, it helps maintain stability during the search process.

For optimization problems with $\:D\le\:5$, the exploration phase utilizes a one-dimensional search strategy. In this approach, a single arm of the starfish advances toward the food source by incorporating positional information from other individuals in the population. The position update is computed using the following formulation:

25 $$\:{Y}_{i,p}^{t}={E}_{t}\times\:{X}_{i,p}^{t}+{A}_{1}\times\:\left({X}_{{k}_{1},p}^{t}-{X}_{i,p}^{t}\right)+{A}_{2}\times\:\left({X}_{{k}_{2},p}^{t}-{X}_{i,p}^{t}\right)$$

where $\:{X}_{{\mathrm{k}}_{1},p}^{t}$​ and $\:{X}_{{\mathrm{k}}_{2},p}^{t}$​ represent the positions of two randomly chosen starfish in the $\:p$-th dimension at iteration t. $\:{A}_{1}$​ and $\:{A}_{2}$​ are random numbers within the range (-1,1), while $\:p$ denotes a randomly selected dimension from the total $\:D$ dimensions. The energy level of a starfish at iteration t, denoted by $\:{E}_{t}$​, is defined as follows:

26 $$\:{E}_{t}=\left(1-\frac{t}{{G}_{max}}\right)\times\:\:cos\:\theta\:$$

where $\:\theta\:$ is determined using Eq. (23). Similar to the previous update rule (Eq. (24)), if the new position of a starfish exceeds the boundary constraints, it remains in its previous position instead of adopting the updated position.

#### Exploitation phase

In this phase, SFOA integrates preying and regeneration mechanisms to boost the efficiency of the global search process. To achieve this, two specific position-updating strategies are employed. The preying behavior is modeled through a parallel bi-directional search approach, which utilizes information from both the best-known solution and the remaining starfish in the population. Initially, five distances are computed between the global best solution and other starfish. From these, two distances are randomly selected for each starfish. These selected distances are then used in the position update process via the parallel two-directional strategy. The calculation of these distances is given as follows:

27 $$\:{d}_{m}=\left({X}_{best}^{t}-{X}_{\:{m}_{p}}^{t}\right),\:\:\:\:\:\:m=1,\dots\:,5$$

Here, $\:{d}_{m}\:$denotes the set of five computed distances between the global best position and selected starfish, while $\:{m}_{p}$ refers to five randomly chosen individuals from the population. Using this information, the position of each starfish during the preying phase is updated according to the following rule:

28 $$\:{Y}_{i}^{t}={X}_{i}^{t}{+{r}_{1}\times\:d}_{\:{m}_{1}}{+{r}_{2}\times\:d}_{{m}_{1}}$$

where$\:{\:r}_{1}$ and $\:{r}_{2}\:$are randomly generated values within the interval (0,1), while $\:\:{d}_{{m}_{1}}\:$and $\:\:{d}_{{m}_{2}}\:$​ are randomly chosen from $\:{d}_{m}.$.

Due to their slow movement, starfish are vulnerable to predators and may detach an arm to escape. In SFOA, the regeneration stage applies only to the last individual in the population ($\:i=N$). Since regeneration takes months in nature, the movement speed is slow. The position update in this phase is modeled as:

29 $$\:{Y}_{i}^{t}={X}_{i}^{t}\times\:{e}^{-\left(\frac{t}{{G}_{max}}\times\:N\right)}$$

The exponential decay term in Eq. (29) dynamically controls the exploitation intensity. At the early stages of the search, the term has a relatively large value, allowing broader movements and a higher chance of escaping local optima. As the number of iterations increases, the term decreases exponentially, thereby reducing the step size and directing the search more intensively toward promising regions. This adaptive adjustment enhances convergence speed while maintaining solution quality.

Finally, if the position obtained from Eq. (28) or Eq. (29) falls outside the permissible bounds of the design variables, it is adjusted as follows:

30 $$\:{X}_{i}^{t+1}=\left\{\begin{array}{c}\begin{array}{cc}{Y}_{i}^{t},&\:lb\le\:{Y}_{i}^{t}\le\:ub\end{array}\\\:\begin{array}{cc}lb,\:&\:ub<lb\end{array}\\\:\begin{array}{cc}ub,\:&\:{Y}_{i}^{t}\end{array}>ub\end{array}\right.$$

In SFOA, boundary handling is explicitly managed through Eqs. (24) and (30). During the exploration phase, as described in Eq. (24), if an updated position in any dimension exceeds the defined bounds, the corresponding arm of the starfish retains its previous position to ensure feasibility. Similarly, in the exploitation phase (Eq. (30)), any position that violates the permissible range is adjusted to remain within the search boundaries. This mechanism ensures that all candidate solutions stay feasible across the entire optimization process, maintaining stability and promoting controlled convergence even when dealing with highly multi-modal functions.

To ensure a balance between exploration and exploitation, each phase in SFOA is selected with equal likelihood by comparing a randomly generated number in the range (0,1) to the algorithmic control parameter $\:{G}_{p}$. A sensitivity analysis of $\:{G}_{p}$ was conducted in the original SFOA study. The analysis showed that setting $\:{G}_{p}=0.5$ provides the best balance between exploration and exploitation, ensuring stable convergence.

The biological inspiration of SFOA is drawn from three key starfish behaviors:

- Exploration corresponds to the algorithm’s hybrid search strategy, which integrates five-dimensional and one-dimensional movement patterns to expand the search space and avoid premature convergence.
- Predation is translated into a two-directional search mechanism that intensifies exploitation around promising areas, mimicking how starfish pursue and capture prey.
- Regeneration is modeled as a re-initialization and adaptive movement strategy, allowing the algorithm to recover lost diversity and redirect the search toward unexplored regions.

These mechanisms collectively enhance optimization efficiency by promoting exploration diversity, accelerating convergence, and preserving solution quality. Moreover, they provide computational advantages by maintaining an adaptive balance between global exploration and local exploitation, thereby improving the algorithm’s robustness and search capability across complex optimization landscapes.

Finally, for clarity, the starfish-inspired mechanisms described above—arm updates, energy levels, and parallel bi-directional search—can be interpreted in terms of standard optimization concepts.

*Starfish arm updates* correspond to position updates of candidate solutions in the search space, similar to movements in other population-based algorithms.

*Energy level* reflects the quality or fitness of a solution, guiding its exploration and exploitation behavior.

*Parallel bi-directional search* represents simultaneous exploration and exploitation, analogous to using both global and local search operators in conventional optimization.

The algorithm’s detailed procedure is outlined in Algorithm 1.

**Algorithm 1:**
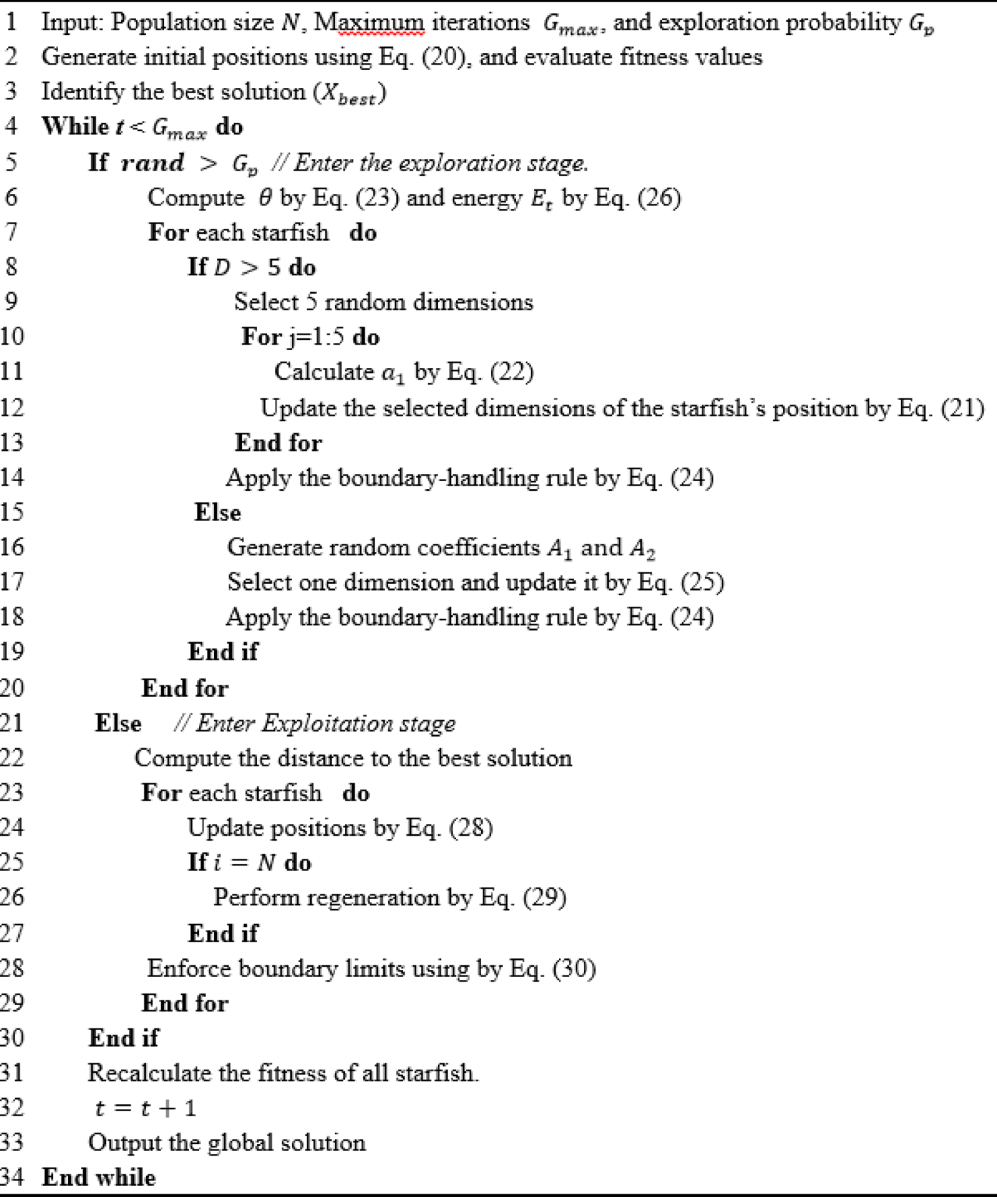
SFOA algorithm.

### Proposed MOSFOA algorithm

In this study, a novel multi-objective starfish optimization algorithm (MOSFOA) is proposed, incorporating Non-Dominated Sorting (NDS) and Crowding Distance (CD) mechanisms to address MOO problems. These mechanisms, collectively referred to as elitist mechanisms, aim to enhance convergence speed toward the Pareto front while maintaining solution diversity across generations^56^.

The NDS technique manages Pareto dominance by ranking solutions according to their dominance levels and preserving the best non-dominated solutions. This ensures that higher-quality solutions are retained throughout the evolutionary process, thereby accelerating convergence to the global PF. The CD method measures the relative spacing between solutions in the same dominance front, prioritizing those with greater CD to prevent clustering and promote exploration of less crowded regions in the objective space. By integrating these elitist mechanisms, MOSFOA balances exploitation and exploration, reducing the risk of premature convergence while maintaining a diverse PO set.

**Fig. 3:**
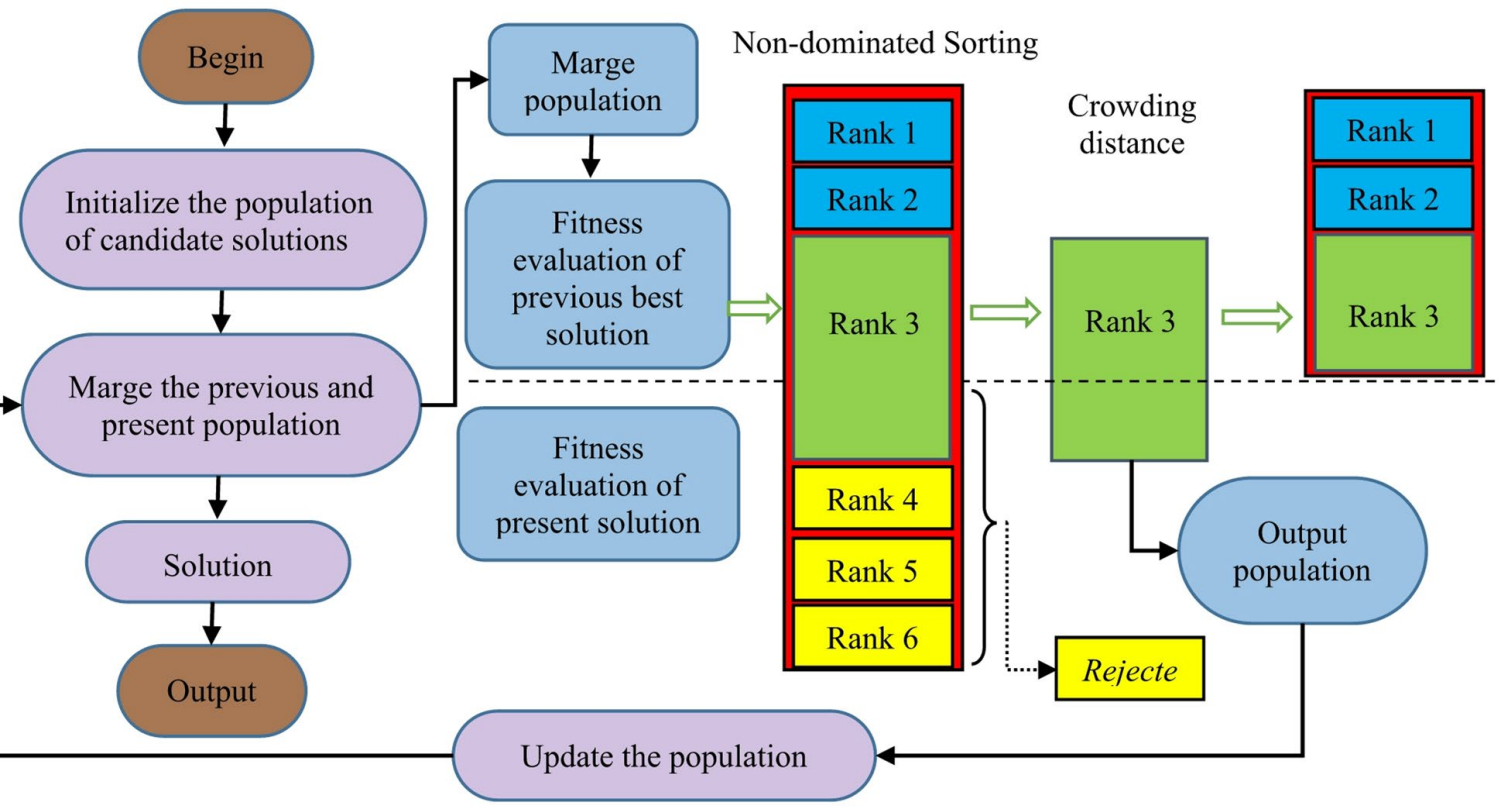
The procedure of non-dominated sorting approach.

The proposed MOSFOA follows a structured approach, illustrated in Fig. 3, and is composed of the following key steps:

- **Step 1**: Identify ND solutions.
- **Step 2**: Apply the NDS method to categorize solutions into dominance fronts.
- **Step 3**: Calculate the Non-Dominated Ranking (NDR) for all ND solutions.

**Fig. 4:**
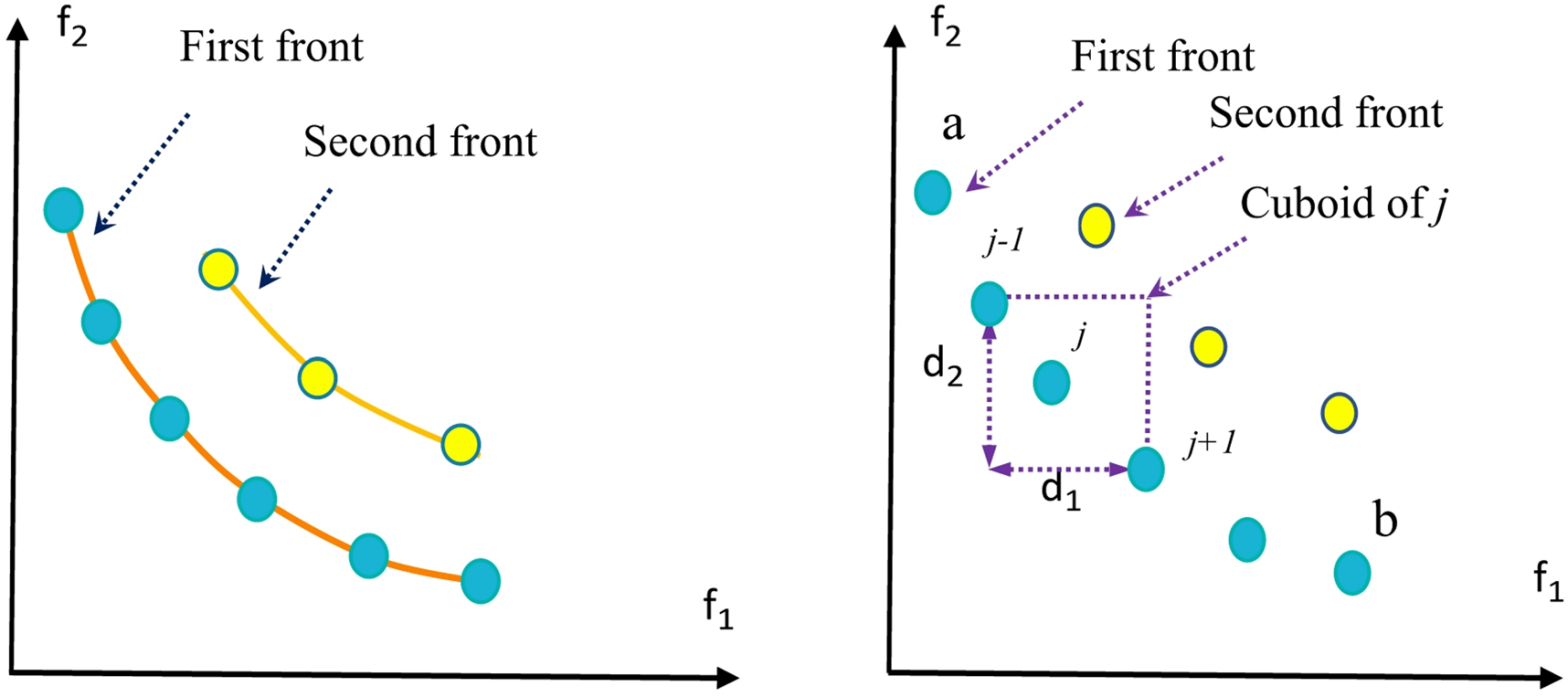
Objective space: (**a**) NDR method; (**b**) CD method.

The NDR process, illustrated in Fig. 4 (a), ranks solutions by dominance level. Solutions in the first front (depicted in light color) are non-dominated by any other solutions, while those in the second front (shown in yellow color) are dominated by at least one solution from the first front. Higher priority is assigned to solutions in the first front. When solutions share the same front, the CD is used to prioritize those with greater spacing, as illustrated in Fig. 4 (b).

**Fig. 5:**
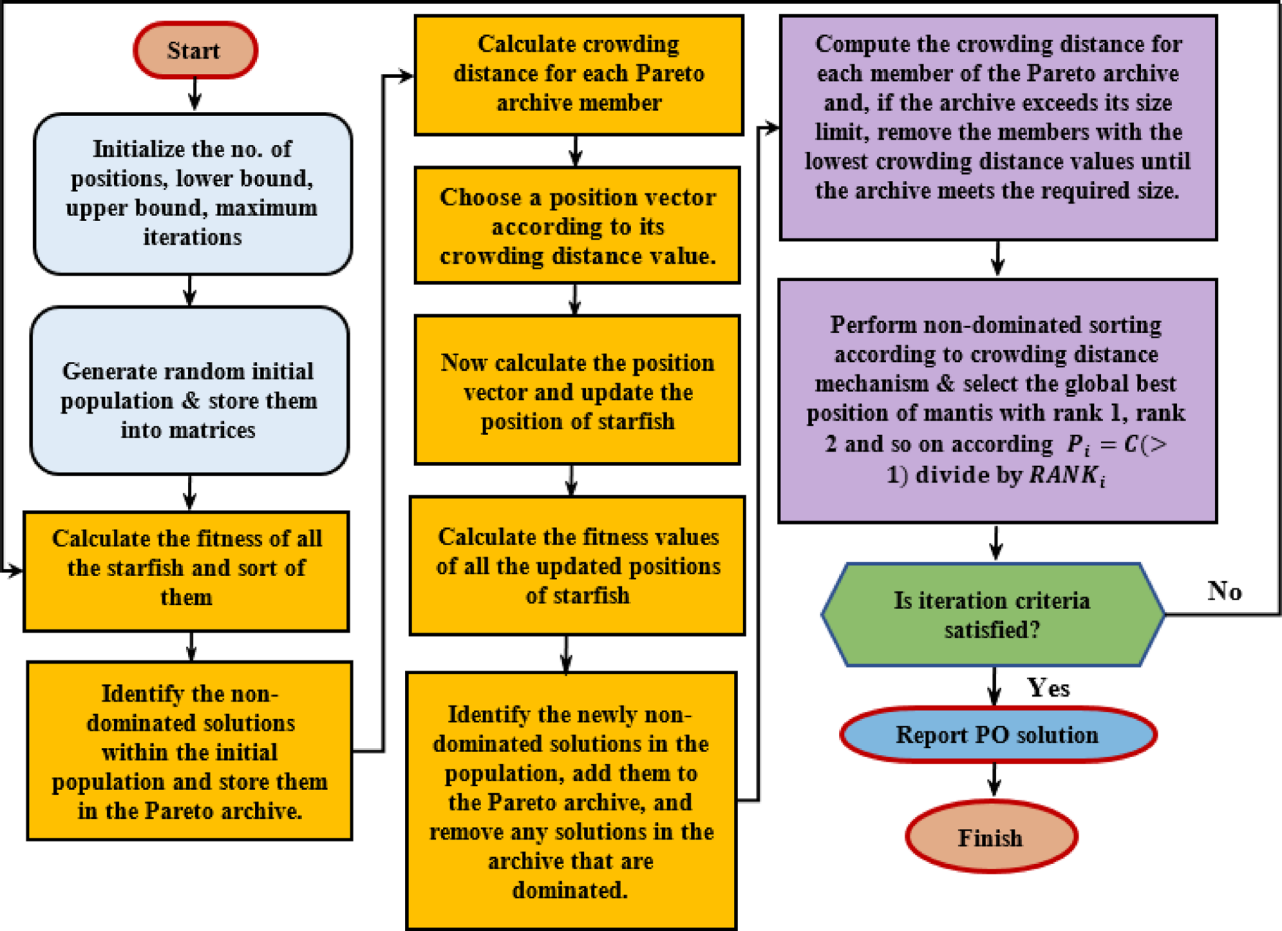
Flowchart of MOSFOA.

The formulation of the CD mechanism is presented as:

31 $$\:C{D}_{j}^{i}=\frac{{f}_{j}^{i+1}-{f}_{j}^{i-1}}{{f}_{j}^{max}-{f}_{j}^{min}}$$

where the values $\:{f}_{j}^{max}\:$and $\:{f}_{j}^{min}$ represent the maximum and minimum values of the $\:jth$ objective function, respectively. Figure 3 provides a diagrammatic representation of the NDS-based approach. Algorithm 2 outlines the pseudocode for MOSFOA, showing how elitist mechanisms are integrated at each generation to preserve high-quality solutions and maintain a diverse population. The flowchart of the introduced MOSFOA is depicted in Fig. 5.

By explicitly incorporating NDS and CD, MOSFOA accelerates convergence toward the global Pareto front while preserving high-quality solutions and maintaining diversity across generations, effectively addressing potential conflicts between objectives in MOO.

The overall MOSFOA process is summarized as follows:

1. *Initialization*: The necessary parameters are initialized, including the population size ($\:N$​), termination criteria, and the maximum number of generations or iterations ($\:{G}_{max}$).
2. *Generation of Initial Population*: An initial parent population ($\:{P}_{0}$​) is randomly produced within the feasible search space $\:S.\:$ Each individual in $\:{P}_{0}$ is evaluated based on the objective functions defined in the objective space vector *F.*
3. *Elitist Mechanisms*: Elitist mechanisms based on CD and NDS are applied to $\:{P}_{0}.\:$This helps in selecting individuals that are more likely to lead to optimal solutions.
4. *Population Update*: A new population ($\:{P}_{j}$​) is created and merged with $\:{P}_{0}$ to form a combined population $\:{R}_{i}$. This combined population is then sorted using elitist non-domination principles, which incorporate both CD and NDR.
5. *Selection of Top Solutions*: The best $\:N$ solutions from the merged population are chosen to form the new parent population for the next generation.
6. *Termination*: this process is repeated iteratively until the predefined termination criteria are Met

While MOSFOA, like NSGA-II, relies on nondominated sorting as the core ranking mechanism, its novelty lies in the way starfish-inspired operators are integrated to improve convergence and diversity. Unlike MOEA/D, which decomposes the problem, MOSFOA enriches the dominance-based framework with:

- Biologically motivated exploration (five-dimensional and one-dimensional arm-based search).
- Predation-inspired exploitation, enhancing local intensification around promising solutions.
- Regeneration mechanism, which adaptively restores diversity and prevents stagnation.

This integration enables MOSFOA to go beyond conventional dominance-based algorithms by maintaining a better balance between exploration and exploitation, leading to superior Pareto fronts in both benchmark functions and real-world OPF problems.

**Algorithm 2:**
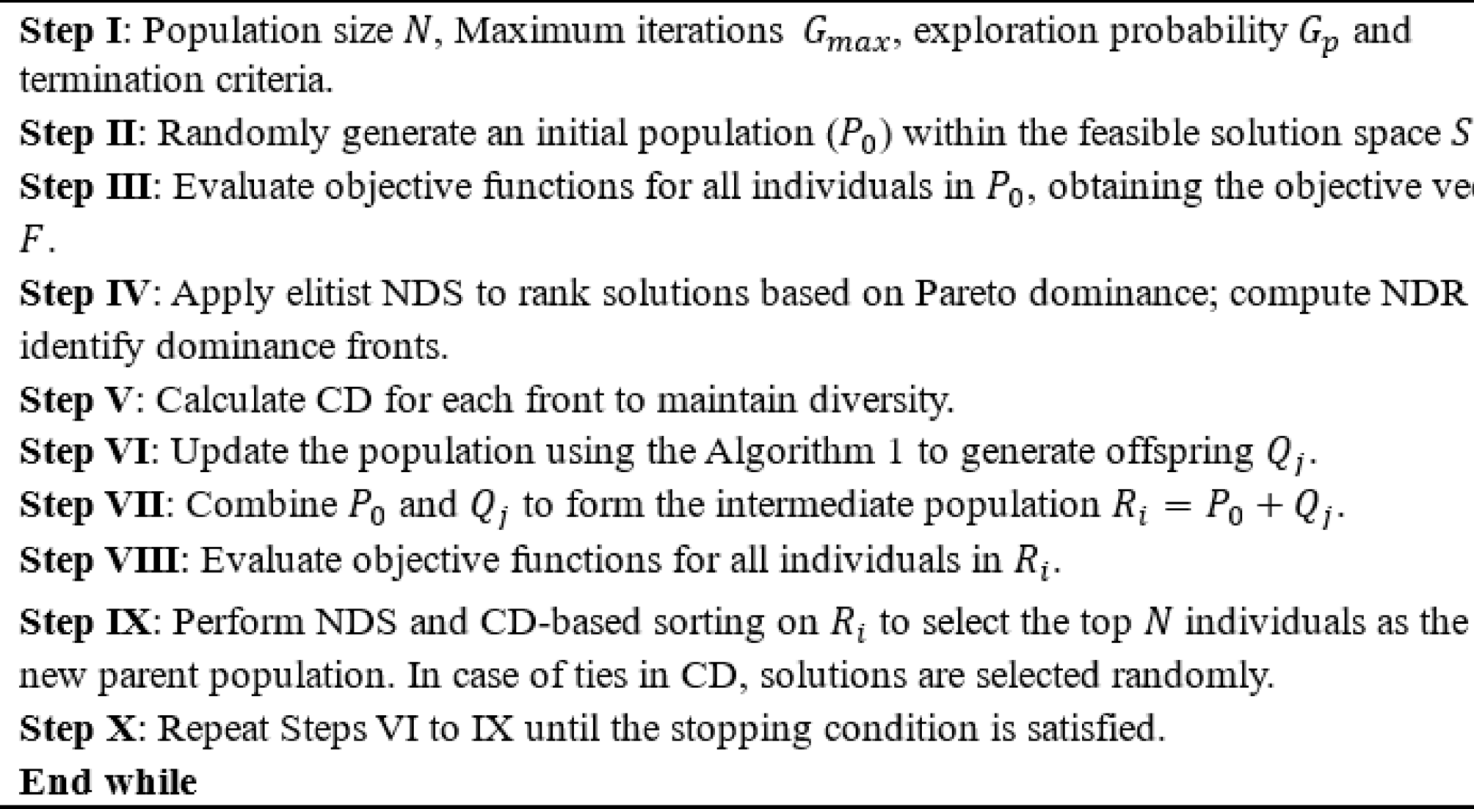
Pseudo-code of MOSFOA.

#### Computational complexity of MOSFOA

The computational complexity of MOSFOA is $\:O{\left(M\cdot\:N\right)}^{2}$, where $\:N$ is the population size and $\:M$ is the number of objective functions. The per-iteration computational time complexity is as follows: for the first iteration, it is

$$\:O(D\cdot\:N+Cost({f}_{obj})\cdot\:N),$$

and for subsequent iterations, which include NDS and CD calculations, it becomes

$$\:O(D\cdot\:N+Cost({f}_{obj})\cdot\:N+(NDS+CD)\cdot\:D).$$

The overall computational time complexity for Maxit iterations is

$$\:O({G}_{max}\cdot\:D\cdot\:N+{G}_{max}\cdot\:Cost({f}_{obj})\cdot\:N+{G}_{max}\cdot\:(NDS+CD)\cdot\:D+{G}_{max}\cdot\:(NDS+CD)\cdot\:Cost({f}_{obj}\left)\right)$$

where $\:Cost\left({f}_{obj}\right)$ denotes the cost of evaluating the objective function, $\:D$ is the number of variables in the objective function, and $\:{G}_{max}\:$is the maximum number of iterations.

## Results and discussion

This section outlines the experimental setup utilized to assess and compare the performance of various MOO methods. Subsequently, the developed MOSFOA approach is evaluated on a set of MOPs to assess its efficiency.

### Experimental setup

To evaluate the performance of the developed method, a diverse set of benchmark test functions is utilized. These functions are categorized into three main groups:

- Unconstrained multi-Objective test problems: ZDT1-4, ZDT6, DTLZ1, DTLZ2, DTLZ4, DTLZ5, and DTLZ7.
- Constrained and engineering design multi-objective problems: BNH, SRN, OSY, TNK, Car, Disk Brake, 4-Bar Truss, CONSRT, and Welded Beam.
- IEEE 30-Bus power system case studies: The IEEE 30-bus power system is applied to four case studies using bi-objective and tri-objective OPF problems.

The mathematical formulations for these problems are provided in the references listed in Tables 1 and 2 (fifth column).

The selection of these benchmark problems is based on the following considerations:

- The test problems exhibit a wide range of PF shapes, including concave, convex, linear, and discontinuous (separated) fronts, offering a robust means of evaluating the algorithm’s adaptability.
- Real-world constrained optimization problems are inherently more complex and provide a more challenging test environment for optimization algorithms.

To assess the performance of the introduced MOSFOA against other methods, its findings are compared with those of ten robust MOO algorithms, divided into two groups:

- Mathematical benchmark problems: MOSFOA is compared with five methods: MOPSO^16^, MOGWO^38^, MOMVO^57^, MOEA/D^19^, and MOEDO^58^.
- IEEE 30-bus power system problems: MOSFOA is compared with five algorithms: MOALO^39^, MOAVOA^21^, MOGOA^59^, SPEA-II^18^, and MODA^60^.

The initial parameter configurations for all competitive methods are summarized in

Table 3. To improve clarity and reproducibility, Table 4 presents the parameters of SFOA and MOSFOA. Each algorithm is executed with a consistent population size of 100. The maximum number of generations is set to 300 for bi-objective problems and 500 for tri-objective problems. To ensure fairness and statistical reliability, each problem is solved in 11 independent runs for each method.

The experimentation and algorithms are conducted using MATLAB R2019a on a 64-bit Microsoft Windows 10 Pro system. The computations are performed on a machine equipped with an Intel Core i5 processor (3.20 GHz) and 8 GB of RAM.

**Table 1:** Description of unconstrained test problems used in the study.

Problem name	#Objectives	#Variables	PF shape / characteristics	Notes	Reference
ZDT1	2	30	Convex PF	Smooth and continuous	^61^
ZDT2	2	30	Non-convex PF	Concave regions	^61^
ZDT3	2	30	Disconnected PF	Five PF segments	^61^
ZDT4	2	10	Multimodal (~ 100 local PFs)	Very challenging	^61^
ZDT6	2	10	Non-convex PF	Biased search space	^61^
DTLZ1	3	5	Linear PF	Multimodal	^61^
DTLZ2	3	12	Spherical PF	Symmetric PF	^61^
DTLZ4	3	12	Spherical PF (biased)	Tests distribution	^61^
DTLZ5	3	12	Curved PF	Degenerated PF	^61^
DTLZ7	3	22	Disconnected PF	Multiple PF regions	^61^

**Table 2:** Description of constrained/engineering test problems used in the study.

Problem name	#Objectives	#Variables	#Constraints	Reference
BNH	2	2	2	^62^
SRN	2	2	2	^62^
OSY	2	6	6	^62^
TNK	2	2	2	^62^
CAR	3	7	10	^63^
DISK BRAKE	2	4	5	^17^
4-BAR TRUSS	2	4	-	^17^
CONSTR	2	2	2	^17^
WELDED BEAM	2	4	4	^20^

**Table 3:** Parameters setting of all methods.

Algorithm	Specific parameters	Value
MOPSO	Archive size, nGrid, α, β, γ, $\:\mu\:,$ $\:{w,\:C}_{1},\:{C}_{2}$	100, 30, 0.1, 4, 2, 0.1, 0.1, 1, 2
MOGWO	Archive size, nGrid, α, β, γ	100, 30, 0.1, 4, 2
MOMVO	$\:\mathrm{A}\mathrm{r}\mathrm{c}{\mathrm{h}}_{\mathrm{s}}\mathrm{i}\mathrm{z}\mathrm{e}$, $\:{WEP}_{max},\:{WEP}_{min}$	100, 1, 0.2
MOEA/D	Archive size, *CR*,$\:{\:T}_{n}$	100, 0.5, 15
MOEDO	-	-
MOALO	-	-
MOAVOA	Archive size, nGrid, α, β, γ	100, 30, 0.1, 4, 2
MOGOA	$\:\mathrm{c}\mathrm{m}\mathrm{i}\mathrm{n}$, $\:\mathrm{c}\mathrm{m}\mathrm{a}\mathrm{x}$, $\:\mathrm{A}\mathrm{r}\mathrm{c}{\mathrm{h}}_{\mathrm{s}}\mathrm{i}\mathrm{z}\mathrm{e}$	0.00004, 1,100
SPEA-II	Archive size, $\:{P}_{c},\:{\:P}_{m}$	100, 0.7, 0.2
MODA	-	-

**Table 4:** Parameters setting of SFOA and MOSFOA.

Algorithm	Specific parameters	Value
SFOA	Control parameter ($\:{G}_{p})$	0.5
	Population size (*N*)	100
	Maximum number of generations ($\:{G}_{max})$	(for single-objective OPF)
MOSFOA	Control parameter ($\:{G}_{p})$	0.5
	Population size (*N*)	100
	Maximum number of generations ($\:{G}_{max})$	300 (bi-objective), 500 (tri-objective)

### Performance metrics

Two widely used performance indicators are employed to evaluate the effectiveness of the proposed algorithm and its competitors: Inverted Generational Distance (IGD) and Hypervolume (HV)^64,65^. These metrics are widely recognized in multi-objective optimization as standard indicators for assessing both convergence and diversity^66–69^.

- Inverted generational distance (IGD):

The IGD metric^64^ is utilized to evaluate the diversity and convergence of the obtained PF toward the true PF. The IGD is defined as follows:

32 $$\:IGD\left({P}^{*},A\right)=\frac{1}{\left|{P}^{*}\right|}\sum\:_{i=1}^{\left|{P}^{*}\right|}{d}_{i}^{0}$$

where $\:{P}^{*}$ is set of true PO solutions (reference set), $\:\left|{P}^{*}\right|$ is the number of solutions in $\:{P}^{*}$, $\:{d}_{i}^{0}$ represents the Euclidean distance between the *ith* solution in the true PF and the nearest solution in the acquired approximation set $\:A$. Lower IGD values indicate better convergence and diversity.

- Hypervolume (HV):

The HV metric^65^ measures both convergence and diversity by calculating the volume of the objective space dominated by the obtained PF and bounded by a reference point. HV is a robust and widely accepted indicator in the evaluation of multi-modal and multi-objective evolutionary algorithms. It captures the overall distribution of solutions in the objective space. The mathematical formulation of HV is given as:

33 $$\:HV=Volume\:\left(\bigcup\:_{x\in\:A}\left[x,{r}^{*}\right]\right)$$

where $\:A$ is the obtained approximation set, $\:{r}^{\mathrm{*}}\:$is a user-defined reference point in the objective space, $\:\left[x,{r}^{\mathrm{*}}\right]$represents the hyper-rectangle formed between a solution $\:x$ and the reference point $\:{r}^{\mathrm{*}}$. A higher *HV* value signifies better spread and convergence of the solutions.

- KKT proximity metric (KKTPM):

Karush–Kuhn–Tucker (KKT) conditions constitute one of the fundamental tools in classical optimization theory^70^. They were originally established to verify the optimality of candidate solutions in single-objective constrained optimization problems. When these conditions are satisfied, a solution is considered stationary and potentially optimal. However, because the classical KKT framework is tailored to single-objective settings, it cannot be directly applied to MOO problems, where optimality is defined in relation to Pareto efficiency rather than a single scalar objective.

To address this limitation, Deb and Abouhawwash introduced the KKT Proximity Metric (KKTPM), an adaptation of the KKT concept that enables its use in multi-objective optimization^71^. Their metric is based on KKT conditions and employs an Achievement Scalarization Function (ASF) to handle multi-objective problems. KKTPM serves as a convergence indicator capable of quantifying how close a solution is to satisfying generalized KKT-like conditions associated with Pareto optimality. Unlike many performance metrics, KKTPM does not require prior knowledge of the true PF front and can also differentiate between feasible and infeasible solutions directly in the objective space. For additional technical details, readers may refer to the relevant literature^27,72,73^.

In general, KKTPM is formulated as an optimization problem as follows:

34 $$\begin{array}{*{20}c} {Minimize_{{\left( {\epsilon _{k} ,x_{{n + 1}} ,u} \right)}} ~~\epsilon _{k} + \mathop \sum \limits_{{j = 1}}^{J} \left( {u_{{M + j}} g_{j} \left( {x^{k} } \right)} \right)^{2} } \\ {~subject~to~\left\| {\nabla F\left( y \right) + \mathop \sum \limits_{{j = 1}}^{{M + J}} u_{j} \nabla G_{j} \left( y \right)} \right\|^{2} \le \epsilon _{k} } \\ {\mathop \sum \limits_{{j = 1}}^{{M + J}} u_{j} G_{j} \left( y \right) \ge - \epsilon _{k} } \\ {u_{j} \ge 0,j = 1,2, \ldots ,\left( {M + J} \right)} \\ {\left( {\frac{{f_{j} \left( x \right) - z_{j} }}{{w_{j}^{k} }}} \right) - x_{{n + 1}} \le 0,j = 1, \ldots ,M.} \\ \end{array}$$

where the constraints $\:{G}_{j}\left(\mathrm{y}\right)$ are given below:

35 $$\:\begin{array}{c}{G}_{j}\left(y\right)=\left(\frac{{f}_{j}\left(\mathrm{x}\right)-{z}_{j}}{{w}_{j}^{k}}\right)-{x}_{n+1}\le\:0,j=1,\dots\:,M,\\\:{G}_{M+j}\left(y\right)={g}_{j}\left(x\right)\le\:0,j=\mathrm{1,2},\dots\:,J.\end{array}$$

36 $$\:\mathrm{y}\:=\:(\mathrm{x};\:{x}_{n+1})$$

37 $$\:{w}_{i}=\frac{{f}_{i}\left({\mathrm{x}}^{k}\right)-{z}_{i}}{\sqrt{\sum\:_{m=1}^{M}\:{\left({f}_{m}\left({\mathrm{x}}^{k}\right)-{z}_{m}\right)}^{2}}}$$

where $\:w$ represents the weight vector, $\:M$ denotes the number of objective functions, and $\:J$ corresponds to the number of inequality constraints. The parameter u_i_ is the Lagrange multiplier associated with the *ith* inequality constraint. The term $\:{x}_{n+1}$ denotes a slack variable, while $\:z$, the reference point $\:z\in\:{R}^{M}$, is considered a utopian point in the objective space. The optimized value of $\:{x}_{k}$ represents the KKTPM at the point $\:{\mathrm{x}}^{k}$. The above optimization problem used to compute the KKTPM consists of one quadratic constraint, and one linear constraint, with a total of (M+J + 2) decision variables. In all simulations, we employed MATLAB’s fmincon() routine to solve the optimization problem stated in Eq. (34). Once the optimization is completed, solutions that are theoretically Pareto optimal yield a KKTPM value of zero. Solutions near the Pareto front produce small KKTPM values, while those farther from the front result in larger values.

For infeasible iterations $\:{\mathrm{x}}^{k}$​, the KKTPM value is directly defined based on the total constraint violation. Hence, the KKTPM for any given iteration $\:{\mathrm{x}}^{k}$​ is computed as follows:

38 $$\:\mathrm{K}\mathrm{K}\mathrm{T}\mathrm{P}\mathrm{M}\left({\mathrm{x}}^{k}\right)=\left\{\begin{array}{c}\begin{array}{cc}{x}_{k}^{*},&\:\mathrm{if\:}{\mathrm{x}}^{k}\mathrm{\:is\:feasible}\end{array}\\\:\begin{array}{cc}1+\sum\:_{j=1}^{J}\:{x{g}_{j}\left({\mathrm{x}}^{k}\right)x}^{2},&\:Else\end{array}\end{array}\right.$$

### Results of ZDT and DTLZ test problems

**Fig. 6:**
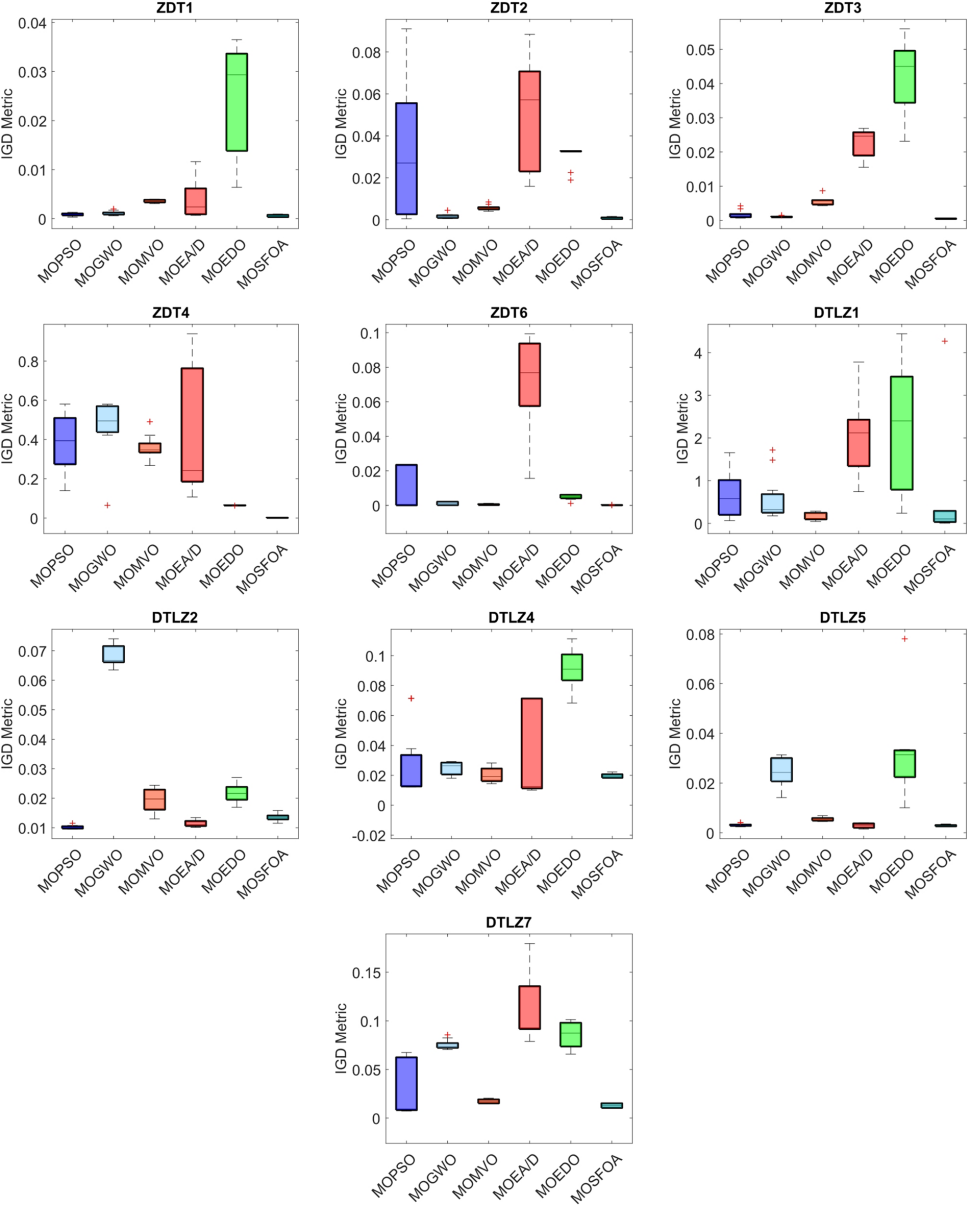
IGD box graph of algorithms on ZDT and DTLZ problems.

The developed MOSFOA approach is initially evaluated using the unconstrained ZDT and DTLZ benchmark suites before being applied to real-world cases. Table 5 reports the IGD metric’s statistical outcomes—average (Ave), standard deviation (SD), and overall rank—for each algorithm. The data show that MOSFOA consistently achieves superior IGD results, underscoring its ability to remain close to the true PF. Specifically, MOSFOA outperforms the other MOO algorithms across most test functions, except DTLZ1 and DTLZ2. In these cases, MOMVO and MOPSO perform slightly better—MOMVO achieves the best result for DTLZ1, while MOPSO excels in DTLZ2. The final section of Table 5 also reports the overall average rankings based on the Friedman test for all benchmark functions. According to these rankings, MOSFOA holds the top position, followed by MOMVO and MOPSO, respectively. Moreover, the consistently low IGD values obtained by MOSFOA—particularly in functions like ZDT3 and ZDT6—underscore its robustness and reliability across diverse optimization scenarios. This is further illustrated by the box plots in Fig. 6. The results confirm that MOSFOA is capable of producing PO solution sets with both strong convergence and good diversity, closely aligned with the true PF, an essential quality in MOO. In sum, MOSFOA achieves the best IGD scores on the majority of benchmark tests, reflecting its exceptional capacity to produce evenly distributed solutions that lie close to the true PF. Although MOMVO and MOPSO occasionally deliver competitive results, MOSFOA consistently demonstrates greater reliability and efficiency in addressing MOO challenges.

**Fig. 7:**
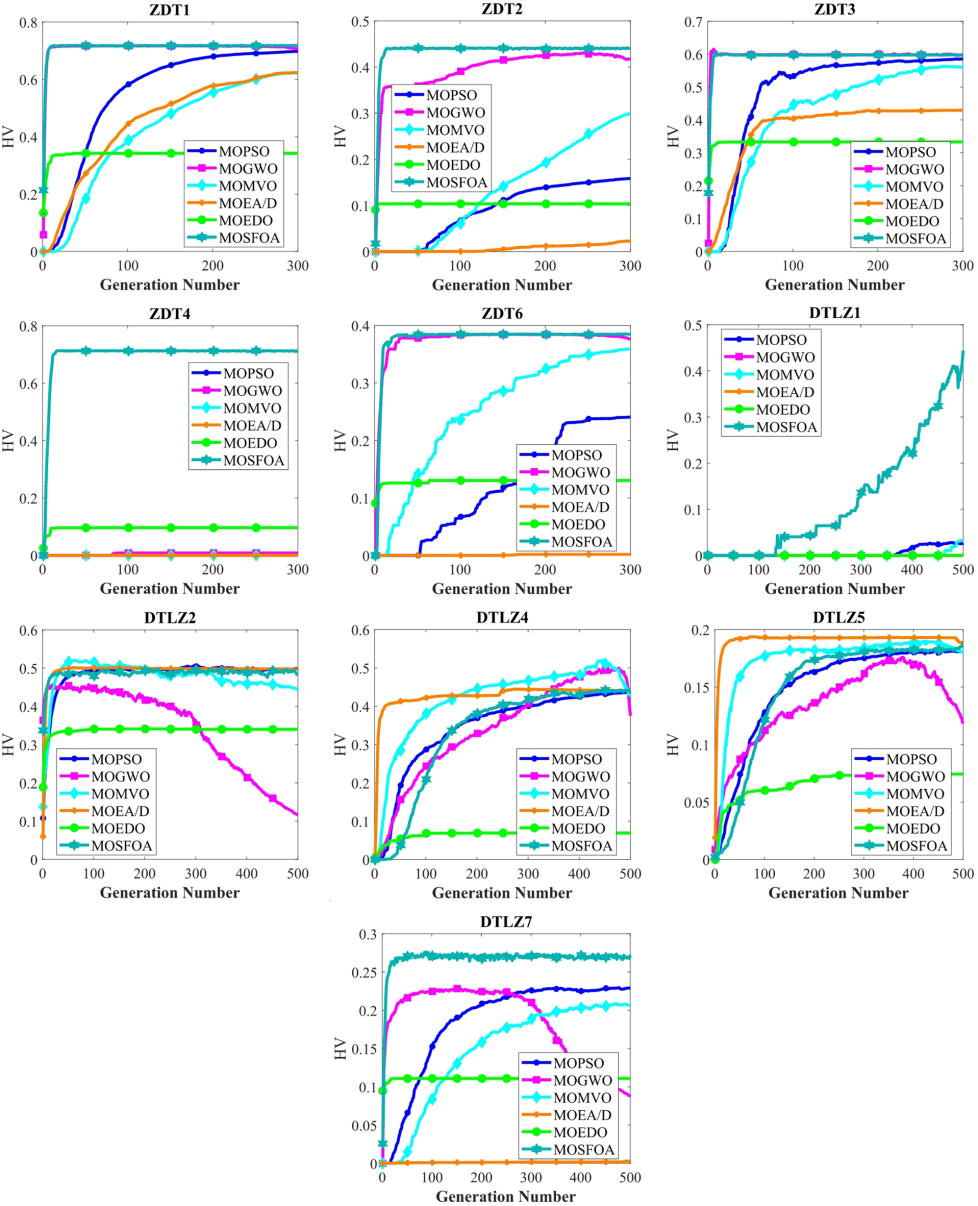
HV convergence behavior of each algorithm.

**Fig. 8:**
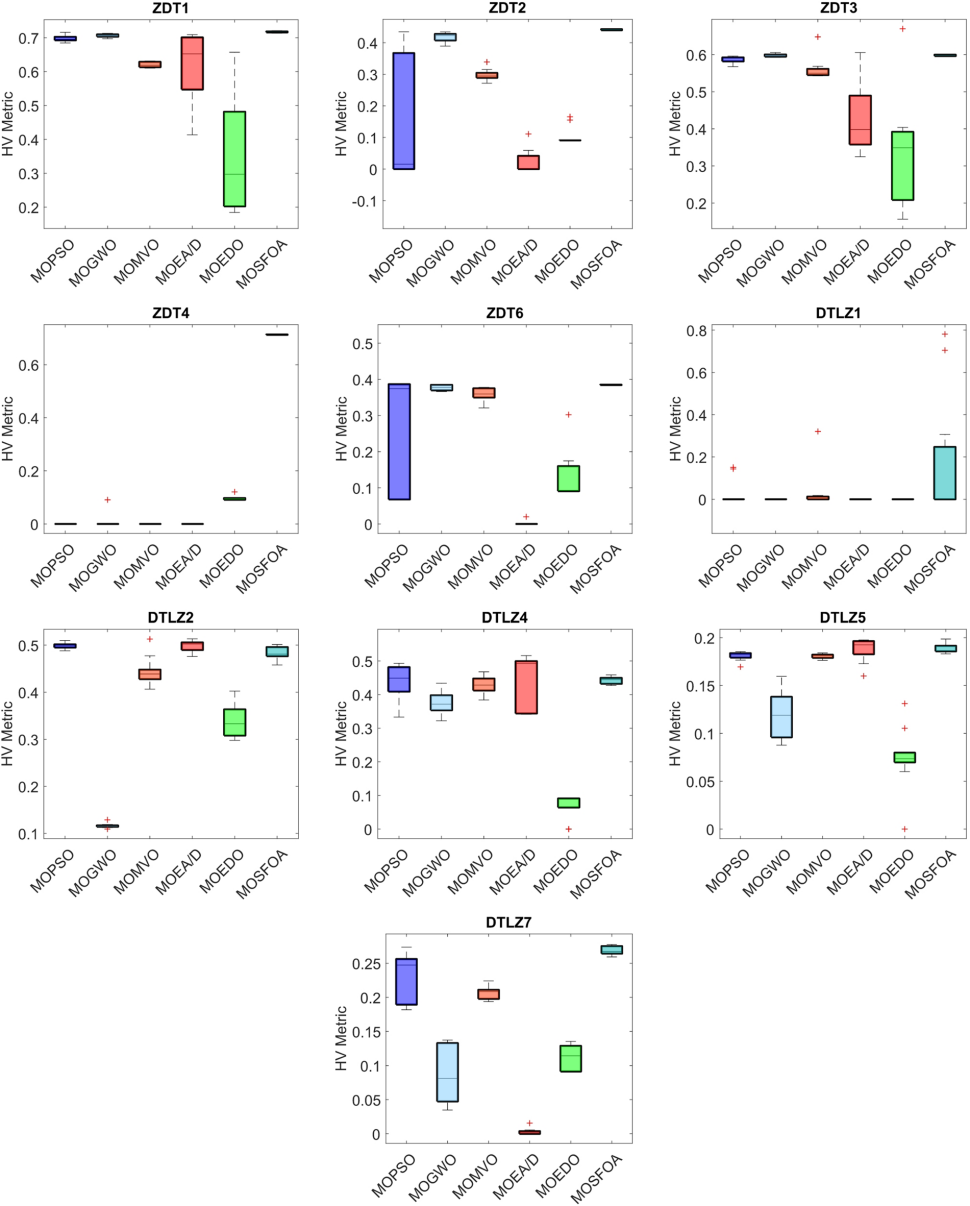
HV box graph of algorithms on ZDT and DTLZ problems.

Table 6 compares the HV outcomes of several algorithms on the ZDT and DTLZ benchmarks, with a particular emphasis on MOSFOA. As an indicator that captures both convergence and diversity by measuring the objective-space volume enclosed by the discovered Pareto front, HV highlights MOSFOA’s strengths: it achieves the highest HV in nine of the ten test problems. The lone exception is DTLZ2, where MOPSO marginally outperforms MOSFOA in terms of both the mean and standard deviation of HV. Across the remaining functions, however, MOSFOA consistently leads the pack, producing well‐distributed solution sets that cover more of the PF. This dominance is reflected in the convergence curves of Fig. 7 and further substantiated by the box plots in Fig. 8, which demonstrate MOSFOA’s stable, high‐quality performance across all cases. While it may not top every single instance, its superior HV scores in 90% of the benchmarks underscore its robustness. These findings confirm that MOSFOA effectively captures a larger share of the Pareto front, making it a dependable choice for tackling complex, real‐world multi‐objective optimization challenges where both thorough exploration and diverse solution sets are crucial.

**Fig. 9:**
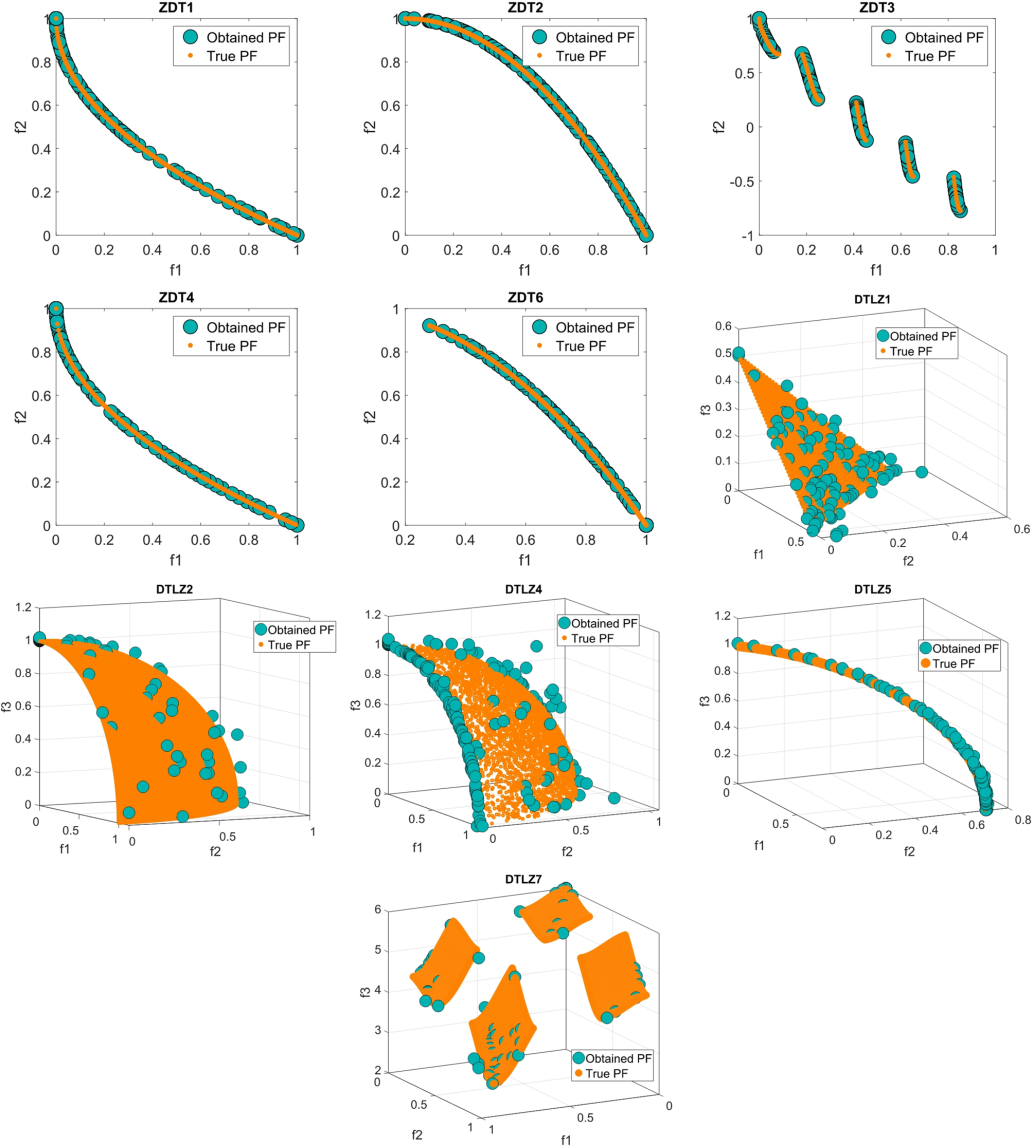
Obtained PF for MOSFOA against the true PF.

Figure 9 illustrates the top ND solutions produced by MOSFOA on the ZDT1–ZDT4 and DTLZ1, DTLZ2, DTLZ4, DTLZ5, and DTLZ7 benchmark sets. The plots clearly demonstrate that the obtained solutions exhibit excellent convergence toward the true PF while maintaining a well-distributed spread across the objective space. This balance between convergence and diversity indicates that MOSFOA effectively avoids premature convergence and successfully explores the search space. Moreover, the smooth and uniform distribution of solutions across different problem types highlights MOSFOA’s adaptability and robustness in handling both convex and non-convex Pareto fronts.

**Table 5:** Statistical findings of IGD-metric on ZDT and DTLZ test functions.

Problem	Index ↓	Algorithms					
		MOPSO	MOGWO	MOMVO	MOEA/D	MOEDO	MOSFOA
ZDT1	Ave	8.621E-04	1.143E-03	3.557E-03	3.638E-03	2.451E-02	5.226E-04
	SD	2.776E-04	4.056E-04	2.832E-04	3.623E-03	1.112E-02	2.479E-04
	Rank	2	3	4	5	6	1
ZDT2	Ave	3.180E-02	1.644E-03	5.603E-03	4.836E-02	3.050E-02	7.632E-04
	SD	3.141E-02	1.108E-03	1.321E-03	2.597E-02	4.912E-03	4.444E-04
	Rank	5	2	3	6	4	1
ZDT3	Ave	1.787E-03	1.063E-03	5.399E-03	2.262E-02	4.226E-02	5.026E-04
	SD	1.090E-03	1.853E-04	1.238E-03	4.010E-03	1.032E-02	6.711E-05
	Rank	3	2	4	5	6	1
ZDT4	Ave	3.818E-01	4.683E-01	3.610E-01	4.261E-01	6.428E-02	9.507E-04
	SD	1.484E-01	1.459E-01	5.764E-02	3.317E-01	5.080E-04	1.366E-04
	Rank	4	6	3	5	2	1
ZDT6	Ave	1.070E-02	1.043E-03	5.194E-04	7.251E-02	4.797E-03	1.703E-04
	SD	1.213E-02	9.197E-04	3.103E-04	2.608E-02	1.504E-03	6.247E-05
	Rank	5	3	2	6	4	1
DTLZ1	Ave	6.622E-01	5.688E-01	1.579E-01	2.086E + 00	2.233E + 00	5.115E-01
	SD	5.293E-01	5.358E-01	8.258E-02	9.354E-01	1.468E + 00	1.252E + 00
	Rank	4	3	1	5	6	2
DTLZ2	Ave	1.016E-02	6.826E-02	1.956E-02	1.142E-02	2.173E-02	1.362E-02
	SD	5.772E-04	3.290E-03	3.768E-03	1.025E-03	2.907E-03	1.245E-03
	Rank	1	6	4	2	5	3
DTLZ4	Ave	2.708E-02	2.473E-02	2.006E-02	3.330E-02	9.110E-02	1.968E-02
	SD	2.320E-02	4.136E-03	5.002E-03	3.013E-02	1.210E-02	1.336E-03
	Rank	4	3	2	5	6	1
DTLZ5	Ave	3.126E-03	2.430E-02	5.430E-03	2.873E-03	3.124E-02	2.872E-03
	SD	5.357E-04	5.887E-03	8.181E-04	8.751E-04	1.719E-02	3.516E-04
	Rank	3	5	4	2	6	1
DTLZ7	Ave	2.860E-02	7.538E-02	1.737E-02	1.141E-01	8.533E-02	1.266E-02
	SD	2.755E-02	4.764E-03	2.015E-03	3.217E-02	1.316E-02	2.330E-03
	Rank	3	4	2	6	5	1
Mean ranking		3.4	3.7	2.9	4.7	5	1.3
Final ranking		3	4	2	5	6	1

**Table 6:** Statistical findings of HV-metric on ZDT and DTLZ test functions.

Problem	Index ↓	Algorithms					
		MOPSO	MOGWO	MOMVO	MOEA/D	MOEDO	MOSFOA
ZDT1	Ave	6.977E-01	7.057E-01	6.206E-01	6.237E-01	3.421E-01	7.172E-01
	SD	8.894E-03	4.858E-03	7.805E-03	9.973E-02	1.607E-01	2.055E-03
	Rank	3	2	5	4	6	1
ZDT2	Ave	1.584E-01	4.171E-01	2.985E-01	2.278E-02	1.035E-01	4.413E-01
	SD	1.915E-01	1.399E-02	1.767E-02	3.657E-02	2.821E-02	2.057E-03
	Rank	5	2	3	4	6	1
ZDT3	Ave	5.859E-01	5.977E-01	5.605E-01	4.294E-01	3.329E-01	5.980E-01
	SD	9.678E-03	3.823E-03	3.013E-02	9.743E-02	1.430E-01	1.653E-03
	Rank	3	2	4	5	6	1
ZDT4	Ave	0.000E + 00	8.264E-03	0.000E + 00	0.000E + 00	9.618E-02	7.131E-01
	SD	0.000E + 00	2.741E-02	0.000E + 00	0.000E + 00	8.691E-03	1.032E-03
	Rank	4	3	4	4	2	1
ZDT6	Ave	2.408E-01	3.764E-01	3.590E-01	1.804E-03	1.304E-01	3.847E-01
	SD	1.651E-01	7.670E-03	1.810E-02	5.984E-03	6.596E-02	4.668E-04
	Rank	4	2	3	6	5	1
DTLZ1	Ave	2.682E-02	0.000E + 00	3.231E-02	0.000E + 00	0.000E + 00	4.439E-01
	SD	5.968E-02	0.000E + 00	9.591E-02	0.000E + 00	0.000E + 00	2.644E-01
	Rank	3	4	2	4	4	1
DTLZ2	Ave	4.991E-01	1.164E-01	4.439E-01	4.976E-01	3.40E-01	4.833E-01
	SD	6.293E-03	4.892E-03	2.908E-02	1.267E-02	3.43E-02	1.410E-02
	Rank	1	6	4	2	5	3
DTLZ4	Ave	4.361E-01	3.751E-01	4.293E-01	4.410E-01	6.926E-02	4.421E-01
	SD	5.717E-02	3.341E-02	2.661E-02	7.815E-02	3.562E-02	1.054E-02
	Rank	3	5	4	2	6	1
DTLZ5	Ave	1.810E-01	1.182E-01	1.808E-01	1.880E-01	7.430E-02	1.887E-01
	SD	4.640E-03	2.556E-02	2.380E-03	1.207E-02	3.168E-02	4.801E-03
	Rank	3	5	4	2	6	1
DTLZ7	Ave	8.741E-02	8.799E-02	2.060E-01	3.076E-03	1.109E-01	2.689E-01
	SD	4.112E-02	4.330E-02	9.683E-03	4.608E-03	1.880E-02	6.041E-03
	Rank	5	4	2	6	3	1
Mean ranking		3.4	3.5	3.5	3.9	4.9	1.2
Final ranking		3	2	2	4	5	1

### Results of engineering test problems

Engineering design problems are critical for evaluating the performance and robustness of multi-objective optimization algorithms. In this study, the effectiveness of the proposed MOSFOA approach is assessed utilizing nine constrained and engineering design benchmark problems: BNH, SRN, OSY, TNK, car, Disk Brake, 4-Bar Truss, CONSTR, and Welded Beam. Each algorithm was independently run 11 times to ensure reliable and fair comparisons.

**Fig. 10:**
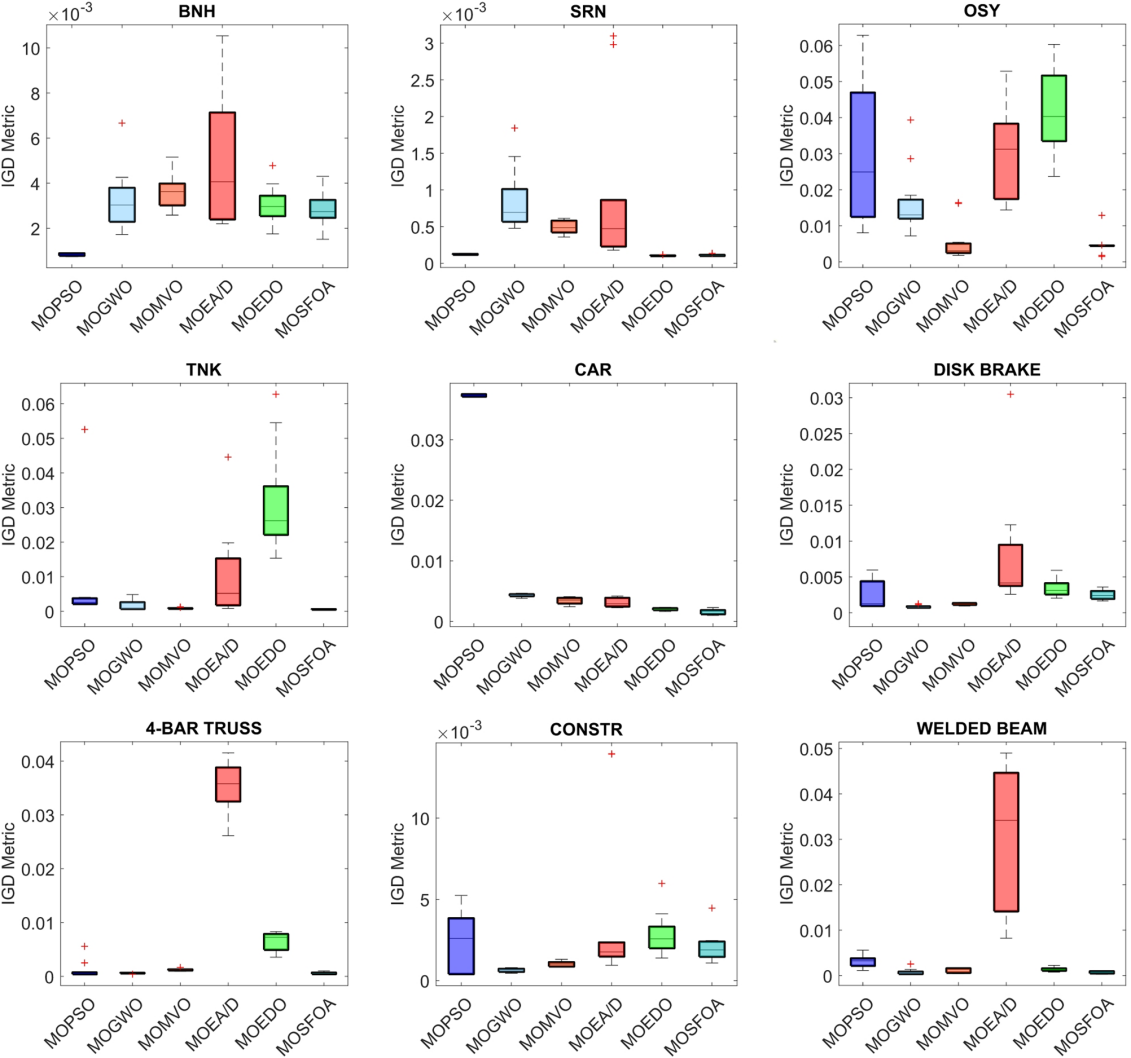
IGD box graph of algorithms on engineering problems.

Table 7 presents the IGD metric results for MOSFOA alongside five competitive algorithms: MOPSO, MOGWO, MOMVO, MOEA/D, and MOEDO. The results reveal that MOSFOA outperforms all competing algorithms in at least 5 out of the 9 benchmark problems. For the remaining cases, MOSFOA remains highly competitive, achieving second place in two problems (BNH and SRN), third place in one (CONSTR), and fourth in the Disk Brake problem. Overall, MOSFOA secures the top position based on the average Friedman ranking, with a score of 1.8. It is followed by MOGWO (2.9), MOMVO (3.1), MOPSO (3.7), MOEDO (4.1), and MOEA/D (5.7), respectively. These rankings indicate the superior overall performance of MOSFOA across various constrained engineering design scenarios. Furthermore, the box plot analysis in Fig. 10 illustrates the distribution and variability of the obtained results. MOSFOA exhibits robust and stable performance, particularly in complex problems such as SRN, OSY, TNK, 4-Bar Truss, and Welded Beam design. The algorithm consistently achieves low average fitness values and minimal variance across multiple runs, indicating its reliability and precision. These findings emphasize MOSFOA’s capability to generate high-quality solutions while maintaining strong stability and consistency—key characteristics for addressing real-world engineering design challenges.

**Fig. 11:**
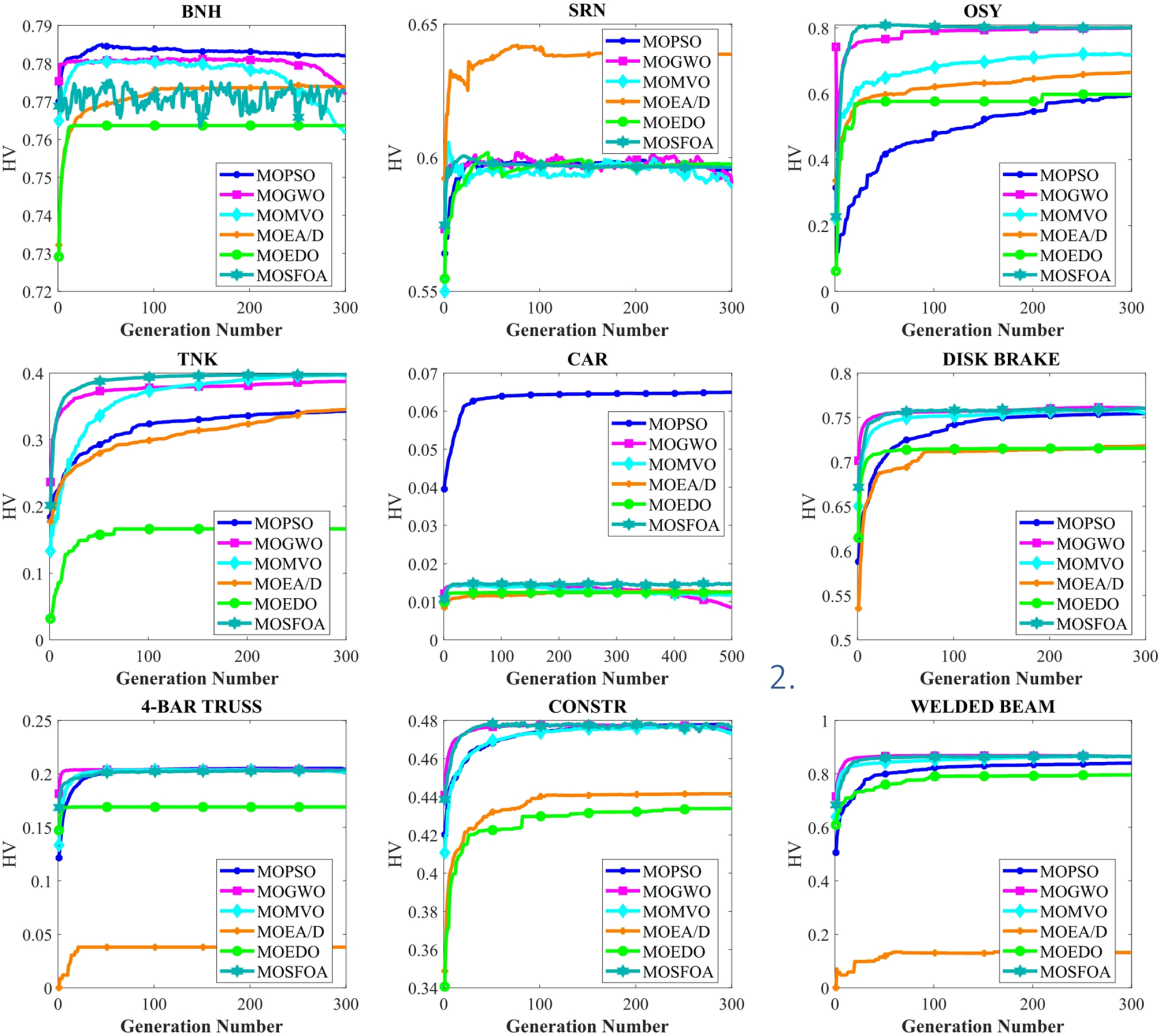
HV convergence behavior of each algorithm.

**Fig. 12:**
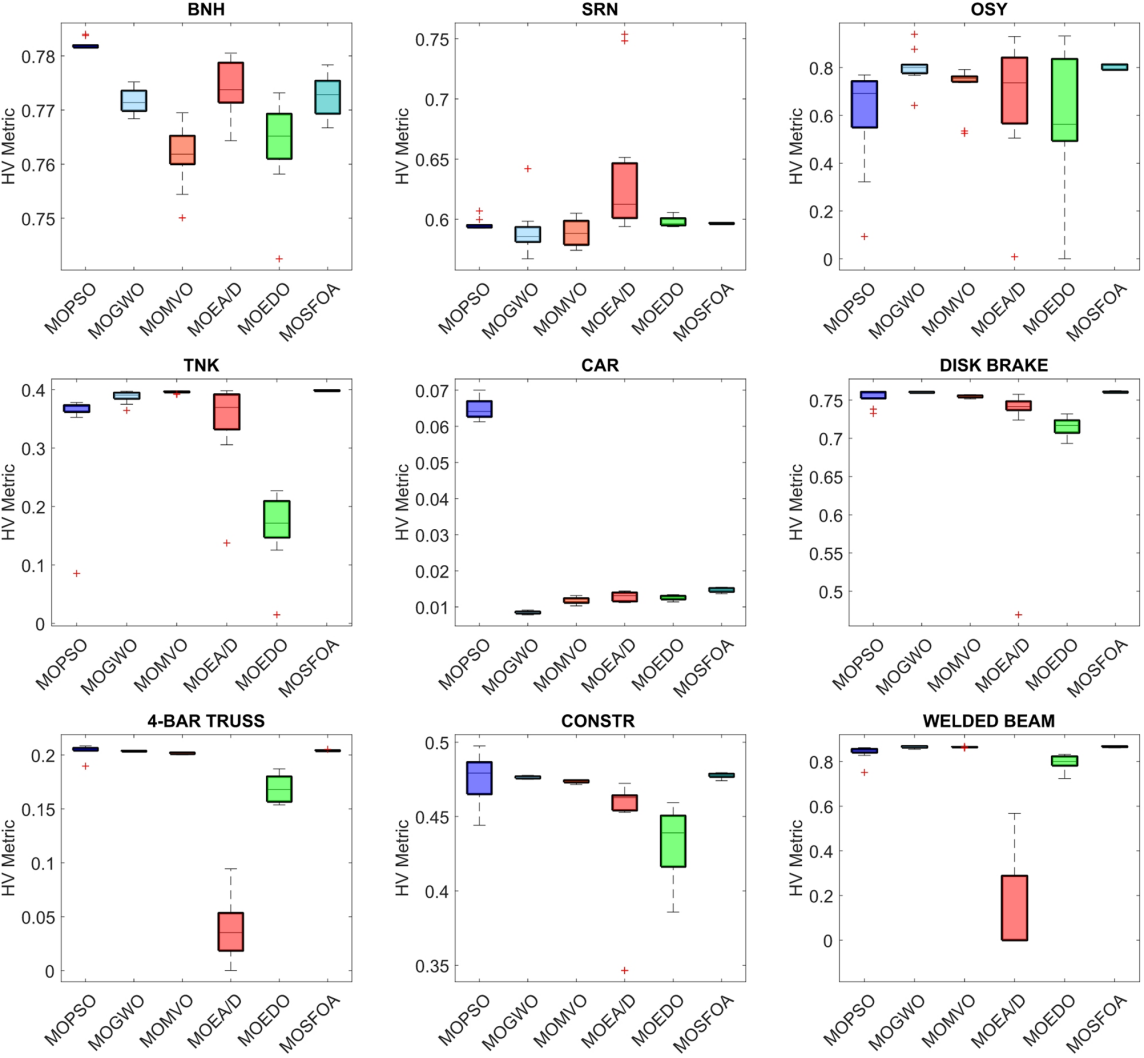
HV box graph of algorithms on engineering problems.

Table 8 displays the HV results for constrained and engineering benchmark problems, emphasizing the outstanding performance of the developed MOSFOA method relative to other methods. Based on the HV metric, MOSFOA achieves the best findings in six out of nine test instances, including OSY, TNK, Disk Brake, 4-Bar Truss, and Welded Beam. MOPSO ranks second overall, achieving the highest HV scores in two problems—BNH and Car. The overall average Friedman rankings provided in Table 8 further support these findings. MOSFOA ranks first, confirming its strong and consistent performance. MOPSO and MOGWO share the second rank, while MOEA/D and MOMVO are tied for third place. MOEDO ranks last among the compared methods. Figure 11 displays the convergence behavior of the algorithms, showing that MOSFOA attains better convergence accuracy than its counterparts. The convergence curves confirm that MOSFOA consistently guides the search process toward the global optimum, even in complex optimization landscapes. Additionally, the boxplot analysis in Fig. 12 emphasizes the robustness and reliability of MOSFOA. The approach exhibits a noticeably narrower distribution of HV values, indicating stable and repeatable performance across all test functions. This further reinforces its strength in handling intricate engineering design problems. In conclusion, the results underscore the effectiveness of the proposed MOSFOA in addressing complex MOO challenges. Its consistent superiority in HV performance, convergence accuracy, and result stability positions it as a highly promising technique, outperforming many existing methods in the literature.

**Fig. 13:**
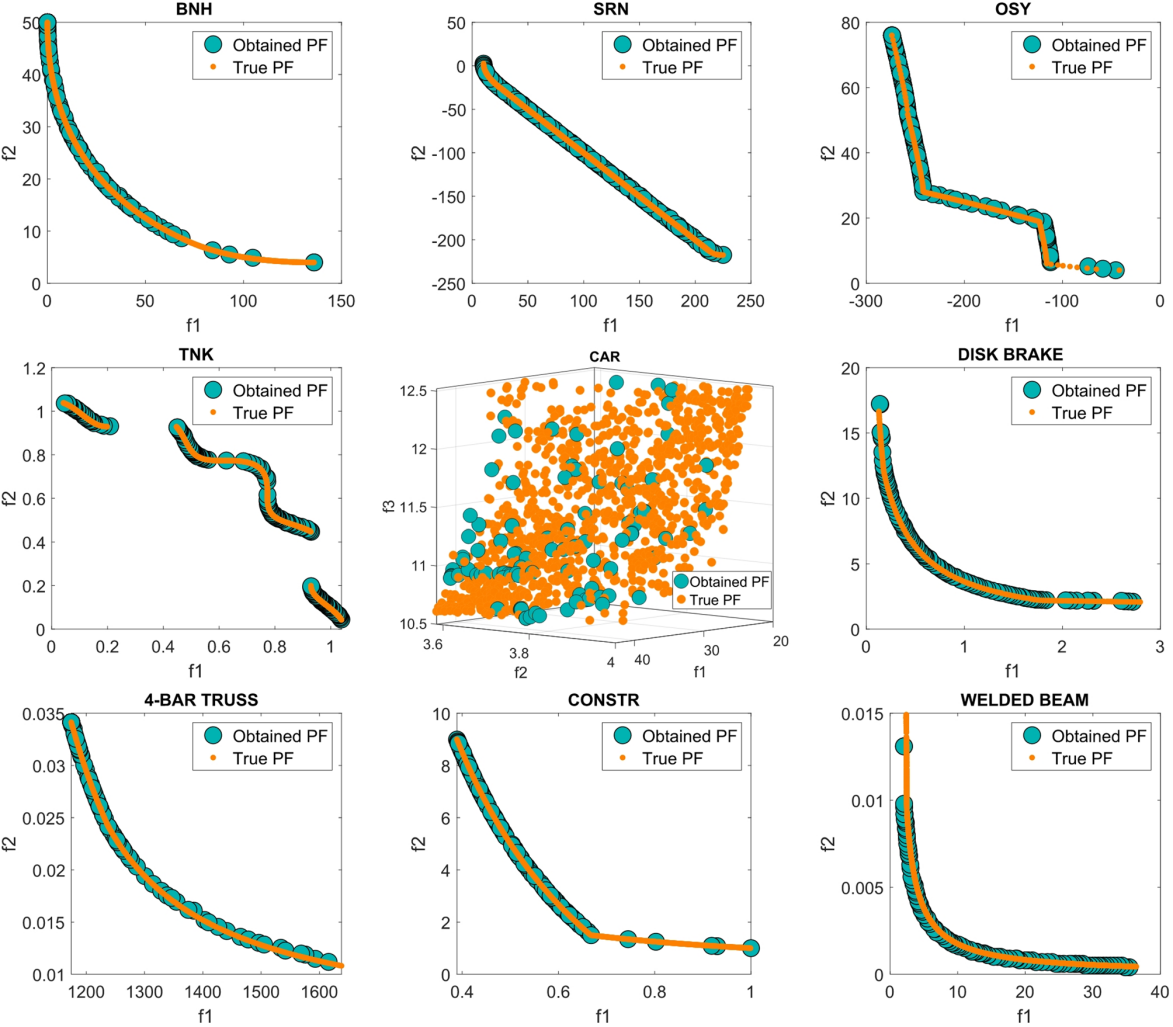
Obtained PF for MOSFOA against the true PF.

Figure 13 presents the best PO solutions produced by the proposed MOSFOA approach for the constrained and engineering benchmark problems, namely BNH, SRN, OSY, CAR, Disk Brake, 4-Bar Truss, CONSTR, and Welded Beam. The visualizations clearly demonstrate MOSFOA’s strong convergence behavior, as the obtained solutions closely align with the true PO fronts, reflecting high computational accuracy and stability. Moreover, the even distribution of solutions along the PO indicates the algorithm’s effectiveness in maintaining diversity under constraint conditions. These results further confirm MOSFOA’s capability to balance convergence and diversity in solving complex real-world engineering optimization problems.

**Table 7:** Statistical findings of IGD-metric on the engineering problems.

Problem	Index ↓	Algorithms					
		MOPSO	MOGWO	MOMVO	MOEA/D	MOEDO	MOSFOA
BNH	Ave	8.303E-04	3.255E-03	3.676E-03	4.833E-03	3.075E-03	2.834E-03
	SD	5.965E-05	1.379E-03	7.963E-04	2.863E-03	8.224E-04	7.314E-04
	Rank	1	4	5	6	3	2
SRN	Ave	1.211E-04	8.573E-04	4.937E-04	9.376E-04	1.033E-04	1.085E-04
	SD	6.788E-06	4.322E-04	8.510E-05	1.069E-03	5.700E-06	1.272E-05
	Rank	3	5	4	6	1	2
OSY	Ave	2.824E-02	1.624E-02	5.490E-03	3.013E-02	4.197E-02	4.740E-03
	SD	1.955E-02	9.552E-03	5.449E-03	1.233E-02	1.198E-02	2.932E-03
	Rank	4	3	2	5	6	1
TNK	Ave	7.256E-03	1.682E-03	8.654E-04	1.049E-02	3.126E-02	5.566E-04
	SD	1.504E-02	1.463E-03	1.816E-04	1.301E-02	1.489E-02	6.515E-05
	Rank	4	3	2	5	6	1
CAR	Ave	3.726E-02	4.297E-03	3.385E-03	3.122E-03	1.972E-03	1.604E-03
	SD	1.959E-04	2.320E-04	5.813E-04	7.336E-04	2.216E-04	4.537E-04
	Rank	6	5	4	3	2	1
DISK BRAKE	Ave	2.438E-03	8.526E-04	1.189E-03	7.936E-03	3.389E-03	2.495E-03
	SD	2.000E-03	1.862E-04	1.596E-04	8.119E-03	1.137E-03	6.362E-04
	Rank	3	1	2	6	5	4
4-BAR TRUSS	Ave	1.175E-03	6.007E-04	1.220E-03	3.504E-02	6.441E-03	5.804E-04
	SD	1.583E-03	9.545E-05	1.818E-04	4.787E-03	1.752E-03	1.962E-04
	Rank	3	2	4	6	5	1
CONSTR	Ave	2.262E-03	6.324E-04	1.011E-03	3.943E-03	2.820E-03	2.081E-03
	SD	1.866E-03	1.121E-04	1.577E-04	4.965E-03	1.323E-03	9.268E-04
	Rank	4	1	2	6	5	3
WELDED BEAM	Ave	3.041E-03	7.884E-04	1.060E-03	3.099E-02	1.325E-03	7.209E-04
	SD	1.316E-03	6.617E-04	4.889E-04	1.580E-02	4.280E-04	2.405E-04
	Rank	5	2	3	6	4	1
Mean ranking		3.7	2.9	3.1	5.4	4.1	1.8
Final ranking		4	2	3	6	5	1

**Table 8:** Statistical findings of HV-metric on the engineering problems.

Problem	Index ↓	Algorithms					
		MOPSO	MOGWO	MOMVO	MOEA/D	MOEDO	MOSFOA
BNH	Ave	7.821E-01	7.616E-01	7.609E-01	7.739E-01	7.636E-01	7.726E-01
	SD	8.936E-04	5.473E-03	6.666E-03	5.536E-03	8.401E-03	3.663E-03
	Rank	1	5	6	2	4	3
SRN	Ave	5.956E-01	5.903E-01	5.887E-01	6.388E-01	5.976E-01	5.964E-01
	SD	4.183E-03	1.925E-02	1.074E-02	5.788E-02	3.877E-03	3.932E-04
	Rank	4	5	6	1	2	3
OSY	Ave	5.950E-01	8.000E-01	7.175E-01	6.653E-01	5.984E-01	8.005E-01
	SD	2.112E-01	7.263E-02	9.369E-02	2.592E-01	2.698E-01	1.115E-02
	Rank	6	2	3	4	5	1
TNK	Ave	3.433E-01	3.875E-01	3.956E-01	3.453E-01	1.661E-01	3.980E-01
	SD	8.587E-02	9.909E-03	1.747E-03	7.519E-02	5.937E-02	6.734E-04
	Rank	5	3	2	4	6	1
CAR	Ave	6.497E-02	8.441E-03	1.173E-02	1.274E-02	1.246E-02	1.464E-02
	SD	2.822E-03	3.459E-04	9.174E-04	1.275E-03	6.755E-04	5.732E-04
	Rank	1	6	5	3	4	2
DISK BRAKE	Ave	7.544E-01	7.601E-01	7.546E-01	7.180E-01	7.155E-01	7.604E-01
	SD	9.955E-03	7.189E-04	1.797E-03	8.293E-02	1.246E-02	8.893E-04
	Rank	4	2	3	5	6	1
4-BAR TRUSS	Ave	2.041E-01	2.035E-01	2.014E-01	3.794E-02	1.690E-01	2.042E-01
	SD	4.994E-03	3.991E-04	6.213E-04	2.644E-02	1.246E-02	9.153E-04
	Rank	2	3	4	6	5	1
CONSTR	Ave	4.754E-01	4.763E-01	4.735E-01	4.416E-01	4.339E-01	4.773E-01
	SD	1.677E-02	7.950E-04	9.168E-04	4.733E-02	2.275E-02	1.789E-03
	Rank	3	2	4	5	6	1
WELDED BEAM	Ave	8.402E-01	8.650E-01	8.639E-01	1.317E-01	7.962E-01	8.658E-01
	SD	3.121E-02	4.206E-03	1.758E-03	2.294E-01	3.314E-02	2.333E-03
	Rank	4	2	3	6	5	1
Mean ranking		3.3	3.3	4	4	4.8	1.6
Final ranking		2	2	3	3	4	1

### Wilcoxon rank-sum

The Wilcoxon Rank-Sum Test (WRT)^74^ is a non-parametric statistical method applied at a 5% significance level. It is used to assess whether two or more datasets come from the same distribution, making it particularly suitable for comparing the performance of optimization algorithms without requiring the assumption of normality.

In this study, the WRT is employed to conduct pairwise comparisons of performance metrics between the introduced methods and its competitors. The null hypothesis asserts that there is no significant difference in the mean performance metrics of the two methods being compared, implying equivalent performance. Conversely, the alternative hypothesis posits that the mean values differ, indicating a performance difference. To summarize the comparison results, the following symbols are used: “+” indicates that the proposed algorithm significantly outperforms the compared algorithm, “–” indicates that the introduced approach performs significantly worse, and “=” denotes no statistically significant difference in performance.

Table 9 displays the *p*-values derived from the Wilcoxon test for each benchmark function based on the IGD metric, revealing that the proposed MOSFOA algorithm significantly outperforms most of its competitors. In particular, MOSFOA demonstrates superior performance over MOPSO in most cases, with only one instance where MOPSO performs significantly better. Similarly, when compared with MOGWO, MOSFOA shows better performance in most functions, although there are a few cases where MOGWO slightly outperforms it. Compared to the remaining algorithms, MOSFOA consistently performs better across all test functions.

Regarding the HV metric, Table 10 shows that MOSFOA continues to deliver excellent results, outperforming other algorithms in most test functions. The *p*-values indicate statistically significant improvements over nearly all competing methods, except in a few cases where the differences are not statistically significant, specifically, in three functions when compared with MOPSO and in one function when compared with MOEA/D. In comparison to the other algorithms, MOSFOA demonstrates superior performance across all benchmark functions, achieving better convergence toward the true Pareto Front and maintaining higher diversity among solutions. This consistent superiority highlights the algorithm’s strong balance between exploration and exploitation.

Broadly speaking, based on the data in Tables 9 and 10, and considering a total of 190 cases (derived from 19 functions evaluated across 5 algorithms and 2 metrics), MOSFOA demonstrates significantly better performance in 145 cases. In 38 cases, the performance was statistically equivalent to the compared algorithms, and in only 7 cases did MOSFOA perform significantly worse. These outcomes confirm the effectiveness and robustness of the introduced MOSFOA algorithm across a wide range of unconstrained, constrained, and engineering optimization problems.

**Table 9:** *p*-values of Wilcoxon test of IGD metric.

Problems ↓	Algorithms				
	MOPSO	MOGWO	MOMVO	MOEA/D	MOEDO
ZDT1	1.512E-02/+	8.113E-04/+	8.162E-05/+	5.010E-04/+	8.162E-05/+
ZDT2	2.027E-03/+	1.512E-02/+	8.162E-05/+	8.162E-05/+	6.908E-05/+
ZDT3	8.162E-05/+	8.162E-05/+	8.162E-05/+	8.162E-05/+	8.162E-05/+
ZDT4	8.162E-05/+	8.162E-05/+	8.162E-05/+	8.162E-05/+	8.162E-05/+
ZDT6	4.306E-01/=	2.557E-02/+	2.358E-04/+	8.152E-05/+	8.162E-05/+
DTLZ1	2.155E-02/+	2.557E-02/+	8.438E-01/=	1.293E-03/+	2.523E-03/+
DTLZ2	8.152E-05/+	8.152E-05/+	8.113E-04/+	6.388E-04/+	8.152E-05/+
DTLZ4	4.701E-01/=	1.044E-02/+	5.114E-01/=	2.934E-01/=	8.152E-05/+
DTLZ5	3.579E-01/=	8.152E-05/+	8.152E-05/+	6.694E-01/=	8.152E-05/+
DTLZ7	4.307E-01/=	8.152E-05/+	8.113E-04/+	8.152E-05/+	8.152E-05/+
BNH	8.152E-05/-	6.936E-01/=	1.044E-02/+	2.372E-01/=	3.933E-01/=
SRN	1.044E-02/+	8.152E-05/+	8.152E-05/+	8.152E-05/+	4.307E-01/=
OSY	2.358E-04/+	1.819E-04/+	3.933E-01/=	8.152E-05/+	8.152E-05/+
TNK	8.152E-05/+	5.010E-04/+	8.152E-05/+	8.152E-05/+	8.152E-05/+
CAR	8.152E-05/+	8.152E-05/+	8.152E-05/+	1.398E-04/+	3.562E-02/+
DISK BRAKE	2.372E-01/=	8.152E-05/-	8.152E-05/+	6.388E-04/+	4.884E-02/+
4-BAR TRUSS	7.928E-01/=	2.934E-01/=	1.070E-04/+	8.152E-05/+	8.152E-05/+
CONSTR	1.000E + 00/=	8.152E-05/-	3.043E-04/+	8.955E-01/=	1.150E-01/=
WELDED BEAM	8.152E-05/+	4.307E-01/=	2.122E-01/=	8.152E-05/+	1.293E-03/+
+/=/-	11/7/1	14/3/2	15/4/0	15/4/0	16/3/0

**Table 10:** *p*-values of Wilcoxon test of HV metric.

Problems ↓	Algorithms				
	MOPSO	MOGWO	MOMVO	MOEA/D	MOEDO
ZDT1	1.819E-4/+	8.152E-05/+	8.152E-05/+	8.152E-05/+	8.152E-05/+
ZDT2	7.422E-5/+	8.152E-05/+	8.152E-05/+	6.236E-05/+	6.908E-05/+
ZDT3	1.070E-4/+	6.458E-01/=	1.293E-03/+	1.293E-03/+	1.293E-03/+
ZDT4	2.553E-05/+	3.513E-05/+	2.553E-05/+	2.553E-05/+	8.152E-05/+
ZDT6	7.426E-01/=	1.808E-02/+	8.152E-05/+	3.513E-05/+	8.152E-05/+
DTLZ1	9.742E-04/+	3.063E-04/+	1.916E-03/+	3.063E-04/+	3.063E-04/+
DTLZ2	1.044E-02/-	8.152E-05/+	3.127E-03/+	3.024E-02/+	8.152E-05/+
DTLZ4	6.458E-01/=	2.358E-04/+	2.122E-01/=	2.934E-01/=	8.114E-05/+
DTLZ5	3.913E-04/+	8.152E-05/+	1.398E-04/+	4.307E-01/=	8.152E-05/+
DTLZ7	6.388E-04/+	8.152E-05/+	8.152E-05/+	7.422E-05/+	8.152E-05/+
BNH	8.152E-05/-	6.458E-01/=	1.819E-04/+	4.701E-01/=	3.127E-03/+
SRN	1.259E-02/+	1.259E-02/+	7.624E-02/=	1.293E-03/-	7.928E-01/=
OSY	8.152E-05/+	6.936E-01/=	3.043E-04/+	7.624E-02/=	7.624E-02/=
TNK	8.152E-05/+	1.070E-04/+	1.819E-04/+	3.913E-04/+	8.162E-05/+
CAR	8.152E-05/-	8.152E-05/+	8.152E-05/+	1.293E-03/+	8.162E-05/+
DISK BRAKE	7.624E-02/=	3.579E-01/=	8.152E-05/+	8.152E-05/+	8.162E-05/+
4-BAR TRUSS	2.147E-02/+	3.015E-02/+	8.076E-05/+	8.076E-05/+	8.076E-05/+
CONSTR	6.936E-01/=	3.562E-02/+	6.388E-04/+	8.152E-05/+	8.162E-05/+
WELDED BEAM	1.392E-04/+	1.000E + 00/=	2.553E-02/+	5.391E-05/+	8.114E-05/+
+/=/-	12/4/3	14//5/0	17/2/0	14/4/1	17/2/0

### Evaluating the performance of algorithms using KKT proximity metric

In this section, the performance of the proposed MOSFOA is evaluated and compared with five competitive approaches using KKT proximity metric. This metric is employed to assess convergence quality without requiring knowledge of the true PF. Eight benchmark problems are considered: ZDT1, ZDT2, ZDT3, ZDT6, DTLZ5, SRN, TNK, and the welded beam design problem.

Table 11 reports the KKTPM results for MOSFOA alongside five competitive algorithms: MOPSO, MOGWO, MOMVO, MOEA/D, and MOEDO. The proposed MOSFOA achieved the best performance in four out of the eight problems, specifically ZDT2, ZDT6, TNK, and the welded beam design problem. It ranked second in ZDT1 and SRN. The overall Friedman mean ranking presented in the last part of Table 11 further confirms the strong performance of MOSFOA, which attained the best average rank of 1.88. It is followed by MOGWO and MOEDO, with mean ranks of 2.50 and 3.63, respectively.

**Fig. 14:**
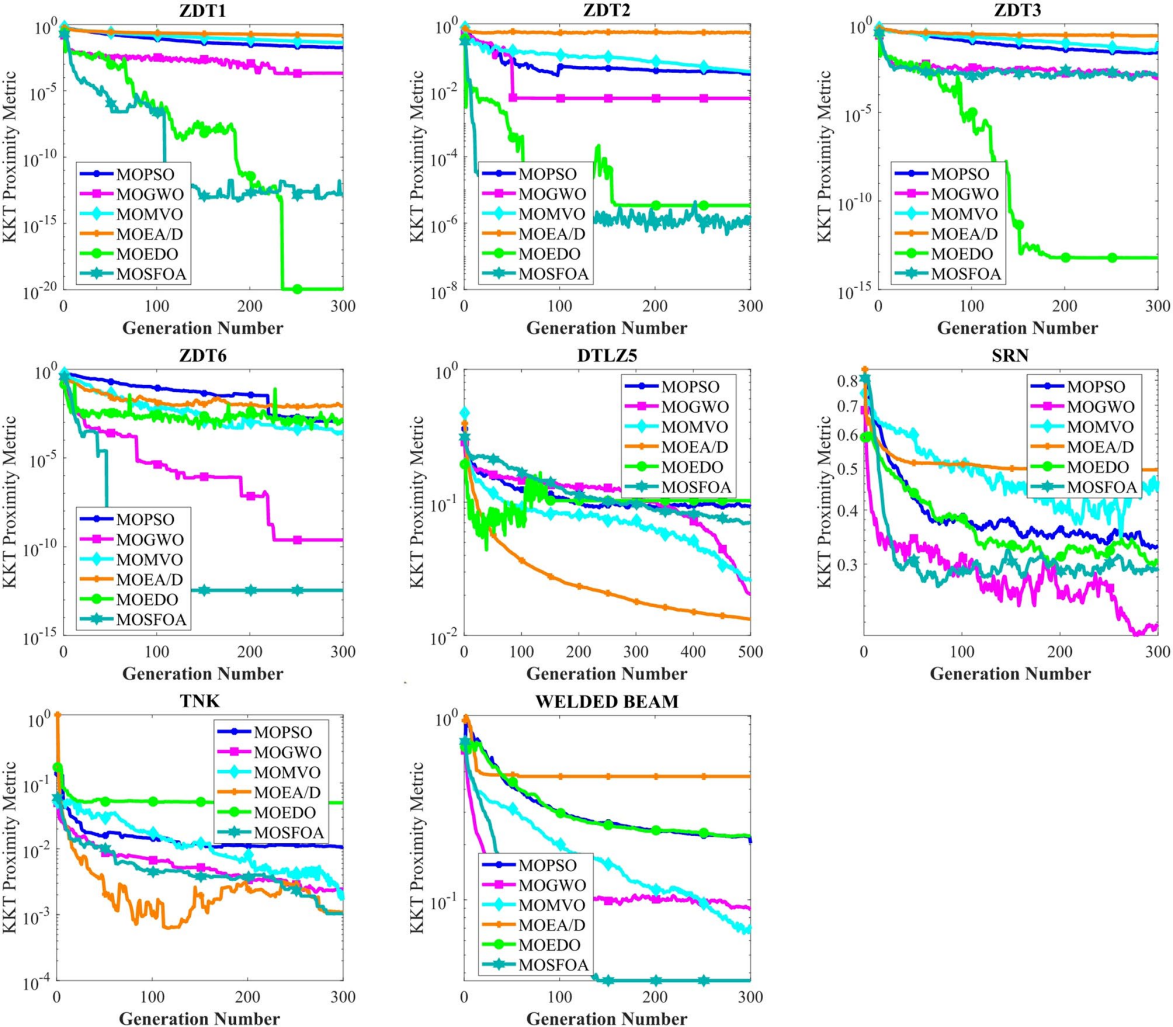
KKTPM convergence behavior of each algorithm.

**Fig. 15:**
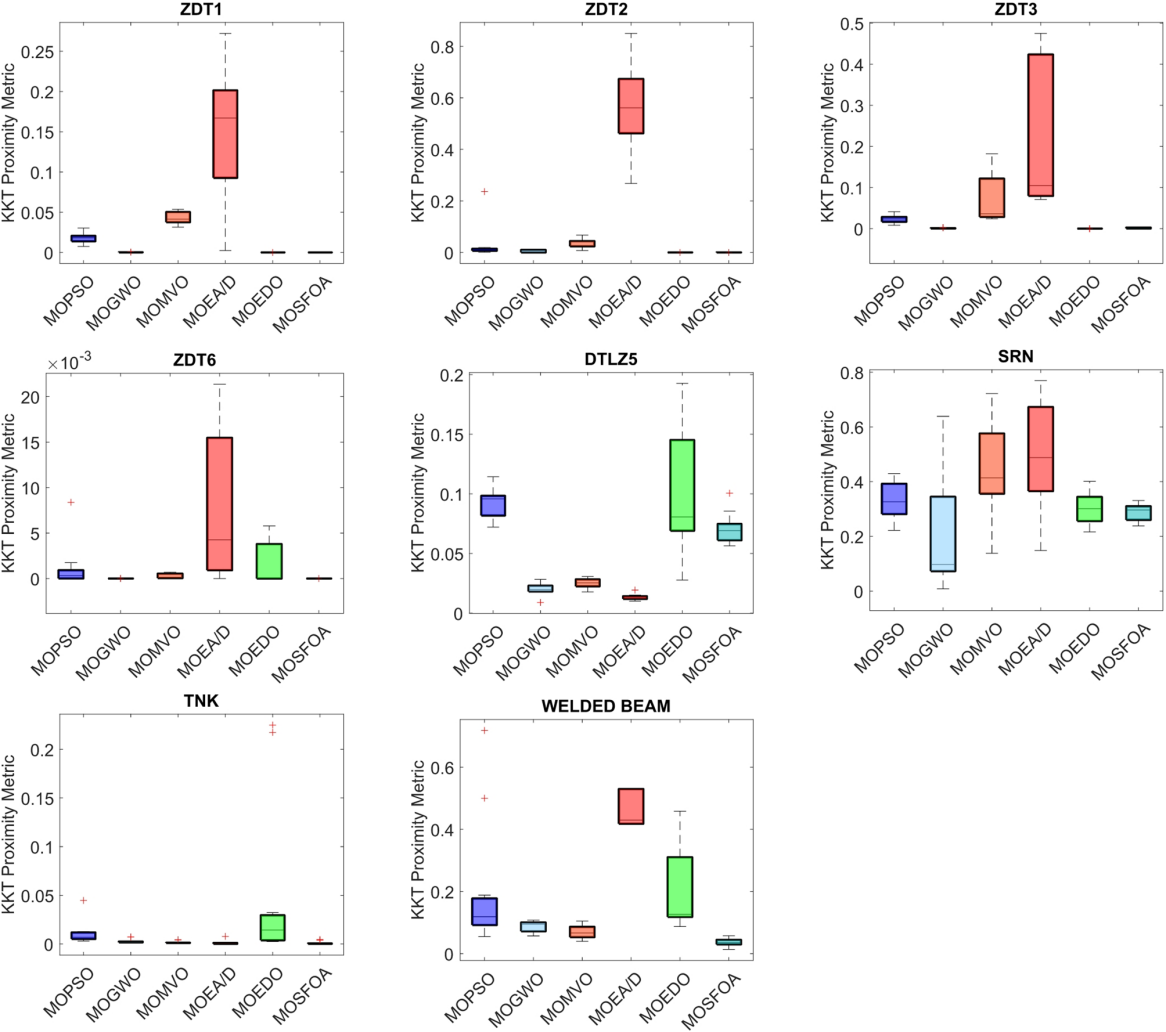
KKTPM box graph of algorithms on selected problems.

To provide a clearer visual understanding of convergence behavior, Fig. 14 demonstrates the convergence curves of MOSFOA compared with the other algorithms across the eight selected problems. Additionally, the box plots in Fig. 15 further highlight the performance variations. The results shown that MOSFOA is capable of generating PO solution sets with strong convergence characteristics that closely align with the true PF, which is a crucial requirement in MOO.

**Fig. 16:**
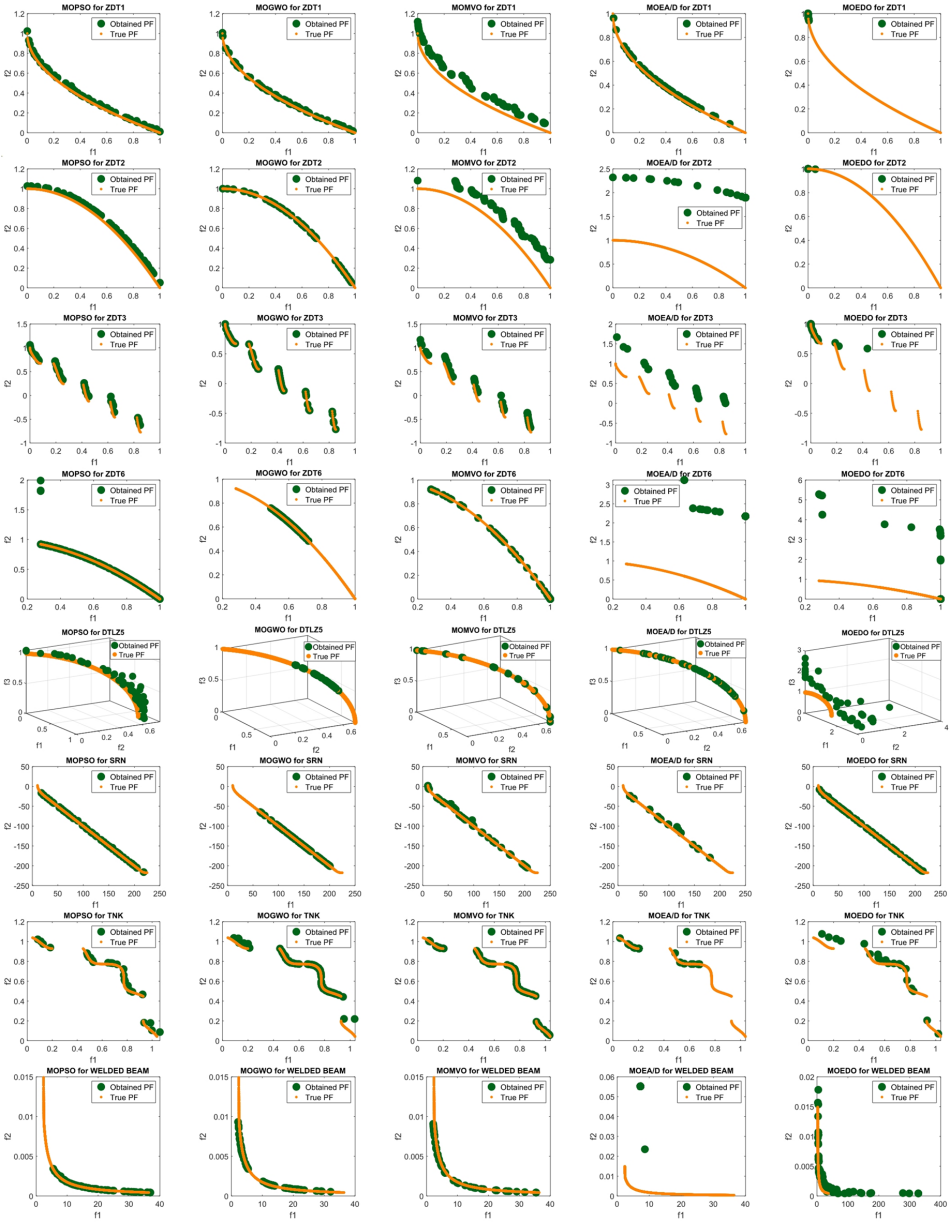
Obtained PF produced by competitive algorithms on selected problems.

It is worth noting that although some algorithms, such as MOEDO, outperformed MOSFOA in terms of convergence for specific cases like ZDT1 and ZDT3, they exhibited noticeably weak distribution quality. This limitation is evident in the IGD and HV findings reported in Tables 5 and 6, as well as in Tables 7 and 8. Moreover, Fig. 16 presents the nondominated solutions generated by the algorithms competing with MOSFOA. Overall, the findings highlight the superiority of the introduced algorithm compared to competing algorithms. Furthermore, the proposed MOSFOA algorithm shows relatively narrow box plots, indicating lower variability and stronger performance consistency across most benchmark functions.

**Table 11:** Statistical findings of KKTPM metric on selected problems.

Problem	Index ↓	Algorithms					
		MOPSO	MOGWO	MOMVO	MOEA/D	MOEDO	MOSFOA
ZDT1	Ave	1.801E-02	2.126E-04	4.316E-02	1.524E-01	1.102E-20	1.482E-13
	SD	6.396E-03	1.321E-04	7.247E-03	9.129E-02	1.028E-23	1.370E-13
	Rank	4	3	5	6	1	2
ZDT2	Ave	3.061E-02	5.74E-03	3.685E-02	5.573E-01	3.407E-06	1.421E-06
	SD	6.839E-02	4.62E-03	1.846E-02	1.611E-01	6.051E-21	1.578E-06
	Rank	4	3	5	6	2	1
ZDT3	Ave	2.278E-02	8.174E-04	6.774E-02	2.122E-01	6.257E-14	1.255E-03
	SD	9.106E-03	7.330E-04	5.981E-02	1.767E-01	6.453E-20	1.208E-03
	Rank	4	2	5	6	1	3
ZDT6	Ave	1.145E-03	2.421E-10	2.648E-04	7.948E-03	1.619E-03	3.469E-13
	SD	2.460E-03	8.028E-10	2.611E-04	7.894E-03	2.484E-03	1.145E-12
	Rank	4	2	3	6	5	1
DTLZ5	Ave	9.258E-02	2.042E-02	2.539E-02	1.323E-02	1.029E-01	7.067E-02
	SD	1.222E-02	5.144E-03	3.843E-03	2.493E-03	5.304E-02	1.290E-02
	Rank	5	2	3	1	6	4
SRN	Ave	3.307E-01	2.172E-01	4.670E-01	4.957E-01	3.056E-01	2.892E-01
	SD	6.523E-02	1.988E-01	1.850E-01	2.044E-01	6.238E-02	3.234E-02
	Rank	4	1	5	6	3	2
TNK	Ave	1.086E-02	2.429E-03	1.671E-03	1.096E-03	5.003E-02	1.033E-03
	SD	1.170E-02	1.658E-03	1.286E-03	2.246E-03	8.512E-02	1.610E-03
	Rank	5	4	3	2	6	1
WELDED BEAM	Ave	2.033E-01	8.855E-02	6.931E-02	4.700E-01	2.23E-01	3.626E-02
	SD	2.094E-01	1.754E-02	2.046E-02	5.743E-02	1.35E-01	1.179E-02
	Rank	4	3	2	6	5	1
Mean ranking		4.25	2.50	3.88	4.88	3.63	1.88
Final ranking		5	2	4	6	3	1

### Application to real‑world test cases

**Fig. 17:**
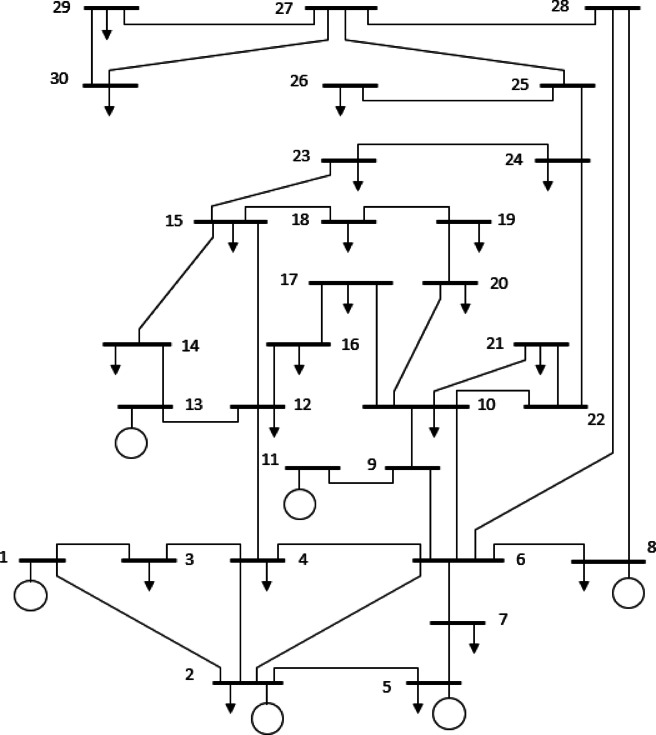
Single line diagram for IEEE 30-bus test system.

In this section, we apply both the original SFOA and its multi‑objective extension, MOSFOA, to the OPF problem on the IEEE 30‑Bus network (see Fig. 17), which comprises 41 transmission lines, six generators, and four transformers. All relevant system data, bus parameters, line impedances, load demands, and generator cost coefficients are taken from^75^.

We design eight case studies on this system (summarized in Table 12) to assess algorithm performance:

1. Single‑objective scenarios (4 cases):

- Minimize fuel cost (FC).
- Minimize emissions (EM).
- Minimize active power losses (PL).
- Minimize voltage deviation (VD).

2. Bi‑objective scenarios (3 cases):

- Minimize FC and EM simultaneously.
- Minimize FC and PL simultaneously.
- Minimize FC and VD simultaneously.

3. Tri‑objective scenario (1 case):

- Minimize FC, EM, and PL together.

Mathematical formulations for each objective are provided in Sect. 3. For all experiments, we use a population size of 100. The maximum number of generations is set to 300 for both single- and bi-objective OPF tasks, and 500 for the tri-objective case.

#### Single-objective OPF

In the SOO cases, the proposed SFOA is evaluated against two categories of optimization algorithms. The first category comprises well-established methods widely utilized in the optimization region, including PSO^4^, ALO^76^, and GWO^77^. The second category features more recently developed algorithms, specifically YDSE^78^ and RIME^79^. To ensure a fair comparison, the parameter settings for all algorithms are adopted from their original publications.

In this study, four single-objective OPF functions are employed to assess the effectiveness and superiority of the SFOA method. These objectives include total fuel cost (FC) ($/h) as defined utilizing Eq. (16), total emissions (EM) (ton/h) as defined utilizing Eq. (17), active power losses (PL) (MW) as defined utilizing Eq. (18), and voltage deviation (VD) (p.u.) as defined utilizing Eq. (19). The control variable values, along with the corresponding best solutions obtained by SFOA, are displayed in Table 13.

**Table 12:** Cases used in this study.

Test system	Case #	Single- and multi-objective functions
IEEE 30-Bus test system	# 1	$\:min{f}_{FC}$
	# 2	$\:min{f}_{EM}$
	# 3	$\:min{f}_{PL}$
	# 4	$\:min{f}_{VD}$
	# 5	$\:min{f}_{FC},{f}_{EM}$
	# 6	$\:min{f}_{FC},{f}_{PL}$
	# 7	$\:min{f}_{FC},{f}_{VD}$
	# 8	$\:min{f}_{FC},{f}_{EM},{f}_{PL}$

**Table 13:** Best control variables obtained by SFOA for cases #(1–4).

Parameters	Bounds		Case			
	Min	Max	#1	#2	#3	#4
Pg1(MW)	50	200	177.0900	64.1733	51.4423	132.4845
Pg2(MW)	20	80	48.4733	67.4145	80.0000	80.0000
Pg5(MW)	15	50	21.5756	50.0000	50.0000	18.5090
Pg8(MW)	10	35	22.4222	35.0000	35.0000	18.6787
Pg11(MW)	10	30	10.6916	30.0000	30.0000	30.0000
Pg13(MW)	12	40	12.0000	40.0000	40.0000	12.0000
Vg1(p.u.)	0.95	1.1	1.0836	1.0492	1.0606	1.0186
Vg2(p.u.)	0.95	1.1	1.0608	1.0419	1.0565	1.0076
Vg5(p.u.)	0.95	1.1	1.0350	1.0211	1.0374	1.0225
Vg8(p.u.)	0.95	1.1	1.0394	1.0338	1.0439	1.0012
Vg11(p.u.)	0.95	1.1	1.0768	1.0634	1.0612	1.0517
Vg13(p.u.)	0.95	1.1	1.0422	1.0557	1.0497	1.0048
Qc10(MVAR)	0	5	4.9723	0.0000	0.2859	4.9923
Qc12(MVAR)	0	5	2.8242	2.3929	0.0000	4.0398
Qc15(MVAR)	0	5	1.9453	5.0000	5.0000	5.0000
Qc17(MVAR)	0	5	1.9125	0.0000	5.0000	0.0000
Qc20(MVAR)	0	5	4.3835	2.8393	5.0000	5.0000
Qc21(MVAR)	0	5	4.1631	5.0000	5.0000	4.0126
Qc23(MVAR)	0	5	4.1402	4.8561	4.9830	4.3984
Qc24(MVAR)	0	5	3.7768	5.0000	4.5894	4.8923
Qc29(MVAR)	0	5	1.8381	1.5197	2.1399	0.3644
T11(p.u.)	0.9	1.1	1.0991	1.0759	0.9972	1.0712
T12(p.u.)	0.9	1.1	0.9000	0.9000	0.9508	0.9064
T15(p.u.)	0.9	1.1	0.9635	1.0055	0.9832	0.9735
T36(p.u.)	0.9	1.1	0.9955	0.9802	0.9739	0.9474
FC ($/h)	-	-	**799.9781**	-	-	
EM (ton/h)	-	-	-	**0.2048**	-	-
PL (MW)	-	-	-	-	**3.0423**	-
VD (p.u.)	-	-	-	-	-	**0.0988**

- Case #1: Minimization of FC.

In this case study, the objective is to minimize the total fuel cost based on the quadratic fuel cost characteristics of the generators. The SFOA method is employed to solve this SOO problem. The optimal control variable values and the corresponding best objective function values obtained by SFOA and its competitor methods are presented in Table 14.

**Fig. 18:**
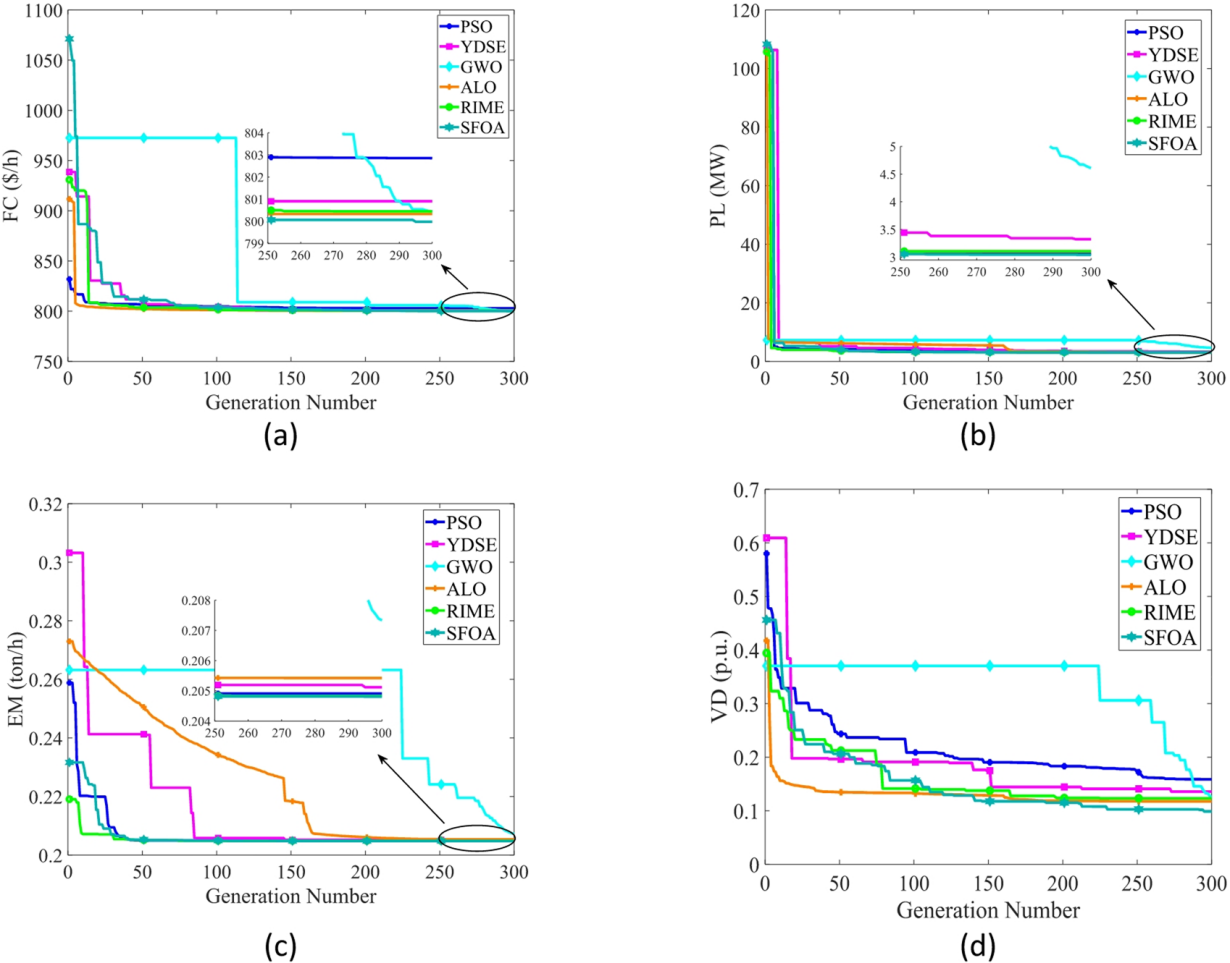
Convergence curves of SFOA and other algorithms for Cases #(1–4). (**a**) Case #1, (**b**) Case #2, (**c**) Case #3, (**d**) Case #4.

As illustrated in Table 14, the SFOA algorithm achieves the lowest fuel cost of 799.9781 ($/h), outperforming all other tested algorithms. In comparison, the fuel costs obtained by the competing methods are 802.8698 ($/h) (PSO), 800.9062 ($/h) (YDSE), 800.4504 ($/h) (GWO), 800.3288 ($/h) (ALO), and 800.4759 ($/h) (RIME). These results clearly demonstrate the superior performance of the introduced SFOA in minimizing the fuel cost objective. Furthermore, the convergence behavior of each approach is depicted in Fig. 18 (a). The figure highlights the rapid and stable convergence of SFOA toward the global optimum compared to the other approaches, further reinforcing its effectiveness in solving this type of optimization problem.

- Case #2: Minimization of EM.

The objective of this case is to address the OPF problem with an emphasis on minimizing the total emissions generated by the power units. The performance of the proposed SFOA algorithm is evaluated and compared against several existing optimization techniques. The simulation outcomes are displayed in Table 15. As in the previous case, the SFOA algorithm outperforms all competing methods, achieving the lowest emission value of 0.2048 (ton/h). In comparison, PSO and RIME yield slightly higher emission values of 0.2049 (ton/h) each. The other algorithms—YDSE, GWO, and ALO—produce emissions of 0.20512, 0.2073, and 0.2054 (ton/h), respectively. These findings demonstrate the effectiveness of SFOA in reducing environmental impact while satisfying OPF constraints.

The convergence behavior of all algorithms for this emission minimization problem is illustrated in Fig. 18 (b). The figure clearly shows that SFOA achieves not only the best final result but also exhibits faster and smoother convergence compared to its counterparts. This performance reflects the algorithm’s robustness, stability, and computational efficiency in addressing complex MOO tasks.

- Case #3: Minimization of PL.

In this scenario, the goal is to minimize active power losses within the power system, a critical factor for improving overall system efficiency and reliability. The developed SFOA algorithm is employed to address this single-objective OPF problem. The corresponding simulation findings, including the optimal control variable settings and their associated power loss values, are described in Table 16. The results reveal that the SFOA method achieves the lowest active power loss, with a value of 3.0423 (MW), outperforming all competing algorithms. This highlights the method’s strong effectiveness in reducing transmission losses and enhancing system performance.

The convergence behavior for this optimization objective is illustrated in Fig. 18 (c). As shown, SFOA exhibits fast and stable convergence toward the optimal solution, highlighting its computational strength and reliability when applied to power loss minimization problems.

- Case #4: Minimization of VD.

The primary objective of this case is to minimize VD across the power network, which is essential for maintaining voltage stability and ensuring reliable system operation. The performance of the introduced SFOA approach is assessed against several benchmark optimization methods, with the findings summarized in Table 17.

The SFOA algorithm achieves the best performance, attaining a minimum voltage deviation of 0.0988 (p.u.), which is significantly lower than the values obtained by other approaches. In comparison, the voltage deviations recorded by PSO, YDSE, GWO, ALO, and RIME are 0.1587 (p.u.), 0.1357 (p.u.), 0.1263 (p.u.), 0.1177 (p.u.), and 0.1233 (p.u.), respectively. These findings clearly demonstrate the superior ability of SFOA to maintain voltage profiles within optimal limits.

The convergence behavior, shown in Fig. 18 (d), further highlights the effectiveness of SFOA. The algorithm not only converges more rapidly but also exhibits greater robustness and stability when minimizing voltage deviation compared to its counterparts, reinforcing its suitability for practical OPF applications.

**Fig. 19:**
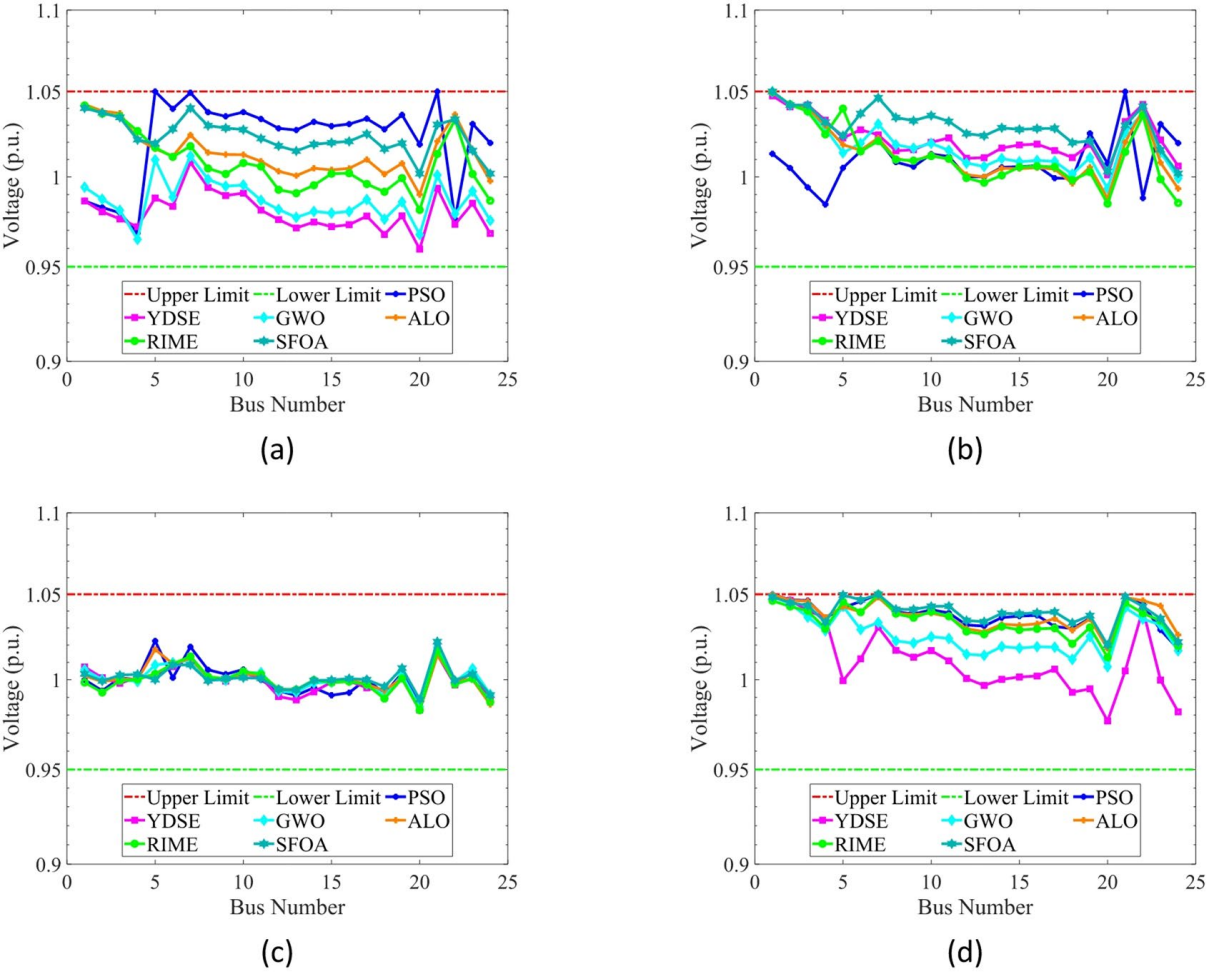
Voltage profile based on all algorithms for Cases #(1–4). (**a**) Case #1, (**b**) Case #2, (**c**) Case #3, (**d**) Case #4.

The bus voltage profiles for Cases 1 to 4 are illustrated in Fig. 19. Both the voltage profiles and the state variables presented in Tables 14, 15, 16 and 17 demonstrate that all algorithms successfully satisfy the system constraints.

**Table 14:** Best control variables obtained by SFOA and other algorithms for case 1.

Parameters	PSO	YDSE	GWO	ALO	RIME	SFOA
Pg1(MW)	178.6030	169.1662	173.3175	176.4936	174.3656	177.0900
Pg2(MW)	49.1863	49.1786	49.1044	48.1385	46.2478	48.4733
Pg5(MW)	21.7056	21.7766	20.9833	21.0719	21.1888	21.5756
Pg8(MW)	21.6794	25.2129	21.3903	19.7848	25.9359	22.4222
Pg11(MW)	10.0000	14.4685	14.9631	14.6150	12.2821	10.6916
Pg13(MW)	12.0000	12.0000	12.3146	12.1597	12.0000	12.0000
Vg1(p.u.)	1.0480	1.0744	1.0834	1.0834	1.0775	1.0836
Vg2(p.u.)	1.0261	1.0621	1.0660	1.0637	1.0582	1.0608
Vg5(p.u.)	0.9896	1.0378	1.0311	1.0327	1.0306	1.0350
Vg8(p.u.)	0.9906	1.0455	1.0370	1.0388	1.0360	1.0394
Vg11(p.u.)	0.9987	1.0398	1.0393	1.0569	1.0625	1.0768
Vg13(p.u.)	1.0525	1.0080	1.0343	1.0211	1.0526	1.0422
Qc10(MVAR)	5.0000	1.8600	2.0380	2.5261	0.0524	4.9723
Qc12(MVAR)	5.0000	2.5473	1.9385	2.4158	3.1865	2.8242
Qc15(MVAR)	0.0000	3.7223	3.6785	1.9899	2.8466	1.9453
Qc17(MVAR)	5.0000	5.0000	2.3153	1.7004	4.2182	1.9125
Qc20(MVAR)	5.0000	4.6053	3.7884	3.5647	4.7359	4.3835
Qc21(MVAR)	5.0000	5.0000	1.7302	2.3224	2.5884	4.1631
Qc23(MVAR)	0.0000	4.5274	2.1332	4.1182	1.1686	4.1402
Qc24(MVAR)	0.0000	3.7494	1.9612	1.1202	4.6045	3.7768
Qc29(MVAR)	0.0000	2.9902	2.7730	2.6802	2.2414	1.8381
T11(p.u.)	0.9987	1.0438	1.0597	1.0526	0.9495	1.0991
T12(p.u.)	0.9000	0.9301	0.9222	0.9563	1.0906	0.9000
T15(p.u.)	1.0127	0.9777	0.9859	0.9890	1.0266	0.9635
T36(p.u.)	0.9000	0.9921	0.9901	0.9968	0.9795	0.9955
FC ($/h)	802.8698	800.9062	800.4504	800.3288	800.4759	**799.9781**

**Table 15:** Best control variables obtained by SFOA and other algorithms for case 2.

Parameters	PSO	YDSE	GWO	ALO	RIME	SFOA
Pg1(MW)	64.2096	66.7984	65.9936	57.8392	64.1147	64.1733
Pg2(MW)	67.7001	65.5334	71.8308	73.7453	67.5643	67.4145
Pg5(MW)	50.0000	50.0000	44.7055	50.0000	50.0000	50.0000
Pg8(MW)	35.0000	35.0000	35.0000	34.9998	35.0000	35.0000
Pg11(MW)	30.0000	30.0000	30.0000	30.0000	30.0000	30.0000
Pg13(MW)	40.0000	40.0000	39.9685	40.0000	40.0000	40.0000
Vg1(p.u.)	1.0001	1.0115	1.0229	1.0571	1.0607	1.0492
Vg2(p.u.)	0.9929	0.9939	1.0037	1.0478	1.0535	1.0419
Vg5(p.u.)	0.9720	0.9853	0.9615	1.0188	1.0324	1.0211
Vg8(p.u.)	0.9771	0.9692	0.9783	1.0328	1.0308	1.0338
Vg11(p.u.)	1.1000	1.0249	1.0231	1.0607	1.0611	1.0634
Vg13(p.u.)	1.0645	1.0244	1.0246	1.0215	1.0167	1.0557
Qc10(MVAR)	0.0000	1.4288	2.1655	3.0459	5.0000	0.0000
Qc12(MVAR)	0.0000	1.1994	3.4279	4.9187	1.4020	2.3929
Qc15(MVAR)	0.0000	3.5964	3.0379	4.9267	1.3598	5.0000
Qc17(MVAR)	0.0000	2.2404	2.9291	4.0258	1.8574	0.0000
Qc20(MVAR)	5.0000	2.0007	2.4238	4.3261	0.2856	2.8393
Qc21(MVAR)	0.0000	0.0228	3.6524	4.9772	5.0000	5.0000
Qc23(MVAR)	5.0000	1.3497	3.6168	4.9776	2.0862	4.8561
Qc24(MVAR)	5.0000	2.2184	2.5878	4.9641	2.0302	5.0000
Qc29(MVAR)	0.0000	3.9298	3.6816	4.9610	2.8403	1.5197
T11(p.u.)	0.9584	1.0137	0.9435	1.0552	1.0501	1.0759
T12(p.u.)	0.9000	0.9378	1.0533	1.0098	0.9654	0.9000
T15(p.u.)	0.9345	0.9571	0.9649	0.9985	0.9887	1.0055
T36(p.u.)	0.9000	0.9570	0.9559	1.0018	0.9958	0.9802
EM (ton/h)	0.2049	0.2051	0.2073	0.2054	0.2049	**0.2048**

**Table 16:** Best control variables obtained by SFOA and other algorithms for case 3.

Parameters	PSO	YDSE	GWO	ALO	RIME	SFOA
Pg1(MW)	51.4585	53.0068	93.2959	52.8972	51.5155	51.4423
Pg2(MW)	80.0000	79.1421	74.5827	79.9895	79.9981	80.0000
Pg5(MW)	50.0000	50.0000	48.3750	50.0000	49.9983	50.0000
Pg8(MW)	35.0000	35.0000	13.1002	33.6567	34.9983	35.0000
Pg11(MW)	30.0000	29.5761	27.9831	29.9990	30.0000	30.0000
Pg13(MW)	40.0000	40.0000	30.6715	39.9606	39.9983	40.0000
Vg1(p.u.)	1.0617	1.0507	1.0653	1.0631	1.0585	1.0606
Vg2(p.u.)	1.0572	1.0509	1.0546	1.0583	1.0509	1.0565
Vg5(p.u.)	1.0391	1.0480	1.0354	1.0406	1.0327	1.0374
Vg8(p.u.)	1.0449	1.0396	1.0377	1.0461	1.0388	1.0439
Vg11(p.u.)	1.1000	0.9916	1.0291	1.0475	1.0719	1.0612
Vg13(p.u.)	1.0515	1.0685	1.0616	1.0396	1.0619	1.0497
Qc10(MVAR)	0.0000	0.4030	1.0672	3.4454	2.3793	0.2859
Qc12(MVAR)	0.1848	4.6158	1.3159	4.9907	1.7062	0.0000
Qc15(MVAR)	4.9972	1.9443	2.2484	4.9997	3.0147	5.0000
Qc17(MVAR)	0.0603	5.0000	2.4959	4.9974	5.0000	5.0000
Qc20(MVAR)	4.2948	0.8460	4.1856	4.6334	4.1046	5.0000
Qc21(MVAR)	4.9956	2.8895	0.8042	4.9957	2.2010	5.0000
Qc23(MVAR)	0.0000	4.7423	4.7139	4.6450	3.5816	4.9830
Qc24(MVAR)	5.0000	1.2690	1.0518	4.9996	0.4478	4.5894
Qc29(MVAR)	0.0000	5.0000	3.2315	4.9894	2.9785	2.1399
T11(p.u.)	1.0682	1.0543	0.9549	0.9992	1.0064	0.9972
T12(p.u.)	0.9000	0.9000	0.9777	0.9887	0.9630	0.9508
T15(p.u.)	0.9792	1.0572	1.0419	0.9778	0.9907	0.9832
T36(p.u.)	0.9663	1.0251	0.9686	0.9852	0.9707	0.9739
PL (MW)	3.0585	3.3251	4.6084	3.1030	3.1084	**3.0423**

**Table 17:** Best control variables obtained by SFOA and other algorithms for case 4.

Parameters	PSO	YDSE	GWO	ALO	RIME	SFOA
Pg1(MW)	81.4922	140.5205	153.7495	113.5472	181.6159	132.4845
Pg2(MW)	80.0000	80.0000	41.6048	55.2638	26.3861	80.0000
Pg5(MW)	50.0000	25.3544	32.2908	32.2434	25.3420	18.5090
Pg8(MW)	34.4905	22.3122	23.5467	27.1526	19.7779	18.6787
Pg11(MW)	29.9868	10.0000	23.3499	26.2416	27.8368	30.0000
Pg13(MW)	12.0000	13.6714	16.5938	35.0474	12.1610	12.0000
Vg1(p.u.)	1.0258	1.0324	1.0280	1.0138	1.0215	1.0186
Vg2(p.u.)	1.0238	1.0161	1.0094	1.0065	1.0034	1.0076
Vg5(p.u.)	1.0177	1.0248	1.0157	1.0180	1.0200	1.0225
Vg8(p.u.)	1.0087	0.9977	1.0018	1.0049	1.0015	1.0012
Vg11(p.u.)	1.0088	0.9982	1.0361	1.0271	1.0566	1.0517
Vg13(p.u.)	0.9977	1.0267	1.0138	1.0121	1.0017	1.0048
Qc10(MVAR)	5.0000	4.7089	3.2971	2.3742	4.7136	4.9923
Qc12(MVAR)	0.0000	5.0000	3.0824	1.6689	2.8222	4.0398
Qc15(MVAR)	0.0000	1.6576	2.5667	2.1044	1.6463	5.0000
Qc17(MVAR)	5.0000	0.7476	2.6041	1.0888	1.4562	0.0000
Qc20(MVAR)	5.0000	1.2724	3.6323	5.0000	4.9022	5.0000
Qc21(MVAR)	0.0000	4.7872	1.0666	2.4856	2.5870	4.0126
Qc23(MVAR)	5.0000	2.3732	4.5343	3.3390	4.4405	4.3984
Qc24(MVAR)	5.0000	4.8831	1.7695	4.9976	0.4094	4.8923
Qc29(MVAR)	0.0000	3.4442	2.6165	1.9913	1.2811	0.3644
T11(p.u.)	0.9270	0.9980	1.0200	0.9751	1.0607	1.0712
T12(p.u.)	1.1000	0.9024	0.9031	0.9443	0.9003	0.9064
T15(p.u.)	0.9000	0.9890	0.9764	0.9695	0.9358	0.9735
T36(p.u.)	0.9470	0.9663	0.9567	0.9617	0.9517	0.9474
VD (p.u.)	0.15867	0.1357	0.1263	0.1177	0.1233	**0.0988**

#### Multi-objective OPF

This subsection provides a set of experimental investigations designed to assess the performance of the introduced MOSFOA approach in addressing bi- and tri-objective OPF problems. Specifically, four test cases (Cases 5–8), summarized in Table 12, are investigated to benchmark the performance of MOSFOA.

To ensure a comprehensive evaluation, the introduced method is compared against a set of well-established and recent MOO approaches, including MOALO, MOAVOA, MOGOA, SPEA-II, and MODA. The quality of solutions is assessed by identifying the BCS using fuzzy set theory^80^, which enables a balanced trade-off between multiple conflicting objectives. Additionally, to preserve diversity among ND solutions and to prevent premature convergence, the CD mechanism is integrated into the MOSFOA framework. This mechanism helps in maintaining a well-distributed PO front by encouraging solutions that are well spread across the objective space.

- Case #5: Minimization of FC and EM.

**Fig. 20:**
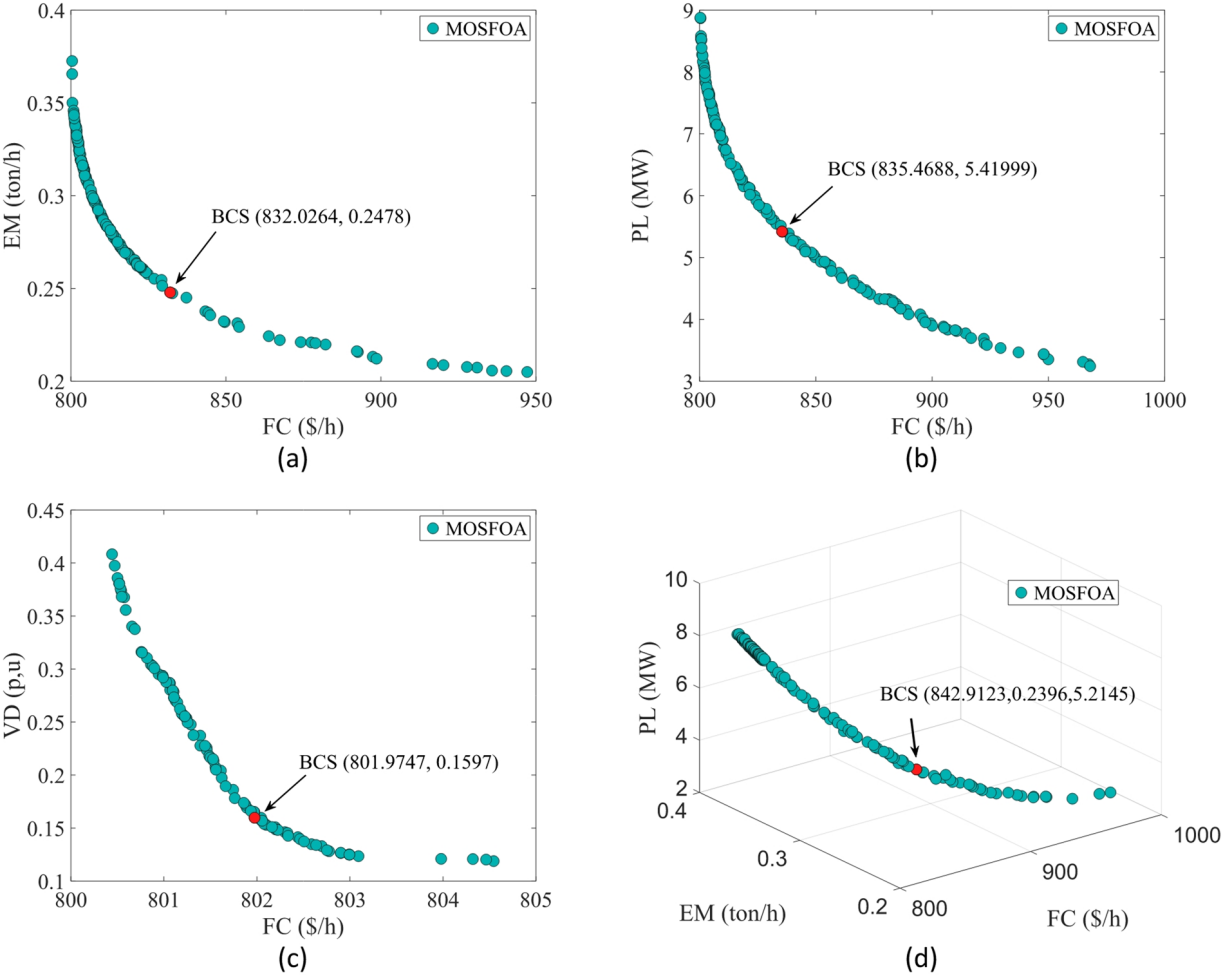
PF obtained by MOSFOA for Cases #(5–8). (**a**) Case #5, (**b**) Case #6, (**c**) Case #7, (**d**) Case #8.

In the fifth case, the optimization process aimed to minimize two conflicting objectives simultaneously: the quadratic FC and pollutant EM. The ND solutions generated by the introduced MOSFOA approach are depicted in Fig. 20 (a). A summary of the BCSs obtained by MOSFOA and other well-known approaches is provided in Table 18. MOSFOA achieved a total FC of 832.0264 ($/h) and an EM level of 0.2478 (ton/h), reflecting a significant improvement in environmental performance compared to other methods. Although MOGOA delivered the lowest total FC, it resulted in the highest EM levels, compromising environmental sustainability. Conversely, SPEA-II achieved the lowest EM but at the expense of a much higher operational cost. Notably, MOSFOA provided a highly competitive solution, outperforming several alternatives in terms of achieving a balanced trade-off.

- Case #6: Minimization of FC and PL.

In this case, the optimization targeted the simultaneous reduction of both FC and active PL. The MOSFOA algorithm is employed to address this multi-objective task, and the resulting PF, depicted in Fig. 20 (b), illustrates a well-distributed set of trade-off solutions across both objectives. Table 18 provides a comparative analysis of the best solutions. MOSFOA achieved an FC of 835.4688 ($/h) with a corresponding active PL of 5.41999 (MW). Although some algorithms, such as MOALO and MOGOA, produced slightly lower FC values, their best solutions failed to satisfy feasibility constraints, making them impractical for implementation. In contrast, MOSFOA not only maintained feasibility but also delivered a balanced and diverse range of solutions. These findings confirm MOSFOA’s robustness and effectiveness in minimizing both operational costs and energy losses concurrently.

- Case #7: Minimization of FC and VD.

This case investigates the MOOPF problem, focusing on the minimization of FC and VD. The PO solutions obtained by the introduced MOSFOA method are illustrated in Fig. 20 (c), showing a well-distributed and diverse PF that effectively captures a wide range of trade-offs between the two objectives. A comparative analysis of MOSFOA’s performance against other methods is shown in Table 18. The findings demonstrate that MOSFOA achieved a balanced performance, obtaining an FC of 801.9747) $/h (and a VD of 0.1597(p.u.) thereby ensuring both economic efficiency and effective voltage regulation.

- Case #8: Minimization of FC and VD.

In the final case, all three objective functions—FC, EM, and active PL—were considered simultaneously. Figure 20 (d) presents the PO solutions obtained by MOSFOA, highlighting the interrelationships and trade-offs among the three objectives. The results confirm that MOSFOA effectively generated a uniformly distributed and well-spread PF, covering a wide range of optimal trade-off solutions.

As depicted, the objectives are inherently conflicting; improvement in one objective often leads to compromises in others. A detailed comparison of the BCS is displayed in Table 18. MOSFOA achieved the best overall performance with an FC of 842.9123 )$/h(, EM of 0.2396 )ton/h(, and active PL of 5.2145 )MW(, demonstrating its strong capability to balance multiple conflicting goals efficiently.

**Fig. 21:**
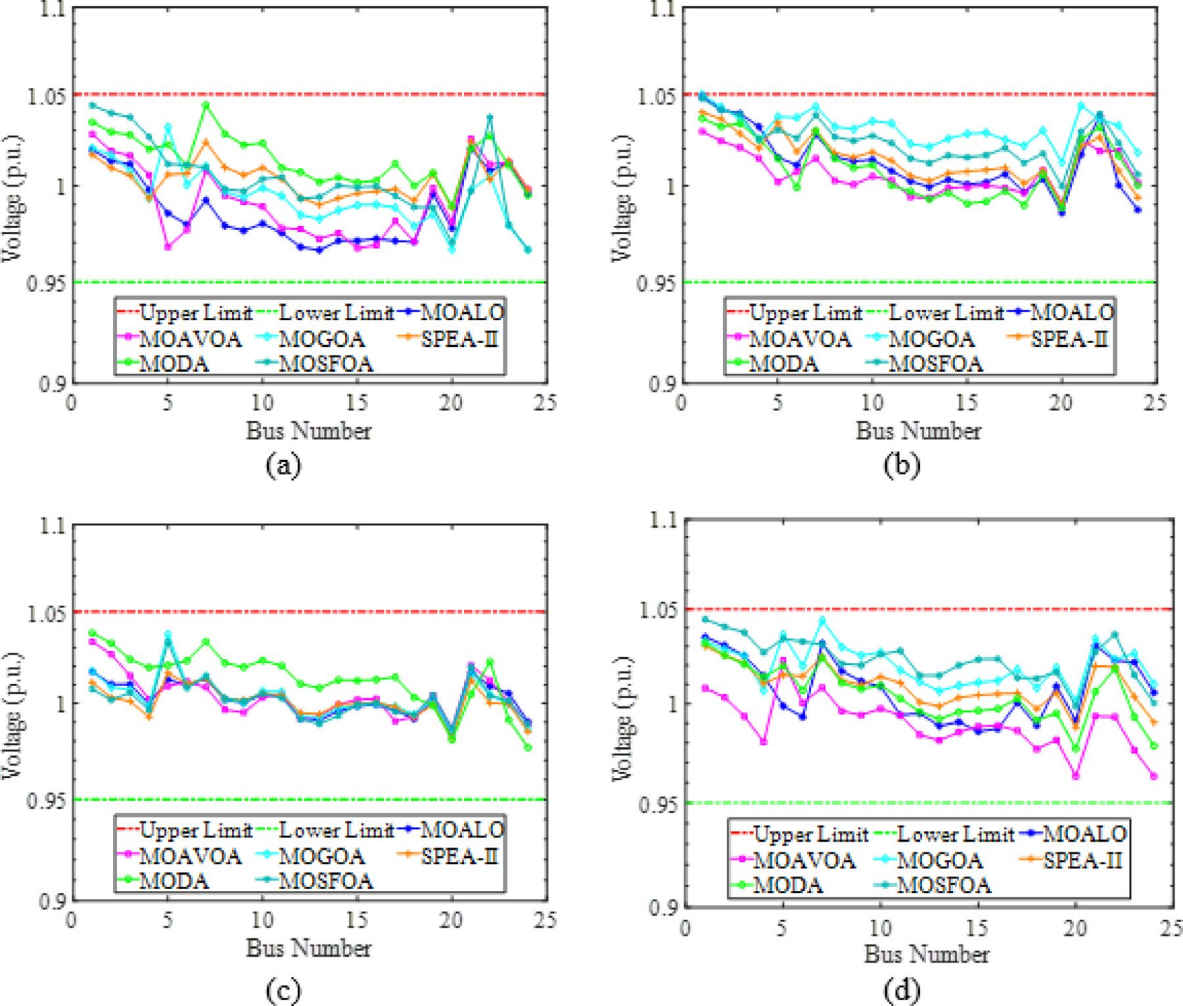
Voltage profile based on all algorithms for Cases #(5–8). (**a**) Case #5, (**b**) Case #6, (**c**) Case #7, (**d**) Case #8.

The values of the control variables, along with the corresponding BCSs obtained by MOSFOA and other methods, are provided in Tables 19, 20, 21 and 22. Additionally, Fig. 21 illustrates the voltage profiles at the load buses for Cases 5 to 8, demonstrating that the voltage levels consistently remain within the specified acceptable range.

**Table 18:** Comparison results of the MOSFOA with other algorithms for cases #(5–8).

Algorithm	Case #								
	#5		#6		#7		# 8		
	FC ($/h)	EM (ton/h)	FC ($/h)	PL (MW)	FC ($/h)	VD (*p*.u.)	FC ($/h)	EM (ton/h)	PL (MW)
MOALO	867.3096	0.2431	832.5362	6.6461	802.6737	0.1957	841.5703	0.2502	6.0115
MOAVOA	836.4208	0.2460	852.1790	5.2350	801.5548	0.2215	845.2618	0.2369	5.7001
MOGOA	817.6664	0.2721	812.8074	6.4347	801.9068	0.1880	836.8839	0.2439	5.6874
SPEA-II	862.1095	0.2249	850.0354	4.9374	802.1080	0.1731	873.5817	0.2197	4.5666
MODA	821.7311	0.2681	843.3198	5.3508	807.2397	0.4277	835.3388	0.2466	5.8335
MOSFOA	832.0264	0.2478	835.4688	5.4199	801.9747	0.1597	842.9123	0.2396	5.2145

**Table 19:** Best control variables obtained by MOSFOA and other algorithms for case 5.

Parameters	MOALO	MOAVOA	MOGOA	SPEA-II	MODA	MOSFOA
Pg1(MW)	108.9652	117.1423	133.5310	87.0718	132.4389	117.2732
Pg2(MW)	67.8638	54.5136	57.5286	63.2282	55.2430	58.3887
Pg5(MW)	45.9245	30.8780	25.6907	33.4993	28.2532	26.3955
Pg8(MW)	28.1756	35.0000	32.7469	34.7265	26.9661	35.0000
Pg11(MW)	23.4128	25.3924	19.9380	29.8184	21.1801	28.2928
Pg13(MW)	14.4653	26.3921	20.7776	39.7847	25.8889	23.7414
Vg1(p.u.)	1.0434	1.0672	1.0331	1.0467	1.0555	1.0597
Vg2(p.u.)	1.0253	1.0453	1.0186	1.0303	1.0422	1.0460
Vg5(p.u.)	0.9972	1.0091	0.9907	0.9943	1.0267	1.0288
Vg8(p.u.)	1.0081	1.01350	1.0054	1.0044	1.0270	1.0400
Vg11(p.u.)	1.0482	0.9751	1.0734	1.0218	1.0297	1.0416
Vg13(p.u.)	1.0046	1.0039	1.0668	1.0255	1.0694	1.0230
Qc10(MVAR)	0.099	2.9151	1.0845	3.2736	2.1235	2.3897
Qc12(MVAR)	4.68836	1.1438	2.3694	1.9024	1.7252	2.4904
Qc15(MVAR)	0.9221	4.9982	0.2866	1.8804	0.0000	0.4538
Qc17(MVAR)	1.8298	4.6588	0.4813	2.8761	1.0130	2.3221
Qc20(MVAR)	4.3785	5.0000	1.5957	0.3899	2.3167	5.0000
Qc21(MVAR)	3.6290	0.3127	3.5570	0.8803	2.3480	0.5556
Qc23(MVAR)	0.7721	2.2936	4.1995	1.7757	2.5874	4.2641
Qc24(MVAR)	3.5880	0.5330	0.3476	2.7031	3.7559	3.8842
Qc29(MVAR)	4.1516	2.3491	0.5344	3.1786	3.5712	0.8731
T11(p.u.)	1.0798	1.0734	0.9586	1.0135	0.9898	1.0523
T12(p.u.)	1.0014	0.9833	1.0608	0.9222	1.0313	0.9281
T15(p.u.)	1.0103	0.9587	1.0763	0.9552	0.9747	1.0338
T36(p.u.)	0.9573	0.9450	0.9791	0.9526	0.9888	1.0177
**FC ($/h)**	867.3096	836.4208	817.6664	862.1095	821.7311	**832.0264**
**EM (ton/h)**	0.2431	0.2460	0.2721	0.2249	0.2681	**0.2478**

**Table 20:** Best control variables obtained by MOSFOA and other algorithms for case 6.

Parameters	MOALO	MOAVOA	MOGOA	SPEA-II	MODA	MOSFOA
Pg1(MW)	143.7462	114.6505	140.0975	94.6690	112.5525	116.3757
Pg2(MW)	51.2508	41.3063	51.6063	57.7704	54.9968	59.3915
Pg5(MW)	41.3746	33.0033	28.2505	41.8780	34.7008	33.3911
Pg8(MW)	17.3254	34.7076	34.9696	34.5574	35.0000	33.9871
Pg11(MW)	13.7843	30.0000	19.4233	28.0265	25.6726	21.9016
Pg13(MW)	22.5110	34.8817	15.4875	30.7543	25.8281	23.7730
Vg1(p.u.)	1.0784	1.0503	1.0787	1.0482	1.0534	1.0737
Vg2(p.u.)	1.0617	1.0378	1.0608	1.0365	1.0449	1.0546
Vg5(p.u.)	1.0398	1.0244	1.0271	1.0112	1.0311	1.0231
Vg8(p.u.)	1.0405	1.0156	1.0386	1.0199	1.0318	1.0375
Vg11(p.u.)	1.0151	1.0236	1.0189	1.0560	1.0501	1.0651
Vg13(p.u.)	1.0253	1.0179	1.0574	1.0378	1.0404	1.0462
Qc10(MVAR)	3.7180	2.6462	0.7166	1.6778	0.3464	1.1216
Qc12(MVAR)	4.8742	4.2460	3.0297	1.7460	2.9194	0.5975
Qc15(MVAR)	4.1410	0.1279	3.9799	3.2670	1.8325	2.1921
Qc17(MVAR)	2.5618	2.9358	4.8424	2.5060	5.0000	3.8906
Qc20(MVAR)	3.4658	5.0000	2.5506	3.0337	5.0000	3.0278
Qc21(MVAR)	1.3713	4.7710	4.4373	2.8099	3.4210	0.1876
Qc23(MVAR)	3.3418	3.3873	1.5153	3.5436	0.0000	4.3496
Qc24(MVAR)	3.3481	4.9997	4.0589	3.5110	3.0009	5.0000
Qc29(MVAR)	1.2003	4.6239	2.8419	2.0251	3.6566	4.6210
T11(p.u.)	1.0138	1.0486	0.9784	1.0047	1.0227	1.0325
T12(p.u.)	0.9882	0.9364	0.9407	0.9491	1.1000	0.9626
T15(p.u.)	0.9866	0.9967	1.0021	1.0097	0.9734	0.9851
T36(p.u.)	0.9953	0.9740	0.9738	0.9750	0.9799	0.9983
FC ($/h)	832.5362	852.1790	812.8074	850.0354	843.3198	**835.4688**
PL (MW)	6.6461	5.2350	6.4347	4.9374	5.3508	**5.4199**

**Table 21:** Best control variables obtained by MOSFOA and other algorithms for case 7.

Parameters	MOALO	MOAVOA	MOGOA	SPEA-II	MODA	MOSFOA
Pg1(MW)	173.1620	176.9087	177.0784	176.7943	165.6220	177.5189
Pg2(MW)	46.4543	48.8942	49.6096	47.2350	47.9484	48.6649
Pg5(MW)	19.9454	20.9384	21.4610	21.5998	22.5066	20.5899
Pg8(MW)	28.6489	22.3441	20.8834	20.4733	17.5794	20.4288
Pg11(MW)	10.1547	10.8940	11.7046	14.1553	13.0550	11.9836
Pg13(MW)	14.1597	12.7250	12.1109	12.4806	25.2521	13.6668
Vg1(p.u.)	1.0456	1.0618	1.0552	1.0493	1.0608	1.0625
Vg2(p.u.)	1.0282	1.0395	1.0352	1.0302	1.0438	1.0338
Vg5(p.u.)	1.0036	1.0028	1.0058	0.9945	1.0312	0.9996
Vg8(p.u.)	1.0121	1.0159	1.0031	0.9994	1.0160	1.0027
Vg11(p.u.)	1.0573	1.0026	1.0828	1.0433	1.0380	1.0524
Vg13(p.u.)	1.0145	1.0524	1.0062	1.0383	1.0699	1.0202
Qc10(MVAR)	2.8029	1.0944	2.9027	1.7820	2.5028	4.4816
Qc12(MVAR)	0.0075	1.7285	2.2767	2.6450	0.0217	3.9235
Qc15(MVAR)	2.9642	0.4785	0.1807	2.1377	3.1308	3.4003
Qc17(MVAR)	2.0290	2.0581	4.8769	2.3353	4.8247	0.5095
Qc20(MVAR)	1.9733	4.6350	2.9822	2.2466	2.9187	4.4964
Qc21(MVAR)	0.7693	4.6217	0.5614	4.2199	2.4341	4.7114
Qc23(MVAR)	2.8936	0.0579	3.9408	2.7547	4.6053	4.5354
Qc24(MVAR)	3.0647	4.0819	4.9009	3.5077	3.8480	1.7931
Qc29(MVAR)	2.7827	0.0000	1.5332	2.8634	2.1854	2.0581
T11(p.u.)	1.0351	1.0014	0.9626	1.0122	1.0234	1.0720
T12(p.u.)	0.9181	0.9037	1.0579	0.9375	0.9207	0.9021
T15(p.u.)	0.9713	1.0755	0.9552	1.0101	1.0363	0.9996
T36(p.u.)	0.9713	0.9600	0.9665	0.9652	1.0060	0.9700
FC ($/h)	802.6737	801.5548	801.9068	802.1080	807.2397	**801.9747**
VD (p.u.)	0.1957	0.2215	0.1880	0.1731	0.4277	**0.1597**

**Table 22:** Best control variables obtained by MOSFOA and other algorithms for case 8.

Parameters	MOALO	MOAVOA	MOGOA	SPEA-II	MODA	MOSFOA
Pg1(MW)	122.7069	109.8343	113.6568	92.5459	114.8682	111.0017
Pg2(MW)	57.5313	56.4673	60.5348	64.5031	62.4880	59.3766
Pg5(MW)	32.4216	29.3492	30.0024	37.6006	30.4587	33.5415
Pg8(MW)	26.4546	34.1061	34.8534	34.1823	35.0000	32.5186
Pg11(MW)	15.9109	29.9079	21.9917	28.4023	20.8023	23.9862
Pg13(MW)	34.5969	29.4354	27.9502	30.6002	25.5220	28.1899
Vg1(p.u.)	1.0510	1.0270	1.0494	1.0492	1.0580	1.0589
Vg2(p.u.)	1.0427	1.0054	1.0383	1.0407	1.0350	1.0452
Vg5(p.u.)	1.0177	0.9804	0.9991	1.0132	1.0221	1.0295
Vg8(p.u.)	1.0239	0.9993	1.0205	1.0199	1.0129	1.0345
Vg11(p.u.)	0.9893	1.0096	1.0907	1.0390	1.0710	1.0890
Vg13(p.u.)	1.0678	1.0515	1.0680	1.0329	1.0450	1.0469
Qc10(MVAR)	1.4787	2.1979	2.6743	1.0135	1.7732	0.0000
Qc12(MVAR)	2.1097	2.8184	4.8364	4.4408	2.5406	0.4010
Qc15(MVAR)	3.7182	4.6343	1.4962	1.3521	1.7529	5.0000
Qc17(MVAR)	3.1180	0.2064	1.7484	3.5303	0.7971	4.6054
Qc20(MVAR)	3.3672	0.4185	0.7662	2.6435	1.3651	3.4385
Qc21(MVAR)	4.0751	1.2861	3.7140	3.3218	1.2718	5.0000
Qc23(MVAR)	2.0620	2.8092	3.0971	4.1935	4.9153	0.0000
Qc24(MVAR)	2.1723	0.5805	2.8614	2.0936	3.5103	5.0000
Qc29(MVAR)	3.6643	0.9815	3.9742	1.2759	2.3661	2.5097
T11(p.u.)	1.0045	0.9192	1.0097	1.0266	1.0255	1.0536
T12(p.u.)	1.0516	0.9831	1.0384	0.9362	0.9910	0.9275
T15(p.u.)	1.0146	1.0493	0.9907	0.9926	1.0039	1.0206
T36(p.u.)	0.9626	0.9719	0.9702	0.9715	0.9930	0.9912
**FC ($/h)**	841.5703	845.2618	836.8839	873.5817	835.3388	**842.9123**
**EM (ton/h)**	0.2502	0.2369	0.2439	0.2197	0.2466	**0.2396**
**PL (MW)**	6.0115	5.7001	5.6874	4.5666	5.8335	**5.2145**

**Table 23:** Results of HV metric of different MOO algorithms on OPF for cases #(5–8).

Problem	Index ↓	Algorithms					
		MOALO	MOAVOA	MOGOA	SPEA-II	MODA	MOSFOA
Case #5: FC + EM	Ave	1.127E-01	1.176E-01	1.180E-01	1.136E-01	1.154E-01	1.198E-01
	SD	1.461E-02	1.495E-03	2.346E-03	2.834E-03	3.647E-03	7.518E-04
Case #6: FC + PL	Ave	1.243E-01	1.405E-01	1.499E-01	1.413E-01	1.432E-01	1.515E-01
	SD	2.366E-03	4.947E-03	2.444E-03	3.099E-03	4.238E-03	1.036E-03
Case #7: FC + VD	Ave	7.874E-02	8.133E-02	8.437E-02	7.980E-02	8.057E-02	8.465E-02
	SD	1.241E-03	1.507E-03	1.571E-03	1.151E-03	4.528E-03	1.250E-03
Case #8: FC + EM + PL	Ave	5.392E-02	6.885E-02	6.926E-02	7.153E-02	6.786E-02	7.286E-02
	SD	7.692E-03	5.313E-03	1.787E-03	1.097E-03	3.052E-03	1.887E-03

#### Stability analysis

**Fig. 22:**
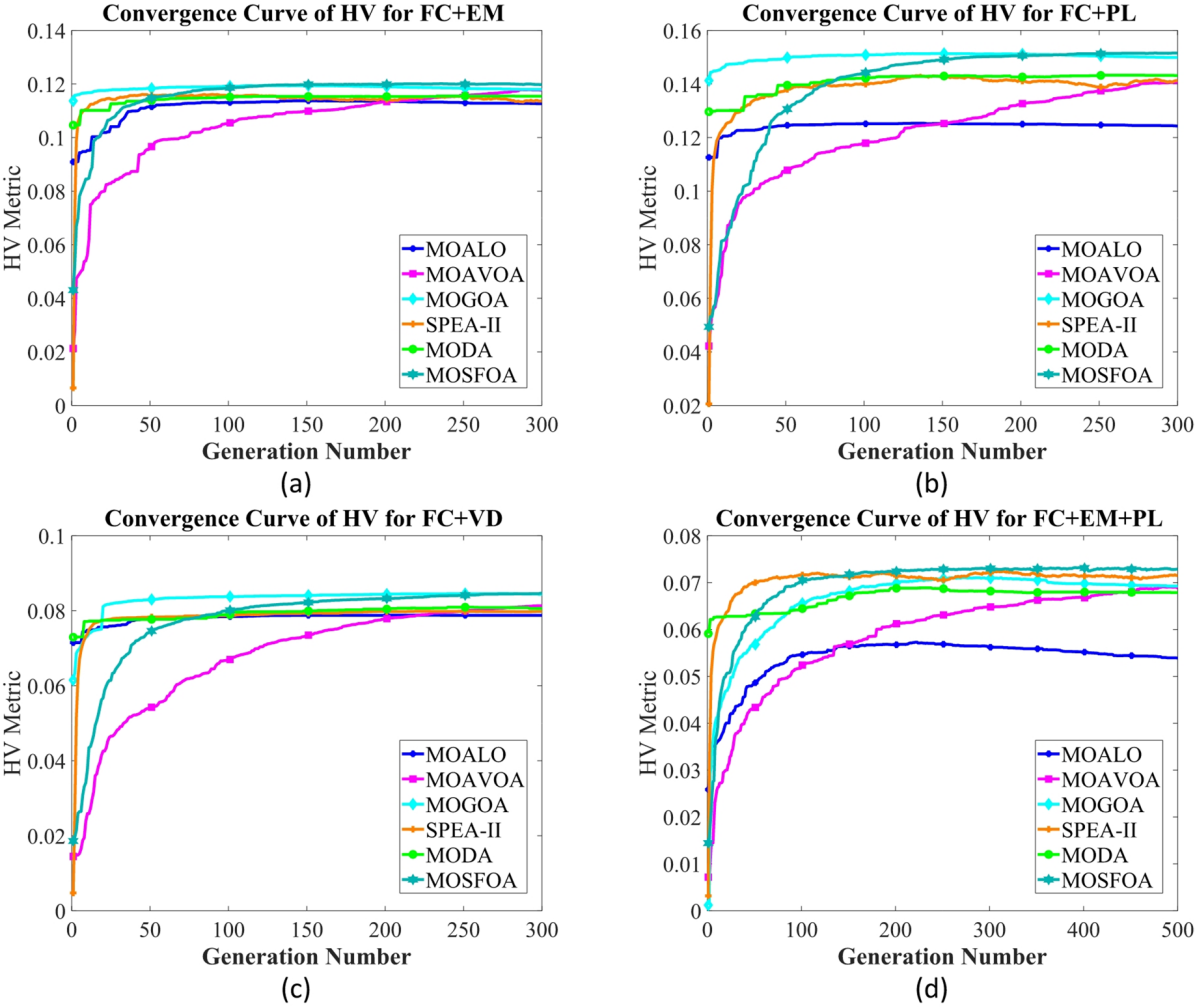
Convergence curve performance of algorithms for HV metric. (**a**) Case #5, (**b**) Case #6, (**c**) Case #7, (**d**) Case #8.

**Fig. 23:**
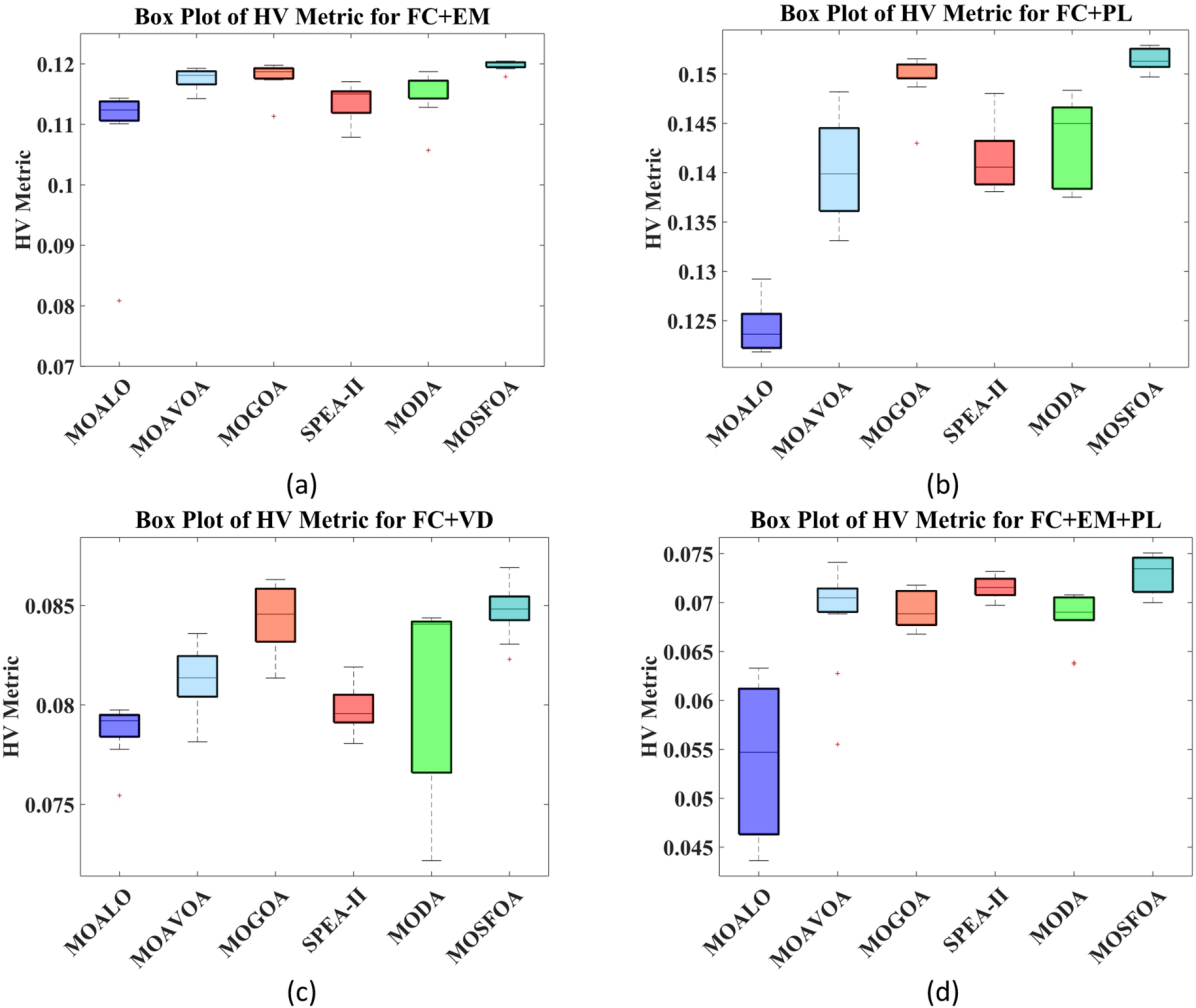
Box plot performance of algorithms for HV metric. (**a**) Case #5, (**b**) Case #6, (**c**) Case #7, (**d**) Case #8.

Table 23 presents the performance of various approaches, including MOSFOA, across a range of MOOPF problems, evaluated using the HV metric. The results clearly show that MOSFOA consistently delivers outstanding performance, frequently achieving the highest HV values. Since a higher HV value indicates better convergence to the PF and greater solution diversity, this confirms the algorithm’s strong effectiveness in MOO. For instance, in Case 6 (FC + PL), MOSFOA achieves an HV of 0.1515, outperforming MOLOA (0.1423), MOAVOA (0.1405), MOGOA (0.1499), SPEA-II (0.1431), and MODA (0.1432). This trend of superior HV values is consistent across several complex cases, confirming MOSFOA’s capability to find diverse and well-converged solution sets. Furthermore, Fig. 22 illustrates MOSFOA’s ability to maintain a strong balance between convergence and diversity in MOO optimization scenarios—an essential feature for robust decision-making in complex MOOPF problems. The boxplot in Fig. 23 further confirms that MOSFOA consistently delivers high HV values, showcasing its reliability and stable performance across different test scenarios.

**Fig. 24:**
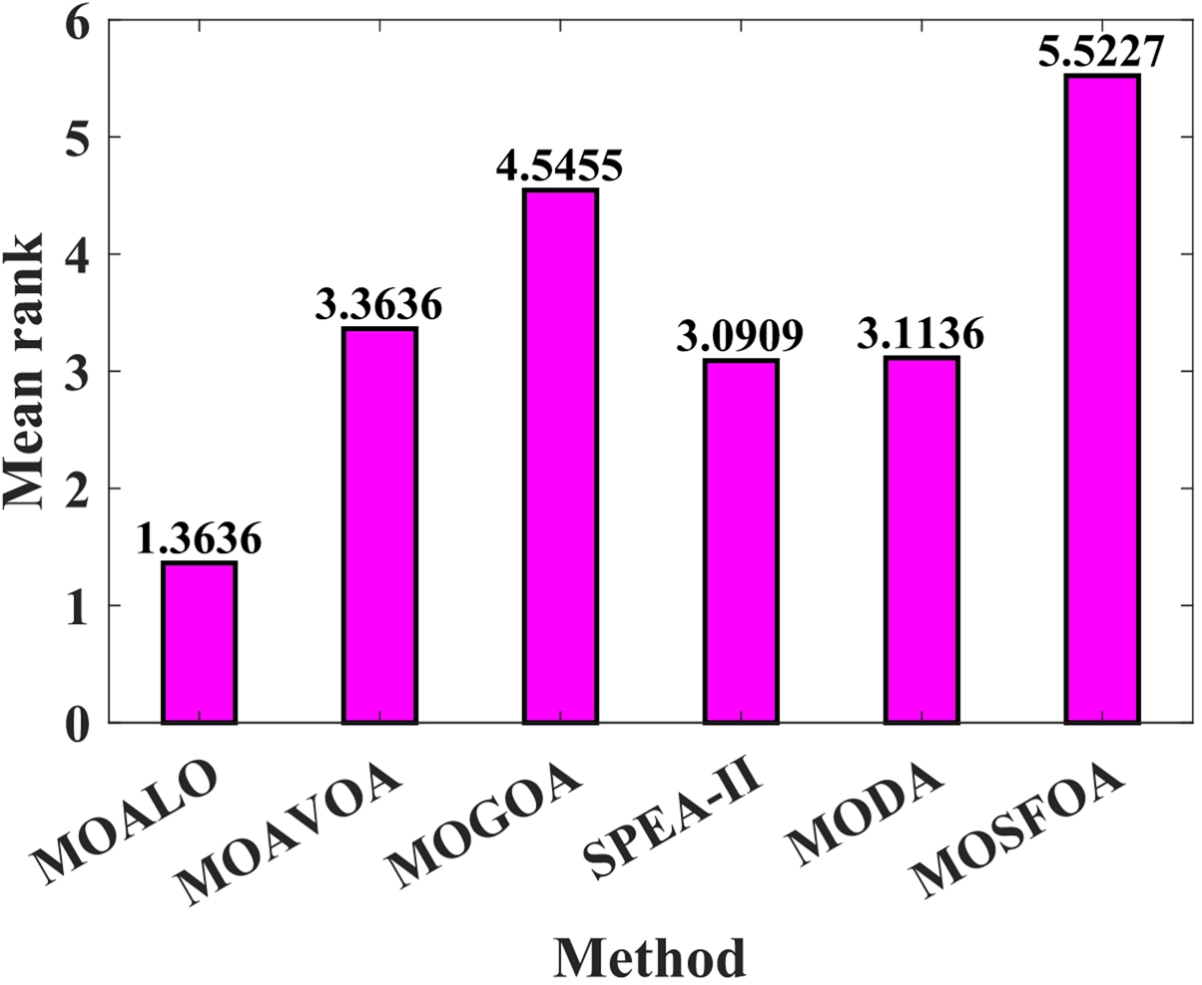
Mean friedman rank of HV for all algorithms.

Figure 24 presents the Friedman test results, offering a statistical comparison of the algorithms. These results reaffirm MOSFOA’s dominance, as it consistently ranks first in terms of HV across the MOOPF functions, highlighting its superiority over competing algorithms. In sum, the HV results and statistical rankings confirm that MOSFOA is a robust and effective algorithm for many-objective optimization, delivering high-quality, diverse, and convergent solutions across a broad range of MOOPF challenges.

### Real-world multi-objective optimization of a speed reducer design for industrial applications

**Fig. 25:**
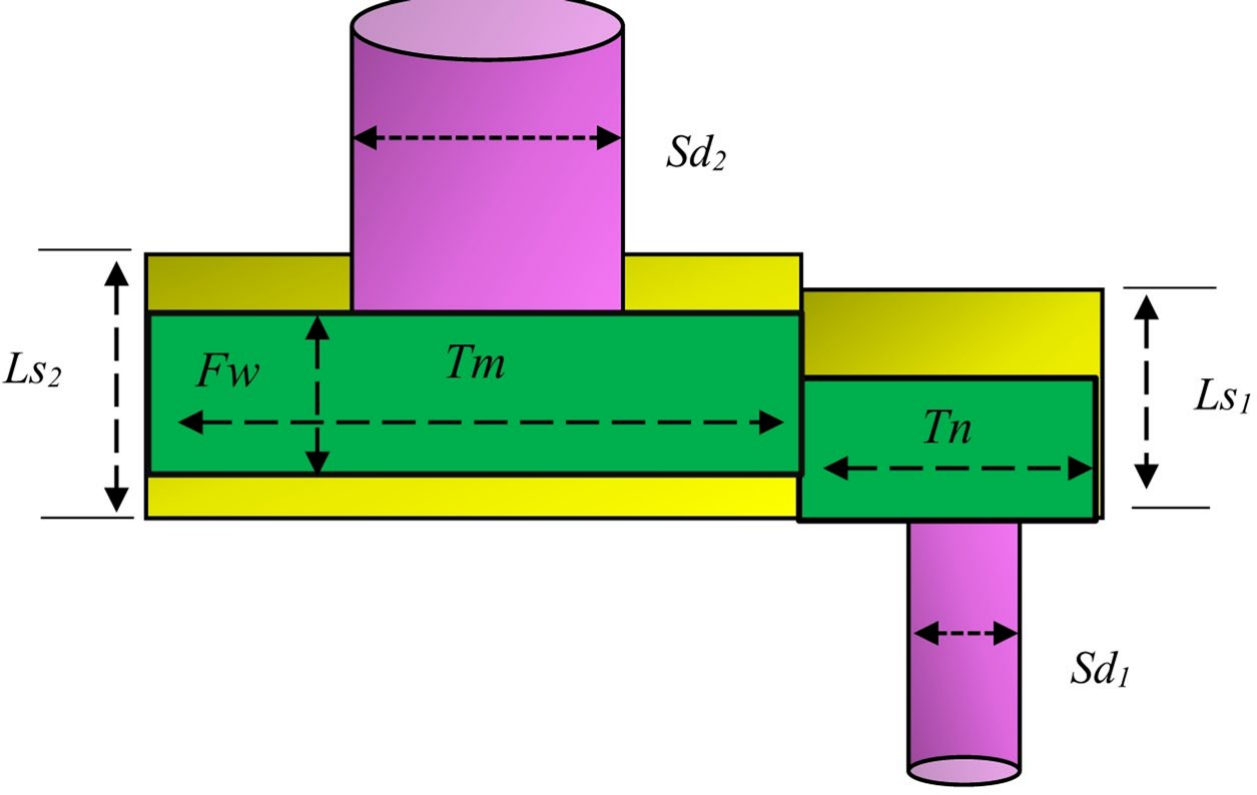
The structure of speed reducer problem.

The speed reducer design problem is a significant real-world application in mechanical engineering and industrial design. It offers a rigorous benchmark for evaluating multi-objective optimization algorithms by examining their ability to balance structural performance, mechanical reliability, and material efficiency. The problem involves seven design variables, $\:X\:=\:[{x}_{1},\:{x}_{1},\:{x}_{3},\:{x}_{4},\:{x}_{5},\:{x}_{6},\:{x}_{7}]$, as illustrated in Fig. 25, representing the face width $\:Fw\:$($\:{=x}_{1}$), module of the teeth $\:Tm$ ($\:={x}_{2}$), number of teeth on the pinion $\:Tn$ ($\:{=x}_{3}$), length of the first shaft between bearings $\:{Ls}_{1}\:$($\:{=x}_{4}$), length of the second shaft between bearings $\:{Ls}_{2}$ ($\:={x}_{5}$), diameter of the first shaft $\:\:{Sd}_{1}\:$($\:={x}_{6}$), and diameter of the second shaft $\:{Sd}_{2}\:$($\:{=x}_{7}$). It is formulated as a dual-objective minimization problem, as shown in Eq. (18), aiming to reduce the overall volume (f1) and minimize mechanical stress (f2). The resulting optimization problem is structured as follows:

39 $$\:Minimize\::\left\{\begin{array}{c}\begin{array}{c}\text{}\text{}{f}_{1}=0.7854{x}_{1}{x}_{2}^{2}\left(3.3333{x}_{3}^{2}+14.9334{x}_{3}-43.0934\right)\\\:-1.508{x}_{1}\left({x}_{6}^{2}+{x}_{7}^{2}\right)+7.4777\left({x}_{6}^{3}+{x}_{7}^{3}\right)+0.7854\left({x}_{4}{x}_{6}^{2}+{x}_{5}{x}_{7}^{2}\right)\\\:\text{}\text{}\end{array}\\\:{f}_{2}=\frac{\sqrt{{\left(745\frac{{x}_{4}}{{x}_{2}{x}_{3}}\right)}^{2}+1.69e7}}{0.1*{x}_{6}^{3}}\end{array}\right.$$

Subject to:

40 $$\begin{gathered} \left\{ {\begin{array}{*{20}c} {g_{1} = - \left( {27/\left( {x_{1} x_{2}^{2} x_{3} } \right) - 1} \right)} \\ {\:g_{2} = - \left( {397.5/\left( {x_{1} x_{2}^{2} x_{3}^{3} } \right) - 1} \right)} \\ {\:g_{3} = - \left( {1.93\frac{{x_{4}^{3} }}{{x_{2} x_{3} x_{6}^{4} }} - 1} \right)} \\ {\:g_{4} = - \left( {1.93\frac{{x_{5}^{3} }}{{x_{2} x_{3} x_{7}^{4} }} - 1} \right)} \\ {\:g_{5} = - \left( {\frac{{\sqrt {\left( {745\frac{{x_{4} }}{{x_{2} x_{3} }}} \right)^{2} + 16.9e6} }}{{0.1x_{6}^{3} }} - 1} \right)} \\ {\:g_{6} = - \left( {\frac{{\sqrt {\left( {745\frac{{x_{5} }}{{x_{2} x_{3} }}} \right)^{2} + 157.5e6} }}{{0.1x_{7}^{3} }} - 1} \right)} \\ {\:g_{7} = - \left( {x_{2} *\frac{{x_{3} }}{{40}} - 1} \right)} \\ {\:g_{8} = - \left( {5*\frac{{x_{2} }}{{x_{1} }} - 1} \right)} \\ {\:g_{9} = - \left( {\frac{{x_{1} }}{{12*x_{2} }} - 1} \right)} \\ {\:g_{{10}} = - \left( {\frac{{1.5*x_{6} + 1.9}}{{x_{4} }} - 1} \right)} \\ {\:g_{{11}} = - \left( {\frac{{1.1*x_{7} + 1.9}}{{x_{5} }} - 1} \right)} \\ \end{array} } \right. \hfill \\ 2.6 \le \:x_{1} \le \:{\mathrm{3}}{\mathrm{.6,0}}{\mathrm{.7}} \le \:x_{2} \le \:{\mathrm{0}}{\mathrm{.8,17}} \le \:x_{3} \le \:{\mathrm{28,7}}{\mathrm{.3}} \le \:x_{4} ,x_{5} \le \:8.3, \hfill \\ 2.9 \le \:x_{6} \le \:{\mathrm{3}}{\mathrm{.9,5}} \le \:x_{7} \le \:5.5 \hfill \\ \end{gathered}$$

**Fig. 26:**
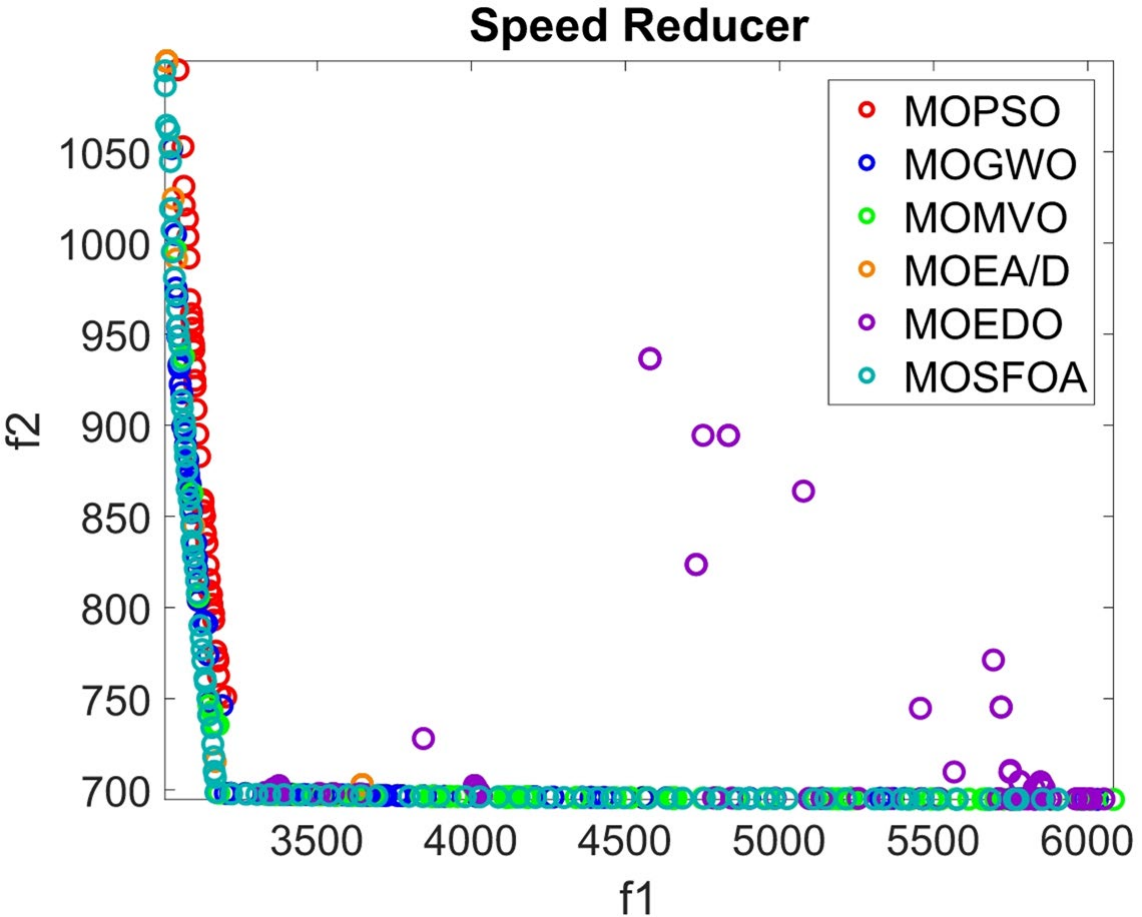
Non-dominated solutions obtained by MOSFOA and competitive algorithms.

Table 24 presents the HV performance of the evaluated multi-objective algorithms on the speed reducer design problem, revealing clear distinctions in convergence quality and solution diversity. MOSFOA demonstrates the strongest overall performance, achieving the highest Best HV value (2.268E − 01) and the highest average HV (2.266E − 01), along with an exceptionally low standard deviation (2.222E − 04). These results indicate that MOSFOA not only converges closer to the true Pareto front but also provides highly stable performance across multiple runs. Although MOGWO and MOAVOA also perform competitively—with Best HV values of 2.258E − 01 and 2.253E − 01, respectively—their slightly lower averages and higher variances reflect reduced consistency. In contrast, algorithms such as MOPSO, MOEA/D, and MOEDO yield noticeably lower HV values, indicating weaker convergence behavior and poorer solution diversity. Moreover, the non-dominated solutions displayed in Fig. 26 further confirm that the developed MOSFOA algorithm outperforms the compared methods in terms of both convergence and distribution of solutions. Overall, the consistently superior HV results validate MOSFOA’s effectiveness in generating well-distributed and highly converged Pareto-optimal solutions for the speed reducer design problem.

**Table 24:** Results of HV metric of different MOO algorithms on speed reducer problem.

Problem	Algorithm	HV		
		Best	Ave	SD
Speed reducer design problem	MOPSO	2.005E-01	1.098E-01	1.016E-01
	MOGWO	2.258E-01	2.235E-01	2.179E-03
	MOAVOA	2.253E-01	2.249E-01	4.386E-04
	MOEA/D	2.214E-01	2.071E-01	1.803E-02
	MOEDO	2.112E-01	2.103E-01	1.098E-03
	MOSFOA	**2.268E-01**	**2.266E-01**	**2.222E-04**

### Comprehensive analysis of experimental results

As discussed earlier, the standard SFOA has shown strong performance in solving a variety of real-world optimization problems. In this study, we extend SFOA to the multi-objective domain by introducing the Multi-Objective Sailfish Optimization Algorithm (MOSFOA). MOO algorithms encounter two primary challenges: (i) attaining precise convergence toward the global Pareto front (PF), and (ii) preserving a well-spread distribution of nondominated solutions. Therefore, an efficient MOO algorithm must maintain a proper balance between convergence and diversity.

To evaluate the performance of MOSFOA, we conduct extensive experiments on unconstrained benchmark functions, constrained problems, and real-world engineering applications. For unconstrained benchmarks, we use ten widely recognized test functions from the ZDT and DTLZ suites, each with different characteristics as summarized in Table 1. MOSFOA is compared against five well-known MOO approaches: MOPSO, MOGWO, MOMVO, MOEA/D, and MOEDO. Two standard performance indicators are employed: the IGD, which assesses convergence and distribution, and the HV, which simultaneously measures convergence and spread.

The findings show that MOSFOA outperformed the competing methods in eight out of ten test cases for the IGD metric and nine out of ten test cases for the HV metric, demonstrating its strong capability to handle diverse unconstrained MOPs. For constrained and engineering problems, nine benchmark functions are used, including BNH, SRN, OSY, TNK, Car Side Impact, Disk Brake, 4-Bar Truss, CONSTR, and Welded Beam. MOSFOA again achieved superior performance, ranking first in five out of nine problems for IGD and in six out of nine problems for HV, confirming its effectiveness in maintaining a solid balance between convergence and diversity in more complex engineering environments.

To further assess convergence behavior, we employ the KKTPM metric. The results show that MOSFOA ranked first in four out of eight benchmark cases and achieved competitive performance in the remaining problems, reaffirming its reliability in approximating Pareto-optimal solutions. Moreover, MOSFOA was also evaluated on the real-world speed reducer design problem, where it achieved strong and well-distributed Pareto fronts, further demonstrating its capability in solving practical industrial optimization tasks. Additionally, MOSFOA was applied to the OPF problem for the IEEE 30-bus system under single-, bi-, and tri-objective formulations. In all cases, MOSFOA consistently outperformed the competing methods, delivering improved convergence and high-quality nondominated solutions, highlighting its robustness for many-objective OPF applications.

From all the above results, it is evident that the proposed MOSFOA algorithm is strong, stable, and highly effective across various problem classes. More specifically, MOSFOA is particularly suitable for:

- Complex engineering MOPs requiring a careful balance between convergence and diversity.
- Nonlinear, multimodal, or discontinuous search spaces.
- Situations where gradient information is unavailable or unreliable.
- Applications that require both strong global exploration and precise local refinement—core strengths inherited from the original SFOA.

Finally, based on the key challenges outlined in Sect. 2 and the strong empirical performance demonstrated across all experiments, we conclude that MOSFOA successfully addresses the main limitations of existing MOEAs. It provides superior convergence, maintains solution diversity, and consistently delivers high-quality Pareto-optimal sets across benchmark and real-world optimization problems.

## Conclusion and future work

This study introduced MOSFOA, a novel multi-objective extension of SFOA that incorporates NDS and CD mechanisms to preserve both convergence and diversity. The algorithm was validated on ten benchmark test cases from the ZDT and DTLZ suites, representing a wide range of Pareto front and Pareto set characteristics. In addition, MOSFOA was evaluated on nine well-known constrained engineering design problems, the OPF problem on the IEEE 30-bus system, and a real-world multi-objective speed reducer design application. Key findings are:

- MOSFOA achieves superior convergence and diversity compared with leading multi-objective algorithms.
- In OPF studies, both SFOA and MOSFOA effectively handle economic, environmental, and technical objectives.
- Single-objective evaluations confirm SFOA’s competitiveness against well-known optimizers.
- Statistical validation using the Wilcoxon rank-sum and Friedman tests, supported by box plot analyses of IGD, HV, and KKTPM metrics, confirms the significance, robustness, and stability of the proposed methods across diverse benchmark and real-world optimization problems.

The results demonstrate that MOSFOA significantly outperforms its counterparts, achieving improvements of up to 68.42% in IGD and 78.95% in HV, highlighting its ability to achieve a well-balanced trade-off between convergence and diversity. Overall, MOSFOA and SFOA demonstrate strong potential for addressing both single- and multi-objective optimization problems in complex engineering scenarios.

In future research, we plan to further evaluate the effectiveness of MOSFOA in complex multi-objective real-world engineering problems, with the aim of continuously enhancing its optimization capabilities and expanding its practical applicability. Specifically, we intend to investigate its performance on advanced multi-objective problems such as wind power forecasting, cybersecurity optimization, optimal reactive power dispatch, mobile cell frequency allocation, and radar placement optimization, among others. These studies will help demonstrate MOSFOA’s ability to balance competing objectives while maintaining convergence and diversity across high-dimensional decision spaces. Beyond practical applications, we also aim to strengthen the theoretical foundation of MOSFOA by exploring its internal dynamics using advanced analytical frameworks, such as complex network theory, as suggested in recent studies^81,82^. This perspective could help reveal the information flow, interaction patterns, and convergence behavior within the algorithm, ultimately contributing to a deeper understanding of its exploration–exploitation balance and improving its overall robustness and scalability.

## Data Availability

The data presented in this study are available through email upon request to the corresponding author.
